# Sirtuin 6 deficiency induces endothelial cell senescence via downregulation of forkhead box M1 expression

**DOI:** 10.18632/aging.202176

**Published:** 2020-11-10

**Authors:** Ok-Hee Lee, Yun Mi Woo, Sohyeon Moon, Jihyun Lee, Haeun Park, Hoon Jang, Yun-Yong Park, Soo-Kyung Bae, Keun-Hong Park, Ji Hoe Heo, Youngsok Choi

**Affiliations:** 1 Department of Biomedical Science, CHA University, Seongnam-si 13488, Gyeonggi-do, Republic of Korea; 2 Department of Stem Cell and Regenerative Biotechnology, Konkuk University, Seoul 05029, Republic of Korea; 3 Asan Institute for Life Sciences, Asan Medical Center, Seoul 05505, Republic of Korea; 4 Department of Dental Pharmacology, BK21 PLUS Project, School of Dentistry, Pusan National University, Yangsan 50612, Republic of Korea; 5 Department of Neurology, Severance Hospital, Yonsei University College of Medicine, Seoul 03722, Republic of Korea; 6 Department of Life Science, Jeonbuk National University, Jeonju-si 54896, Jeollabuk-do, Republic of Korea

**Keywords:** SIRT6, FOXM1, endothelial cell, senescence, cell cycle

## Abstract

Cellular senescence of endothelial cells causes vascular dysfunction, promotes atherosclerosis, and contributes to the development of age-related vascular diseases. Sirtuin 6 (SIRT6), a conserved NAD+-dependent protein deacetylase, has beneficial effects against aging, despite the fact that its functional mechanisms are largely uncharacterized. Here, we show that SIRT6 protects endothelial cells from senescence. SIRT6 expression is progressively decreased during both oxidative stress-induced senescence and replicative senescence. SIRT6 deficiency leads to endothelial dysfunction, growth arrest, and premature senescence. Using genetically engineered endothelial cell-specific SIRT6 knockout mice, we also show that down-regulation of SIRT6 expression in endothelial cells exacerbates vascular aging. Expression microarray analysis demonstrated that SIRT6 modulates the expression of multiple genes involved in cell cycle regulation. Specifically, SIRT6 appears to regulate the expression of forkhead box M1 (FOXM1), a critical transcription factor for cell cycle progression and senescence. Overexpression of FOXM1 ameliorates SIRT6 deficiency-induced endothelial cell senescence. In this work, we demonstrate the role of SIRT6 as an anti-aging factor in the vasculature. These data may provide the basis for future novel therapeutic approaches against age-related vascular disorders.

## INTRODUCTION

Vascular aging is accompanied by dysfunctional vascular phenotypes including endothelial dysfunction and arterial stiffening that play critical causal roles in the majority of cardiovascular diseases [1–3]. Cellular senescence, an irreversible cell cycle arrest, in endothelial cells critically contributes to this vascular aging. Senescent endothelial cells show impaired homeostatic functions including nitric oxide production, vascular inflammation, cytoskeleton integrity, angiogenesis, proliferation, and cell migration [4, 5]. Senescent cells can also propagate their senescence phenotypes to healthy neighboring cells by releasing reactive oxygen species as well as inflammatory mediators, including but not limited to, interleukin(IL)-6, IL-1, and IL-8 [1, 6, 7]. The accumulation of senescent cells in vessels leads to chronic inflammation and oxidative stress resulting in endothelial dysfunction and arterial stiffening [1].

Sirtuins are mammalian homologs of the yeast Sir2 protein that is known to mediate chromatin silencing, DNA repair, and longevity [8]. There are seven known sirtuins in mammals. Endothelial cells express all sirtuin types [9]. Among the seven known sirtuins, sirtuin 1 (SIRT1) is the most extensively analyzed sirtuin in endothelial cells and is known to be an important regulator of vascular endothelial aging by preventing DNA damage, cell cycle arrest, and oxidative stress [10]. SIRT1 exerts its effects by regulating endothelial nitric oxide synthase (eNOS), liver kinase B1, p53, angiotensin II type 1 receptor, and Forkhead box O (FOXO) 1 [11, 12]. There has been some research on the role of SIRT6 in the vasculature, but the research has not been as extensive as it has been for SIRT1. SIRT6, a nuclear and cytosolic protein, has NAD^+^-dependent deacetylase [13] and/or mono-ADP-ribosyltransferase [14] activities in many cell types. Loss of SIRT6 is known to associate with vascular endothelial dysfunction, senescence, and inflammation [15, 16], whereas induction of SIRT6 expression by exogenous vector-driven SIRT6, antioxidant, and anti-inflammatory signals prevents or ameliorates endothelial dysfunction, senescence, and inflammation [16, 17]. The protective effects of SIRT6 on endothelial function are known to occur by inhibition of ICAM-1, PAI-1, p21Cip1/Waf1, and NF-κB signaling and sustaining high eNOS levels [11]. However, the more descriptive mechanism underlying the protective role of SIRT6 in endothelial cells is yet to be explored. In the present study, we investigated the role of SIRT6 in relation to the senescent phenotypes of endothelial cells and the pathway responsible for the SIRT6-associated senescence in endothelial cells.

## RESULTS

### SIRT6 expression is downregulated in senescent endothelial cells

**Figure 1:**
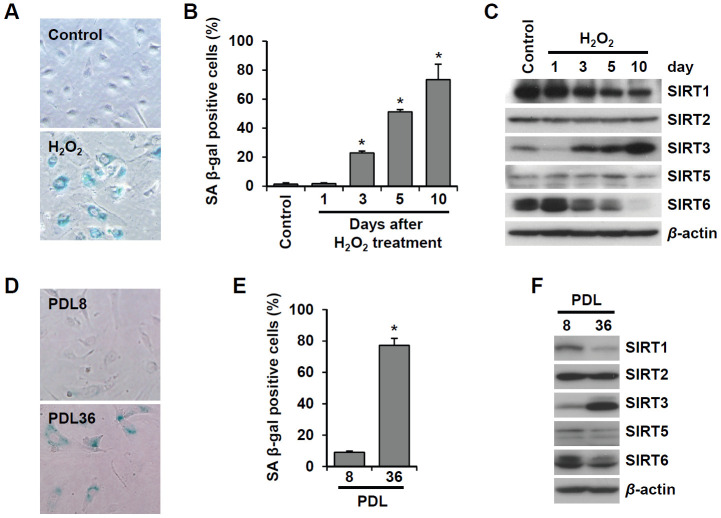
**SIRT6 expression is inhibited in endothelial cells during oxidative stress-induced or replicative senescence.** (**A**) Representative image of SA β-gal-positive HUVECs 10 d after the addition of H_2_O_2_ (200 μM). (**B**) The percentage of SA β-gal-positive senescent HUVECs that were treated with 200 μM H_2_O_2_ for 1 h and then cultured for the indicated time to generate oxidative stress-induced senescence. The data represent the mean percentage ± SD (n = 3). *P < 0.01 vs. control. (**C**) Western blot images to analyze the expression of SIRT1, SIRT2, SIRT3, SIRT5, and SIRT6 in HUVECs at 1, 3, 5, or 10 d after addition of H_2_O_2_ (200 μM). (**D**) SA β-gal staining images for young (PDL8) and old (PDL36) cells. (**E**) The percentage of SA β-gal-positive HUVECs that were passaged to induce replicative senescence. The data are shown as the mean ± SD (n = 3). *P < 0.01 vs. young cells. (**F**) The expression of SIRTs in young and old HUVECs. An antibody recognizing β-actin was used as a loading control.

**Figure 2:**
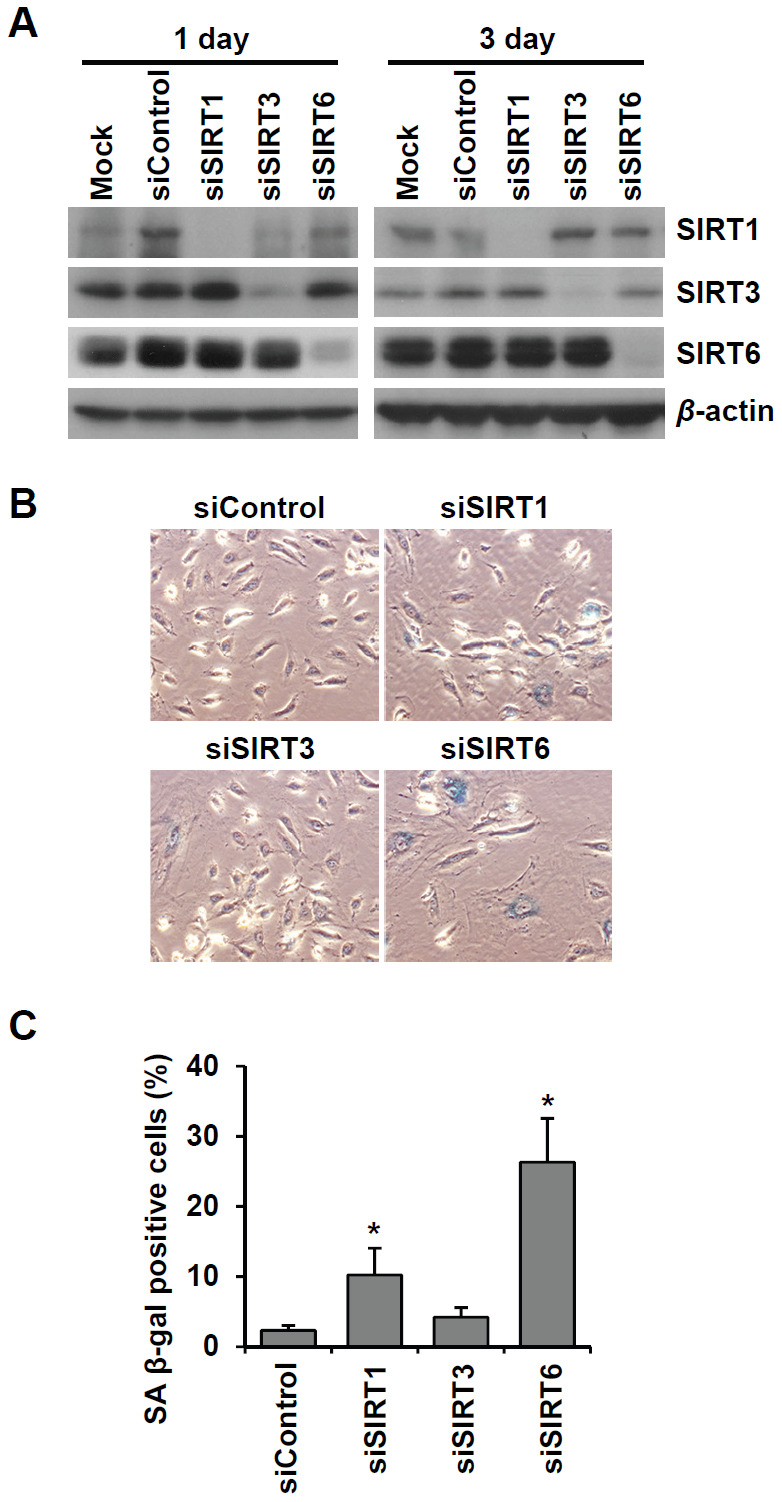
**Knockdown of SIRT6 expression induces endothelial cell senescence.** (**A**) Western blot analysis showing the knockdown expression of SIRT1, SIRT3, and SIRT6 in HUVECs treated with *SIRT1*, *SIRT3*, and *SIRT6* siRNAs, respectively. Total protein was extracted from cells 1 and 3 d after siRNA treatment. (**B**) The representative images obtained from SA β-gal-stained HUVECs. The cells transfected with the indicated siRNA (25 nM) were re-transfected with the siRNA 3 d after the first siRNA treatment. After 6 d from the first transfection, cells were stained for SA β-gal. (**C**) The percentage of SA β-gal-positive senescent cells at 6 d after siRNA transfection. The data are shown as the mean ± SD (n = 3). *P < 0.05 vs. control siRNA.

To examine the effect of cellular senescence on SIRT expression, we induced oxidative stress-induced senescence of human umbilical vein endothelial cells (HUVECs) by treatment with 200 μM H_2_O_2_ and validated the cellular senescence using senescence-associated β-galactosidase (SA β-gal) assay (Figure 1A). SA β-gal-positive endothelial cells gradually increased over time after H_2_O_2_ treatment, and 23% and 51% of cells were SA β-gal positive on day 3 and day 5, respectively (Figure 1B). Western blot analysis showed that SIRT1 and SIRT6 expression was significantly reduced on days 3, 5, and 10 following H_2_O_2_ treatment (Figure 1C). When approximately 73% of HUVECs were SA β-gal positive, SIRT6 expression was almost undetectable. Compared to SIRT1 and SIRT6, SIRT3 expression increased on days 3, 5, 10 after H_2_O_2_ treatment (Figure 1C). To confirm the expression pattern of SIRTs in senescent endothelial cells, we used another senescence model (i.e. replicative senescence achieved by repeated passage) to show that cell proliferation stopped and cells entered senescence. We compared SIRT expression in young cells (population doubling level 8, PDL8) to that in old cells (PDL36) using western blot analysis. Replicative senescence in HUVECs was confirmed using the SA β-gal assay (Figure 1D). The percentage of SA β-gal-positive HUVECs in PDL36 was 77% (Figure 1E). In keeping with the pattern of SIRT expression in the oxidative stress-induced senescence of endothelial cells, old endothelial cells showed a clear decrease in SIRT1 and SIRT6 expression and an increase in SIRT3 expression (Figure 1F). To study whether the downregulation of SIRT1, SIRT3, and SIRT6 expression affected endothelial cell senescence, HUVECs were treated with *SIRT1*, *SIRT3*, and *SIRT6* siRNA. *SIRT1*, *SIRT3*, and *SIRT6* knockdown with siRNA treatment was confirmed by western blot analysis (Figure 2A). SIRT1 and SIRT6 downregulation significantly increased the population of SA β-gal-positive cells 6 d after siRNA treatment, but *SIRT3* knockdown did not induce endothelial senescence (Figure 2B, 2C). The number of SA β-gal positive cells in *SIRT6* knockdown cells was 2.6-fold higher than that in *SIRT1* knockdown cells. We confirmed *SIRT6* knockdown-induced senescence using a different sequence of SIRT6 siRNA (siSIRT6*, Supplementary Figure 1A–1D). These data suggest that the downregulation of SIRT6 expression itself is enough to induce endothelial cell senescence.

### SIRT6 is involved in the maintenance of endothelial cell function

**Figure 3:**
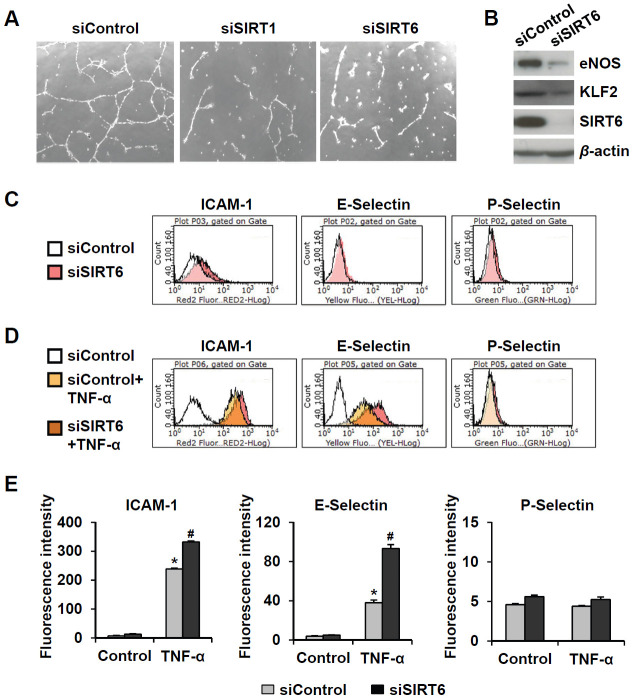
**Downregulated expression of SIRT6 induces endothelial cell dysfunction.** (**A**) Effect of *SIRT6* siRNA on *in vitro* tube formation in HUVECs. HUVECs transfected with 25 nM of the indicated siRNA were cultured on Matrigel to check *in vitro* angiogenesis activity of endothelial cells. The representative micrographs of tube formation in HUVECs. (**B**) Western blot analysis showing the effect of *SIRT6* siRNA on the expression of eNOS and KLF2. β-Actin was used as a loading control. (**C**, **D**) Representative flow cytometry plots showing the effect of *SIRT6* knockdown on cell surface expression of ICAM-1, E-selectin, and P-selectin. HUVECs transfected with 25 nM control or *SIRT6* siRNA were treated or not treated with TNF-α (50 ng/mL) for 4 h. Cells were stained with the fluorochrome-conjugated antibodies and analyzed by flow cytometry. (**E**) Graphs showing ICAM-1, E-selectin, and P-selectin expression levels in the cells. Data were obtained by analyzing the mean fluorescence intensity of each inflammatory molecule on HUVECs, which were differentially treated with control or *SIRT6* siRNA in the absence and presence of TNF-α. *P < 0.01 vs. control siRNA. #*P* < 0.01 vs. control siRNA with TNF-α.

Senescent endothelial cells have impaired angiogenic function and are susceptible to inflammatory responses. To evaluate the effect of *SIRT6* knockdown on capillary tube formation and inflammation in HUVECs, cells were transfected with 25 nM control, *SIRT1*, or *SIRT6* siRNA. When endothelial cells were cultured on Matrigel, the cells formed capillary-like tube network. *SIRT1* and *SIRT6* siRNA-transfected HUVECs on Matrigel showed reduced branch points and very short tubes (Figure 3A). Moreover, *SIRT6* knockdown inhibited eNOS and KLF2 expression (Figure 3B), which play essential roles in maintaining endothelial integrity [18, 19]. Depletion of SIRT6 resulted in an increase in the inflammatory responses of endothelial cells (Figure 3C–3E). *SIRT6* knockdown increased ICAM-1 expression but not E- and P-selectin expression. TNF-α-treated HUVECs highly expressed ICAM-1 and E-selectin. Interestingly, *SIRT6* siRNA treatment upregulated TNF-α-induced ICAM-1 and E-selectin expression compared to control siRNA treatment with TNF-α.

### Downregulation of SIRT6 expression is associated with vascular senescence in mice

**Figure 4:**
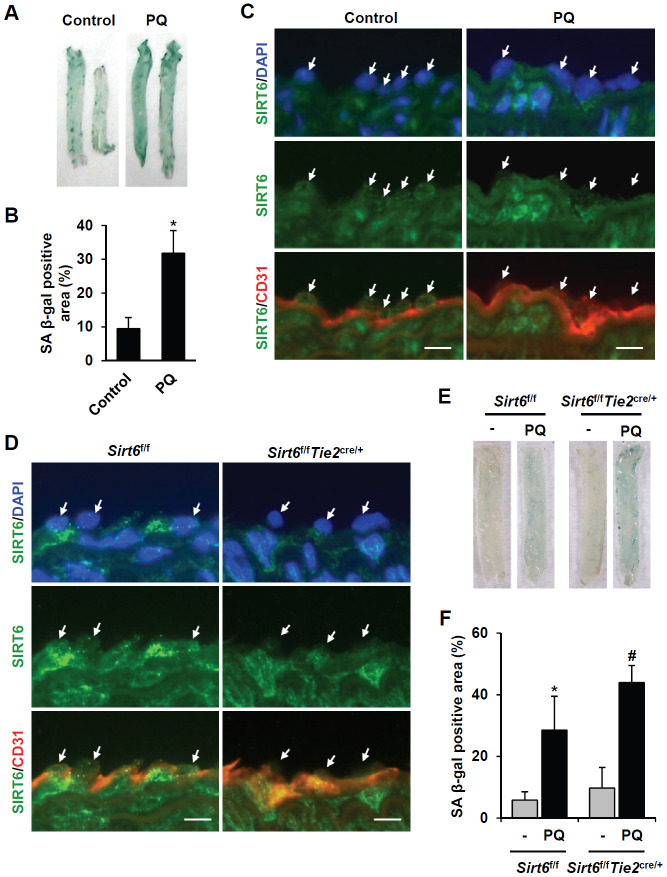
**SIRT6 expression is downregulated in mouse senescent aorta, and endothelial-specific *Sirt6* knockout in mouse deteriorates oxidative stress-induced senescence in the aorta.** (**A**) Images from SA β-gal staining of thoracic aorta from C57/BL6 mice injected with PBS or PQ. (**B**) Graph showing the relative SA β-gal-positive areas in PBS- and PQ-treated thoracic aortas. SA β-gal-positive areas were quantified using ImageJ. The experiment was repeated twice. Data represent the mean percentage ± SD (n = 4). *P < 0.05 vs. control treatment. (**C**) Double immunofluorescence staining showing SIRT6 and CD31 expression in control and PQ-treated thoracic aortas. The sections were co-stained with anti-SIRT6 and anti-CD31 antibodies. DAPI was used to stain nuclei. Arrows indicate nuclei of endothelial cells. Scale bars represent 10 μm. (**D**) Double immunofluorescence staining confirming *Sirt6* knockout in *Sirt6^f/f^Tie2^cre/+^* mouse thoracic aortas. The sections were co-stained with anti-SIRT6 and anti-CD31 antibodies. DAPI was used to stain nuclei. Arrows indicate nuclei of endothelial cells. Scale bars represent 10 μm. (**E**) Dissecting microscope images of thoracic aortas stained for SA β-gal. The thoracic aortas were obtained from *Sirt6^f/f^* and *Sirt6^f/f^Tie2^cre/+^* mice injected with PBS or PQ. (**F**) Relative SA β-gal-positive areas in the mouse thoracic aortas from *Sirt6^f/f^* and *Sirt6^f/f^Tie2^cre/+^* mice injected with PBS or PQ. The percentage of SA β-gal-positive areas was quantified using ImageJ. The experiment was repeated twice. Data are shown as the mean ± SD (n = 4). *P < 0.05 vs. control *Sirt6^f/f^*. #*P* < 0.05 vs. *Sirt6^f/f^* treated with PQ.

Next, we focused on the effect of vascular senescence on *in* *vivo* SIRT6 expression using a paraquat dichloride x-hydrate (PQ)-induced senescence mouse model [20]. Injection of PQ, a herbicide that induces oxidative stress, in mice resulted in senescence of the thoracic artery. Intraperitoneal treatment of PQ for 3 d significantly induced SA β-gal-positive cells in thoracic arteries (Figure 4A, 4B). Using immunofluorescence, we checked whether PQ-induced vascular senescence affected SIRT6 expression in endothelial cells. SIRT6 was mainly localized in the nucleus of vascular endothelial cells. PQ treatment decreased SIRT6 expression in endothelial cells, which were located on the luminal surface and stained with CD31, an endothelial cell marker (Figure 4C). Subsequently, to understand whether selective depletion of SIRT6 expression in aortic endothelial cells can stimulate vascular senescence, we generated conditional knockout mice in which SIRT6 expression is selectively ablated in endothelial cells by Cre-loxP recombination. *Sirt6^f/f^* mice possessing loxP sites flanking exon 2-3 of the *Sirt6* gene were crossed with *Tie2-Cre* mice expressing Cre recombinase under the control of a *Tie2* promotor, generating *Sirt6^f/f^Tie2^cre/+^* mice. The depletion of SIRT6 expression in thoracic aortic endothelial cells was confirmed by staining with an anti-SIRT6 antibody (Figure 4D). The viability, growth rate, and fertility of *Sirt6^f/f^Tie2^cre/+^* mice were the same as those of control mice. We examined senescence development in the isolated thoracic aortas from *Sirt6^f/f^Tie2^cre/+^* mice using SA β-gal staining. Compared to control *Sirt6^f/f^* mice, *Sirt6* ablation in aortic endothelial cells did not affect vascular senescence. However, in PQ-treated mice, there were larger SA β-gal-positive areas in the aortas of *Sirt6^f/f^Tie2^cre/+^* than in the aortas of control mice (Figure 4E, 4F). These data suggested that decreased SIRT6 expression is not sufficient to induce *in vivo* vascular senescence, but the downregulation of SIRT6 may exacerbate vascular senescence together with external and/or additional factors such as oxidative stress.

### Genes associated with cellular growth and proliferation as top-affected genes by *SIRT6* knockdown

**Figure 5:**
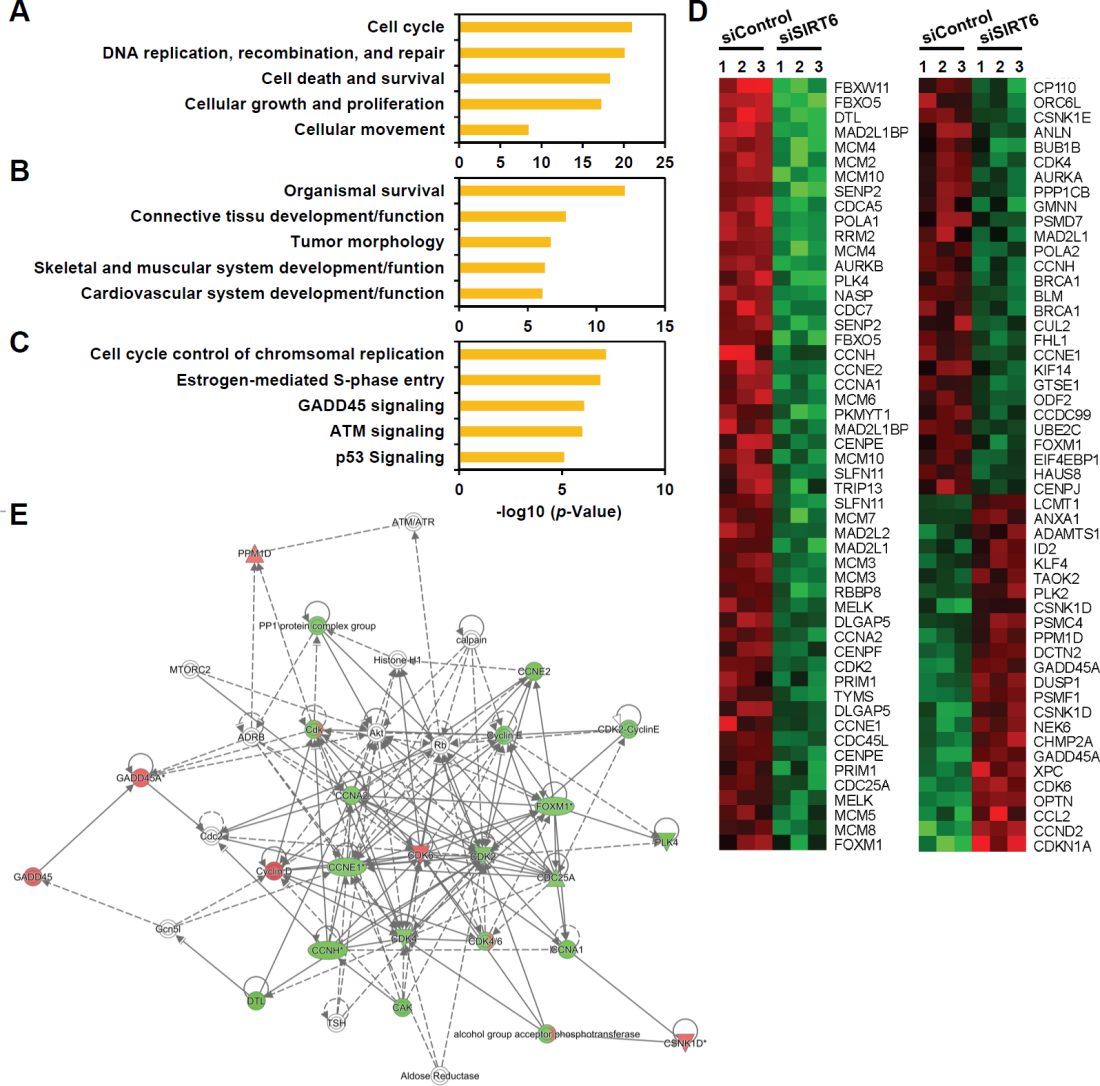
**Genes associated with cell proliferation are the genes most affected by *SIRT6* knockdown in endothelial cells.** HUVECs were transfected with 25 nM of control or *SIRT6* siRNA. After 3 d, total RNA was isolated, and the gene expression profile was assessed with the Illumina bead chip analysis. The identified genes that were differentially regulated by *SIRT6* knockdown were analyzed by the Ingenuity Pathway Analysis program to determine the *SIRT6* gene function in endothelial cells. (**A**, **B**) Top five groups of genes categorized by molecular and cellular functions (**A**) and physiological system development and function (**B**). (**C**) Top five canonical pathways affected by *SIRT6* knockdown. (**D**) Heat map showing the genes specifically involved in cell cycle transition regulation. Rows show individual genes, and columns show triplicate samples. Up- and downregulated genes are shown in red and green, respectively. (**E**) A gene functional association network for the genes involved in cell cycle transition regulation. The intensity of the node colors (red and green) indicates the degree of up- and downregulation.

To understand the mechanistic pathways linked to *SIRT6* depletion-induced endothelial cell senescence, we compared the gene transcription profiles of *SIRT6* siRNA-treated HUVECs with control cells using microarray analysis. We found 1,443 differentially expressing genes including 691 downregulated and 752 upregulated genes with more than 1.3-fold change (Supplementary Table 1). Ingenuity pathway analysis revealed that the top five molecular and cellular functions were cell cycle, DNA replication/recombination/repair, cell death/survival, cellular growth/proliferation, and cell movement (Figure 5A). Moreover, the top five groups of genes in terms of physiological system development and function were associated with organismal survival, connective tissue development and function, tumor morphology, skeletal and muscular system development and function, and cardiovascular system development and function (Figure 5B). Analysis of canonical pathways showed that the top five pathways regulated by SIRT6 were cell cycle control of chromosomal replication, estrogen-mediated S phase entry, GADD45 signaling, ATM signaling, and p53 signaling (Figure 5C). Up- or down-regulated genes involved in cell cycle transition regulation are presented as a heatmap and as a regulatory network (Figure 5D, 5E). We found that there was a significant increase in the expression of the cell cycle inhibitor CDKN1A (p21 Cip1) and a decrease in the expression of CDK2, CDK4, CCNA1, CCNA2, CCNE1, CCNE2, and FOXM1 in *SIRT6* knockdown HUVECs. Taken together, these data suggest that *SIRT6* knockdown-induced senescence occurred due to changes in the regulation of genes involved in cell cycle and that SIRT6 played a critical role in maintaining cell cycle progression in endothelial cells. The results from the microarray analysis also provided FOXM1 as a potential novel gene associated with SIRT6-induced cell cycle arrest in endothelial cells (Figure 5E).

### *SIRT6* knockdown induces cell growth arrest in endothelial cells

**Figure 6:**
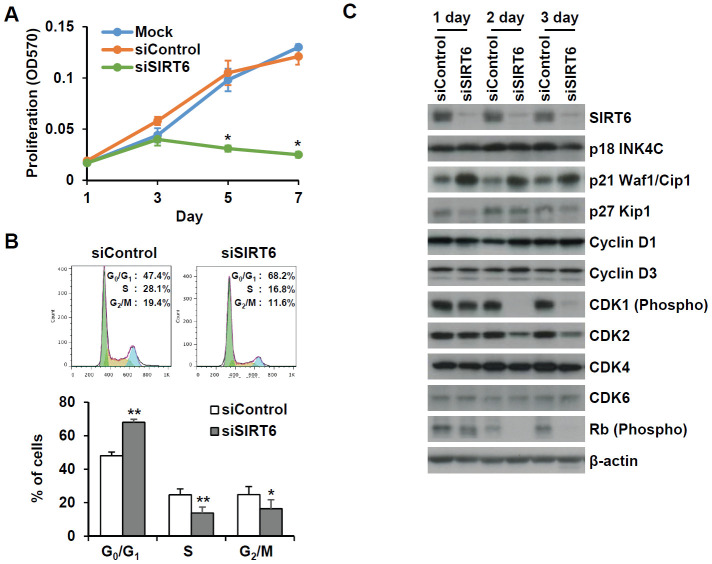
***SIRT6* knockdown significantly induces cell proliferation in endothelial cells.** (**A**) MTT assay showing the effect of *SIRT6* knockdown on endothelial cell proliferation. HUVECs were transfected with 25 nM control or *SIRT6* siRNA and incubated for the indicated number of days. *P < 0.01 vs. control siRNA. (**B**) Cell cycle analysis indicating that *SIRT6* siRNA induces cell cycle arrest in endothelial cells. HUVECs were transfected with 25 nM control or *SIRT6* siRNA. After 3 d, cells were stained with PI and analyzed using flow cytometry. Graphs show the mean percentage ± SD of cells in G_0_/G_1_, S, and G_2_/M phases. *P < 0.05 vs. control siRNA. **P < 0.01 vs. control siRNA. (**C**) Western blot analysis to determine the effect of *SIRT6* knockdown on the expression of cell cycle regulators. β-Actin was used as a loading control.

**Figure 7:**
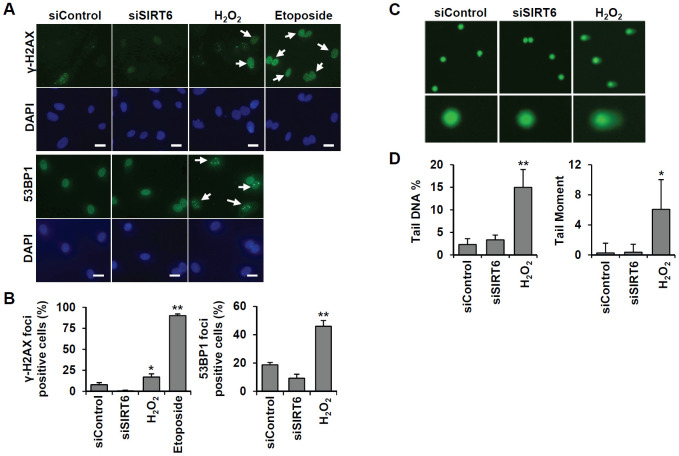
***SIRT6* knockdown does not directly induce DNA damage at the times when the cell cycle is inhibited.** (**A**) Representative images for the effect of *SIRT6* knockdown on the formation of DNA damage foci. HUVECs were transfected with 25 nM control or *SIRT6* siRNA. H_2_O_2_ (200 μm) and etoposide (10 μm) were used as positive controls to induce DNA damage. After 3 d, cells were stained with anti-γ-H2AX and anti-53BP1 antibodies. DAPI was used to stain nuclei. Scale bars represent 20 μm. (**B**) Quantification of DNA-damaged cells. Cells with more than ten γ-H2AX or five 53BP1 foci were scored. Data are expressed as the mean ± SD (n = 3). *P < 0.05 vs. control siRNA. **P < 0.001 vs. control siRNA. (**C**) Comet images of HUVECs treated with control or *SIRT6* siRNA. HUVECs were treated with 25 nM control or *SIRT6* siRNA. After 3 d, dissociated single cells were subjected to alkaline comet assay. Cells treated with H_2_O_2_ for 1 h were used as positive control. (**D**) Analysis of comet images. Percent DNA in the tail and tail moment of damaged cells were quantified using OpenComet. Data are expressed as the mean ± SD (n = 3). *P < 0.05 vs. control siRNA. **P < 0.01 vs. control siRNA.

To understand whether SIRT6 plays a regulatory role in endothelial cell division, growth of *SIRT6* siRNA-treated HUVECs was compared to that of control siRNA-treated cells via the MTT assay and live cell counting using trypan blue (Figure 6A and Supplementary Figure 1E). Treatment with *SIRT6* siRNA significantly inhibited HUVEC proliferation. Cell cycle analysis showed that the incubation of cells with *SIRT6* siRNA for 3 d resulted in cell cycle arrest (Figure 6B). We observed a significant increase in the number of cells in the G_0_/G_1_ phase among *SIRT6* knockdown cells, along with a decrease in the number of cells in S and G_2_/M phases, indicating that SIRT6 plays an important role in cell cycle progression in endothelial cells. Next, we investigated whether the depletion of SIRT6 expression affected the expression of cell cycle regulatory factors (Figure 6C). The expression of the cyclin-dependent inhibitor p21 was highly upregulated on day 1 after *SIRT6* siRNA transfection. In addition to the increase in p21 expression, CDK2, CDK4, and phosphorylated forms of Rb expression were significantly downregulated in cells transfected with *SIRT6* siRNA compared to cells transfected with control siRNA. We also saw that *SIRT6* knockdown inhibited CDK1 phosphorylation. These data suggested that SIRT6 expression is important for maintaining and controlling factors involved in cell cycle regulation in vascular endothelial cells. Together with the expression and activities of cell cycle regulators, cell cycle arrest can be affected by DNA damage. To determine whether *SIRT6* knockdown induced DNA damage, we quantified γH2AX and 53BP1 foci, sensitive markers of DNA damage, in HUVECs treated with *SIRT6* siRNA for 3 d (Figure 7A). Approximately 90% and 20% of cells were γH2AX foci positive among etoposide (a DNA topoisomerase II inhibitor)- and H_2_O_2_-treated cells, respectively (Figure 7B). However, *SIRT6* siRNA-treated cells did not show a significant increase in γH2AX foci-positive cells. In addition, the number of 53BP1 foci-positive cells was unchanged upon *SIRT6* knockdown, although H_2_O_2_ significantly stimulated 53BP1 foci formation (Figure 7A, 7B). To confirm that SIRT6 downregulation was not directly linked to DNA damage, we performed alkaline comet assay that is able to detect single and double-stranded DNA breaks in single cell level. Treatment of HUVECs with H_2_O_2_ for 1 h significantly induced DNA damages resulting in comet tail, migrating DNA fragments, from the nucleoid (Figure 7C, 7D). However, *SIRT6* knockdown did not induce DNA strand breaks. No differences in the tail DNA percent or tail moment were observed in control and *SIRT6* siRNA-treated cells 3 days after transfection (Figure 7C, 7D). We also assessed the level of DNA damage response proteins, phosphorylated p53. Phosphorylation of p53 at serine 15 can be induced by DNA damage sensing proteins, ataxia telangiectasia mutated kinase and ataxia telangiectasia and Rad3-related kinase [21]. *SIRT6* siRNA treatment did not stimulate the phosphorylation of p53 (Supplementary Figure 2). These data demonstrate that downregulated SIRT6 is closely associated with cell cycle arrest not attributable to prevalent DNA damage.

### *SIRT6* knockdown-induced senescence is associated with a decrease in FOXM1 expression, and FOXM1C induction ameliorates the senescence phenotype in endothelial cells

**Figure 8:**
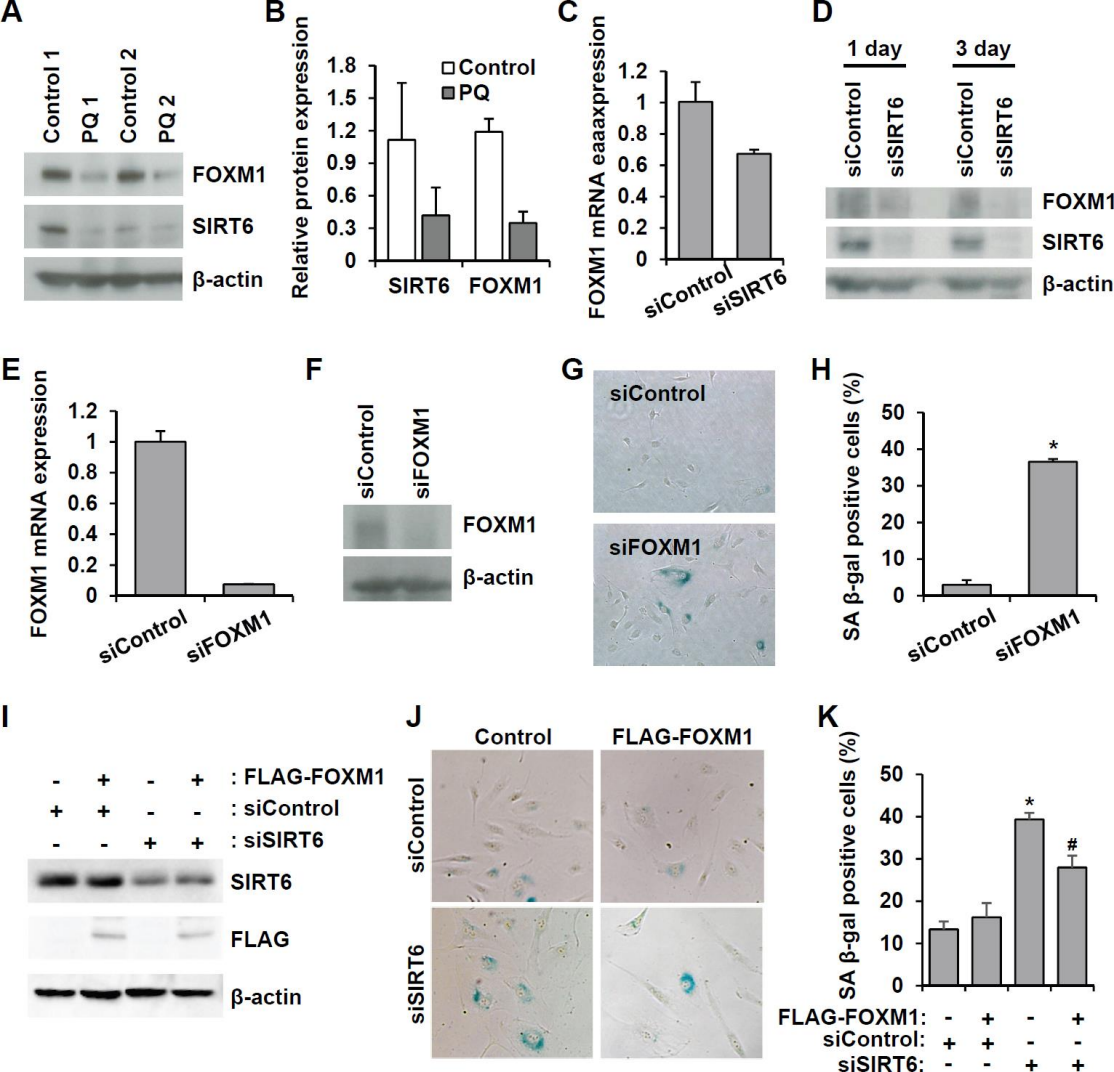
**Downregulated SIRT6 expression inhibits FOXM1 expression, which is closely related to endothelial cell senescence.** (**A**) Western blot analysis showing downregulation of FOXM1 and SIRT6 expression in thoracic aortas of mice treated with PBS or PQ. (**B**) Relative SIRT6 and FOXM1 protein expression in control and PQ-treated aortas. The protein expression was quantified using Bio-Rad Image Lab software. Relative expression was normalized to β-actin. (**C**) Real-time RT-PCR analysis indicating that *SIRT6* knockdown transcriptionally inhibited FOXM1 expression. (**D**) Western blot analysis presenting the effect of *SIRT6* knockdown on FOXM1 protein expression. (**E**) Real-time RT-PCR confirming the efficient knockdown of FOXM1 expression in endothelial cells by 25 nM *FOXM1* siRNA. (**F**) Western blot analysis to show the knockdown of FOXM1 protein in *FOXM1* siRNA-treated HUVECs. (**G**) Representative images of SA β-gal-positive senescent HUVECs that were transfected with control or *FOXM1* siRNA. (**H**) Quantification of data from (**G**). The mean percentage of SA β-gal-positive cells was calculated, and error bars indicate SD (n = 3). *P < 0.001 vs. control siRNA. (**I**) Western blot analysis showing overexpression of FOXM1 in control or *SIRT6* siRNA-treated endothelial cells. HUVECs were infected with 10 MOI of lentivirus vector encoding FLAG-tagged SIRT6. One day later, cells received the first transfection with 25 nM control or *SIRT6* siRNA. Next, cells were transfected with the same siRNA 3 d after the first transfection. Six days after the first transfection, cells were analyzed for SIRT6 and FLAG-tagged FOXM1 expression. β-Actin was used as a loading control. (**J**) Representative images showing that overexpression of FOXM1 inhibited *SIRT6* knockdown-induced endothelial cell senescence. Cells were stained for SA β-gal. (**K**) Quantification of data from (**J**). Data are expressed as the mean ± SD (n = 3). *P < 0.001 vs. control siRNA. #*P* < 0.01 vs. *SIRT6* siRNA.

FOXM1 is a master regulator essential for different phases of the cell cycle [22]. FOXM1 repression was observed during cellular aging, and recovery of its expression prevented senescence [23–25]. Microarray analysis revealed that FOXM1 was one of the factors affected by *SIRT6* knockdown. Additionally, western blot analysis using senescent aortas collected from PQ-treated mice also supported the idea that together with a decrease in SIRT6 expression, FOXM1 expression is repressed in senescent aortas (Figure 8A, 8B). To clarify the effect of *SIRT6* knockdown on FOXM1 expression, we checked the mRNA and protein expression of *FOXM1* in *SIRT6* knockdown-induced senescent HUVECs (Figure 8C, 8D and Supplementary Figure 1F). According to real-time RT-PCR and western blot analyses, decreased SIRT6 expression reduced FOXM1 expression, indicating that FOXM1 expression was closely linked to SIRT6 expression in HUVECs. When HUVECs were treated with *FOXM1* siRNA (Figure 8E, 8F), the number of SA β-gal-positive cells increased, suggesting that repression of FOXM1 expression itself can induce endothelial senescence (Figure 8G, 8H). Human *FOXM1* gene consists of ten exons, of which exons Va and VIIa are alternatively spliced [26, 27]. There are four FOXM1 isoforms, FOXM1A, FOXM1B, FOXM1C, and FOXM1D (Supplementary Figure 3A). To identify which isoforms are expressed in HUVECs, we performed RT-PCR using primer sets flanking exon Va or VIIa. Primers P1 and P2 predominantly amplified 250 bp of FOXM1A or FOXM1C forms containing Va exon. The most of PCR products amplified by primers P3 and P4 were 379 bp fragment that did not have VIIa exon (Supplementary Figure 3B). These data suggested that FOXM1C is the major isoform expressed in HUVECs. To investigate whether restoration of FOXM1C expression in *SIRT6* siRNA-treated HUVECs can ameliorate cellular senescence, we generated lentivirus encoding FLAG-tagged FOXM1C and infected HUVECs with this lentivirus. We confirmed *SIRT6* knockdown and the induction of FLAG-FOXM1C by western blotting (Figure 8I). SA β-gal staining showed that the rescued FOXM1C expression significantly blocked *SIRT6* knockdown-induced endothelial cell senescence (Figure 8J, 8K).

## DISCUSSION

Strategies to slow down the aging process at a systemic or tissue level have attracted a great deal of attention because there is evidence to suggest that slowed aging results in a prolonged lifespan by delaying and preventing disease onset for chronic conditions in adulthood and old age [28, 29]. Cardiovascular disease, a major cause of death, is an age-related disease. Therefore, it is important to understand the mechanisms underlying vascular aging. One of the most promising gene targets for slowing down vascular aging is SIRT1 [28]. Sirtuin-activating compounds, including resveratrol and other synthetic compounds, have been tested in clinical trials to assess their ability to prevent aging and age-related diseases [29]. Although other types of sirtuins are also thought to be involved in the aging process, few studies have explored the effect of other sirtuins on vascular aging. Herein, we show SIRT6 is significantly downregulated in senescent HUVECs and that siRNA-mediated knockdown of SIRT6 expression induces endothelial cell senescence. We also showed that inhibition of SIRT6 expression impairs *in vitro* angiogenesis and eNOS and KLF2 expressions that play an important role in the maintenance of endothelial cell function. Moreover, SIRT6 inhibition increases ICAM-1 expression and enhances TNF-α-induced ICAM-1 and E-selectin expression. All of the phenotypes that we observed in *SIRT6* siRNA-treated HUVECs are equivalent to senescent phenotypes (e.g. reduced capacity of new vessel formation, endothelial dysfunction, and high inflammatory responses) [30]. SIRT6 is known to physically interact with the NF-κB subunit RELA to negatively regulate NF-κB-dependent transcription via histone H3K9 deacetylation [31]. Therefore, SIRT6 downregulation can lead to inappropriate NF-κB activation, resulting in cell senescence and inflammation. We also found that senescence also resulted in an increase in SIRT3 expression. SIRT3 can deacetylate FOXO3 to upregulate a set of genes that are essential for mitochondrial homeostasis. Thus, SIRT3-activated signaling pathways preserve mitochondrial reserve capacity by the clearance of defective mitochondria and the maintenance of mitochondrial mass and ATP production [32]. In the present study, increased SIRT3 expression during senescence may be the result of efforts made by endothelial cells to protect the progressive accumulation of defective mitochondria, a common feature of aged cells.

A previous publication using an ApoE^-/-^ atherosclerotic animal model showed that *SIRT6* mRNA and protein levels were downregulated in atherosclerotic aortas and that SIRT6 has a protective role against the development of atherosclerosis. Our *in vivo* vascular aging model also showed that SIRT6 expression is significantly lower in the endothelial cells of senescent mouse aorta, which is consistent with our HUVEC data. In endothelial cell-specific SIRT6 knockout mice (*Sirt6^f/f^Tie2^cre/+^*), there were no differences between the control and SIRT6-deficient mouse aortas. Under the pathological condition of oxidative stress, endothelial cell-specific SIRT6 deficiency exacerbated PQ-induced senescence in the aorta. Our data suggest that there may be a complementary mechanism to counteract the effect of SIRT6 deficiency *in vivo* and that SIRT6 depletion under conditions of stress makes vessels more vulnerable to senescence. Similar to our findings, a study using endothelial-specific *Sirt6* knockout mice reported that SIRT6 deficiency did not produce statistically significant effects on blood pressure, but *Sirt6* knockout mice treated with deoxycorticosterone acetate/salt exhibited a more significant increase in blood pressure than that in control mice [33].

Cellular senescence involves a programed cell cycle arrest. Our microarray analysis supports the hypothesis that SIRT6 plays an important role in cell cycle regulation. The top signaling pathway associated with the SIRT6 function was cell cycle control. *SIRT6* siRNA treatment negatively affected endothelial cell proliferation, and *SIRT6* siRNA-treated cells were arrested in the G_0_/G_1_ phase. Depletion of SIRT6 inhibited CDK2, CDK4, phosphorylated CDK1, and phosphorylated Rb expression while it also increased p21 expression. The cell cycle arrest 3 days after *SIRT6* siRNA transfection does not appear to be due to DNA damages. SIRT6 depletion itself did not induce single and double-strand DNA damage. However, we cannot completely rule out the accumulation of DNA damage during *SIRT6* knockdown-induced senescence, which in turn can accelerate senescence. There are evidences supporting that SIRT6 is responsible for more efficient DNA damage repair [34–36]. In cells under oxidative stress, SIRT6 was recruited to the sites of DNA double-strand breaks and promoted DNA repair by physical association and activation of PARP1 [34]. The study using 18 rodent species with diverse lifespans showed that deacetylase and mono-ADP ribosylase activities of SIRT6 were much higher in longer-lived species than in shorter-lived ones. The enzymatic activities directly contributed to lifespan extension and correlated positively with efficiency of DNA repair [35]. Rezazadeh et al. also demonstrated that SIRT6 promoted repair of stress-induced DNA damage by transiently repressing transcription [36]. Therefore, instead of direct induction of DNA damage, cell cycle is sensitively controlled by SIRT6 protein levels. Inhibition of SIRT6 expression induces endothelial cell senescence via dysregulated expression of genes involved in cell cycle regulation. It would seem that exposing *SIRT6* knockdown cells to additional stresses such as oxidative stress and cell-culture stress could accelerate senescence due to accumulation of DNA damage caused by a defect in DNA repair.

Using microarray analysis, we have shown that FOXM1 is responsible for *SIRT6* deficiency-induced endothelial cell senescence. FOXM1 expression was significantly decreased in *SIRT6* siRNA-induced senescent HUVECs as well as in the senescent aortas of mice. FOXM1 is a transcription factor of the forkhead family and *FOXM1* gene consists of 10 exons. To date, four isoforms, FOXM1A, FOXM1B, FOXM1C, and FOXM1D, were identified [26, 27]. Among them, FOXM1B, FOXM1C, and FOXM1D are transcriptionally active, and FOXM1A is inactive. FOXM1D plays a role in actin cytoskeleton regulation through ROCK2 interaction [27]. FOXM1B is predominantly overexpressed in tumor and FOXM1C is ubiquitously expressed in various cell types [26]. FOXM1B and FOXM1C is a key cell cycle regulator of both the G_1_/S phase transition and mitosis progression. FOXM1 expression levels correlate with the state of cell proliferation. FOXM1 is highly expressed in proliferating cells [26]. FOXM1 deficiency is associated with decreased cell proliferation as it inhibits many cell cycle-regulatory genes that are known to promote cell cycle progression through S, G_2_, and M phases [26].

Our RT-PCR data indicated that FOXM1C is predominant isoform expressed in HUVECs. Consistent with our results, Lam et al. also reported that FOXM1C is the isoform highly expressed in HUVECs [37]. Recovery of FOXM1C expression in HUVECs ameliorated *SIRT6* siRNA-induced endothelial cell senescence. Similar to our findings, a study using aged fibroblasts also demonstrated repression of FOXM1 in aged cells and the protective effect of FOXM1 induction on cellular aging phenotypes [23]. Compared to young mice, old mice have a defect in hepatocyte proliferation following partial hepatectomy [38]. Increased FOXM1 expression in aged FOXM1 transgenic mice restored DNA replication and mitosis during liver regeneration, which was associated with the expression of genes that stimulate S phase and M phase progression [38]. *FOXM1* deficiency is known to induce pleiotropic cell cycle defects such as impairments of mitotic entry, chromosome segregation, and mitotic exit [26]. The known targets of FOXM1 are G_2_/M phase-promoting genes such as cyclin B1, Plk-1, Cdc25B phosphatase, and Aurora kinases. Additionally, CDK inhibitors p21 Cip1 and p27 Kip1 are elevated in FOXM1-deficient cells because of the downregulation of Skp2 and Cks1, which are important for the proteosomal degradation of p21 Cip1 and p27 Kip1 proteins [39]. Our discovery that *SIRT6* knockdown results in FOXM1 downregulation may be essential for elucidating the growth arrest phenotypes of senescent endothelial cells in the future.

## CONCLUSIONS

In conclusion, we present a *in vitro* and *in vivo* data showing that a decrease in SIRT6 expression is an important consequence of endothelial cell aging as well as a cause of endothelial senescence. Our findings suggest that the *SIRT6* gene function is closely associated with cell cycle regulation and that the SIRT6-regulated FOXM1 pathway is a novel pathway responsible for endothelial cell senescence. The mechanism by which SIRT6 regulates FOXM1 expression is unclear and should be further explored to aid our understanding of the protective role of SIRT6 in vascular aging. Moreover, our findings also highlight FOXM1 as a potential molecular target to prevent and treat cardiovascular diseases.

## MATERIALS AND METHODS

### HUVEC culture

HUVECs were purchased from Lonza (Walkersville, MD, USA) and maintained on 2% (w/v) gelatin-coated culture dish with Endothelial Growth Media-2 (EGM-2) BulletKit^TM^ medium (Lonza) in a 37° C humidified incubator with 5% CO_2_. HUVECs were sub-cultured before they reached 80% confluence to avoid irreversible contact-inhibition. HUVECs under eight population doublings (PDs) were used for the current experiments, with the exception of the induction of replicative senescence. The number of PDs was calculated using the following equation:

PD=log2 (CH/CS), (1)

where *C_H_* is the number of viable cells at harvest and *C_S_* is the number of cells seeded as described previously [40]. PD levels were obtained from the sum of all previous PDs. To induce replicative senescence, HUVECs were continuously passaged to reach the indicated PD levels. Premature oxidative stress-induced senescence of HUVECs was induced by H_2_O_2_ treatment. HUVECs under PDL 8 were treated with 200 μM for 1 h. Then, cells were washed with EGM-2 and further incubated in EGM-2 for the indicated time. To knockdown *SIRT1*, *SIRT3*, *SIRT6,* and *FOXM1* expression, HUVECs under PDL 8 were transfected with 25 nM of *SIRT1*, *SIRT3*, *SIRT6,* and *FOXM1* siRNAs, respectively, using Lipofectamine RNAiMax (Invitrogen, Carlsbad, CA, USA). Three days after the first siRNA transfection, cells were re-transfected with siRNA. *SIRT1* (GAAGTTGACCTCCTCATTG), *SIRT3* (GTCCATATCTTTTTCTGTG), *SIRT6* (GGAACATGTTTGTGGAAGA), and SIRT6* (CTGGTCTCCAGCTTAAACA) siRNAs were obtained from Bioneer (Daejeon, Korea), and ON-TARGETplus *FOXM1* siRNA (2305) was purchased from GE Healthcare Dharmacon (Lafayette, CO, USA).

### Mice

*Sirt6^flox/flox^* (*Sirt6^f/f^, Sirt6^tm1.1Cxd^*/J) and *Tie2-Cre* (*Tie2^cre/+^*, B6.Cg-Tg(*Tek-cre*)1Ywa/J) mice were purchased from the Jackson Laboratory (Sacramento, CA, USA). *Sirt6^f/f^* mice were backcrossed with C57BL/6 mice to produce congenic strains. Then, *Sirt6^f/f^* mice were crossed with *Tie2-Cre* mice expressing Cre recombinase under the control of a *Tie2* promotor to generate mice (*Sirt6^f/f^/Tie2^cre/+^*) deficient of endothelial cell SIRT6. Mice were genotyped by PCR according to the provider’s instruction. *Sirt6^f/f^* mice from the same litters were used as controls against *Sirt6^f/f^/Tie2^cre/+^* mice. C57BL/6 mice were obtained from Orient Bio Company (Seongnam, Korea). To induce senescence of aorta, male mice (8–9 weeks old) were intraperitoneally injected with 25 mg/kg PQ (Sigma-Aldrich, St. Louis, MO, USA). After 3 d, mice were anesthetized and sacrificed. After systemic perfusion with PBS, the thoracic aorta was excised and fixed with formalin or fixation buffer from the Senescence Cells Histochemical Staining Kit (Sigma-Aldrich). Animal care and experimental procedures were performed following approval from the Institutional Animal Care and Use Committee of CHA University (Approval No. IACUC160065).

### Lentiviral vector production and infection

To deliver the *FOXM1* gene into HUVECs, we generated a lentiviral vector encoding *FOXM1* tagged with FLAG. Full length *FOXM1* coding sequences were amplified by PCR from pCl-CMV-YFPc-FOXM1-puro/pCl-CMV-FOXM1-YFPc-puro in a retroviral array library [41] and then cloned into a pENTR/D-TOPO vector (Invitrogen). Using Gateway recombination (Invitrogen), the *FOXM1* coding sequences were transferred into destination lentiviral vector, pHAGE-CMV-N-FLAG-HA-puro, to obtain an N-terminally FLAG- and HA-tagged *FOXM1* (pHAGE-CMV-N-FLAG-HA-FOXM1-puro) expressing vector. Lentiviral vector particles were produced using the Lenti-X HTX packaging system (Clontech Laboratories, Mountain View, CA, USA) according to the manufacturer’s instruction. Briefly, Lenti-X 293T cells were plated in a 100 mm dish at a density of 5×10^6^ cells and transfected with Lenti-X HTX packaging mix and pHAGE-CMV-N-FLAG-HA-FOXM1-puro using Xfect polymer. After 2 d, lentiviral supernatants were harvested and passed through a 0.45 μm filter to remove cell debris. After concentrating the lentivirus supernatants with the Lenti-X concentrator, the titer of virus particles was determined by Lenti-X GoStix (Clontech Laboratories). HUVECs were seeded in 60 mm dishes at 1×10^5^ cells. One day later, cells were fed with EGM-2 containing 5 μg/mL of polybrene and treated with 10 multiplicity of infection (MOI) of lentivirus particles.

### SA β-gal staining

SA β-gal activity was measured using Senescence Cells Histochemical Staining Kit (Sigma-Aldrich) according to the manufacturer’s instructions. Briefly, the fixed HUVECs and thoracic aortas were incubated with staining-mixture containing X-gal at 37° C without CO_2_ until cells were stained blue. After washing with PBS, cells were covered with a 70% (v/v) glycerol solution. An image of positive SA β-gal cells and aortas was taken under a microscope, and cells were counted. The SA β-gal-positive areas in thoracic aortas were quantified using ImageJ (version 1.52a, National Institutes of Health, USA).

### Western blot analysis

Total protein was isolated from cells using cold radio immunoprecipitation assay buffer containing a protease inhibitor cocktail (Roche Applied Science, Penzberg, Bavaria, Germany). A total of 50 μg of protein was subjected to SDS-PAGE, and the blots were transferred onto a polyvinylidene difluoride membrane. After blocking, the membranes were incubated with anti-SIRT1 (Millipore, Temecula, CA, USA), anti-SIRT2 (Cell Signaling Technology, Danvers, MA, USA), anti-SIRT3 (Cell Signaling Technology), anti-SIRT5 (Millipore), anti-SIRT6 (Cell Signaling Technology), anti-eNOS (Cell Signaling Technology), anti-KLF2 (Santa Cruz Biotechnology, Santa Cruz, CA, USA), anti-FOXM1 (Cell Signaling Technology), anti-β-actin (Santa Cruz Biotechnology), and anti-FLAG (Sigma-Aldrich) antibodies. Antibodies against cell cycle regulators and checkpoint molecules, including cyclin D1, cyclin D3, p18 INK4C, p21 Waf1/Cip1, p27 Kip1, CDK2, CDK6, phospho-RB, and phospho-p53 (Ser15), were purchased from Cell Signaling Technology. Immune-reactive protein bands were visualized by chemiluminescence using ECL reagents (GE Healthcare, Fairfield, CT, USA). Protein expression was imaged in a ChemiDoc XRS system (Bio-Rad Laboratories, Hercules, CA, USA).

### Real-time reverse transcription-polymerase chain reaction (RT-PCR) analysis

At the indicated time after treatment with control, *SIRT6*, or *FOXM1* siRNA, total RNAs were isolated using the RNeasy RNA isolation kit (Qiagen, Hilden, Germany) and reverse-transcribed using SuperScript® III First-Strand Synthesis System (Thermo Fisher Scientific, Waltham, MA, USA). Real-time RT-PCR was performed using the SYBR green reagent (Roche Applied Science) and an iCycler (Bio-Rad Laboratories). The primers used here were as follows: *FOXM1* (5´-AAACCTGCAGCTAGGGATGT-3´ and 5´-AGCCCAGTCCATCAGAACTC-3´) and β-actin (5´-GGACTTCGAGCAAGAGATGG-3´ and 5´-AGCACTGTGTTGGCGTACAG-3´). PCR reactions were carried out in triplicate. The relative expression of *FOXM1* mRNA was normalized to the β-actin mRNA. To analyze which spliced isoforms of *FOXM1* gene were expressed in HUVECs, we performed RT-PCR. The used primers were as follows: P1 (5´-CGTGGATTGAGGACCACTTT-3´), P2 (5´-GGATTCGGTCGTTTCTGCTG-3´), P3 (5´-GGGCGCACGGCGGAAGATGAA-3´), and P4 (5´-CCACTCTTCCAAGGGAGGGCTC-3´). The PCR products were run on a 2% agarose gel.

### Immunostaining

The fixed aorta was embedded in paraffin, sectioned at 5 μm thickness, and placed on Superfrost Plus Stain slides (Thermo Fisher Scientific). After deparaffinizing the sections, antigen retrieval of tissue sections was performed using an Epitope Retrieval Solution System (IHCworld, Woodstock, MD, USA) according to the manufacturer’s instruction. The slides were incubated with a blocking buffer containing PBS with 4 % (w/v) BSA and 5% goat serum for 4 h at room temperature. Subsequently, the slides were incubated with primary antibodies such as anti-SIRT6 (Novus Biological, Littleton, CO, USA) and anti-CD31 (Santa Cruz Biotechnology) overnight at 4° C followed by secondary antibodies for 1 h at room temperature. Avidin-biotin-horseradish peroxidase complex (Vector Laboratories Ltd., Peterborough, Cambridgeshire, UK) and Alexa Fluor 488 or 546 conjugated antibodies (Thermo Fisher Scientific) were used as secondary antibodies. The peroxidase signals were detected using DAB solution (Vector Laboratories Ltd.), and the slides were counterstained with hematoxylin, dehydrated, and mounted in permount mounting medium (Thermo Fisher Scientific). Slides with fluorochrome antibodies were mounted with ProLong antifade reagent (Invitrogen) containing 4’,6-diamidino-2-phenylindole (DAPI). To stain HUVECs for γ-H2AX and 53BP1 foci, siRNA-treated HUVECs were cultured on gelatin-coated Lab-Tek chamber slides (Sigma-Aldrich). For the positive controls, HUVECs were treated with H_2_O_2_ (200 μM) and etoposide (10 μM) for 1 h. Cells were then washed, and fresh medium was supplied. Following incubation for 3 d, cells were fixed with 3% (w/v) paraformaldehyde/2% (w/v) sucrose in PBS for 10 min at room temperature. Cells were then permeabilized with permeabilization buffer containing 0.5% (v/v) Triton X-100, 20 mM HEPES-KOH (pH 7.9), 50 mM NaCl, 3 mM MgCl2, and 300 mM sucrose, followed by incubation with anti-53BP1 (BD Biosciences, San Jose, CA, USA) or anti-γ-H2AX (Millipore) antibodies. Next, 53BP1 and γ-H2AX proteins were visualized by treatment with an Alexa Fluor 488 conjugated anti-mouse antibody (Invitrogen).

### Flow cytometry analysis

Antibodies used for flow cytometry were purchased from BD Pharmingen (San Jose, CA, USA). Control or SIRT6 siRNAs were transfected into HUVECs at a concentration of 25 nM. After 3 d, the cells were treated with 50 ng/mL of rhTNF-α (R&D Systems, Minneapolis, MN, USA) for 4 h and prepared as a single cell suspension. Cells were then incubated with anti-E-selectin-phycoerythrin (PE), anti-ICAM-1-allophycocyanin (APC), and anti-P-selectin-fluorescein isothiocyanate (FITC). Immuno-stained cells were analyzed with a GUAVA Flow Cytometer (Millipore).

### Tube formation assay

Twelve-well plates containing 400 μL of polymerized growth factor-reduced Matrigel (Corning, Bedford, MA, USA) were used to validate tube formation of endothelial cells. HUVECs in EGM-2 medium were plated into Matrigel at a density of 1×10^5^ cells/well and incubated for 20 h at 37° C. Cultures were imaged (40× magnification) after control cells formed tubes.

### Proliferation assay

HUVEC proliferation was measured using 3-(4,5-dimethylthiazol-2-yl)-2,5-diphenyltetrazolium bromide (MTT, Sigma-Aldrich). HUVECs were seeded into 96-well plates at a density of 1×10^3^ cells/well. After 1 d, 25 nM of each siRNA was transfected into HUVECs and cultured in EGM-2 for the indicated time. HUVEC growth was measured by adding MTT solution (5 mg/mL) to each culture being assayed to equal one tenth of the culture volume, followed by incubation at 37° C for 4 h. After removing the medium, the converted formazan crystals were solubilized with 0.04 M HCl in isopropanol and quantified by optical density at a wavelength of 570 nm. The background signal was acquired at 690 nm and subtracted from the culture measurements.

### Microarray analysis

Total RNA was isolated from the HUVECs treated with control or *SIRT6* siRNA for 3 d using the RNeasy RNA isolation kit (Qiagen). Five-hundred nanograms of total RNA was used for labeling and hybridization, in accordance with the manufacturer’s protocols (#AMIL1791, Ambion, Inc.). Labeled RNA was hybridized with bead chips, which were then washed and scanned with an Illumina Bead Array Reader (Illumina, Inc. Sam Diego, CA, USA). The microarray data were normalized using the quantile normalization method in the Linear Models for Microarray Data (LIMMA) package in the R language environment. The expression of each gene was transformed into a log_2_ base before further analysis. The Ingenuity Pathway Analysis program (Redwood City, CA, USA) was used to identify specific gene subsets sharing functional annotation linking and to analyze specific biological and functional pathways.

### Cell cycle analysis

Cell cycle analysis was performed using GUAVA Cell Cycle Reagent (GUAVA Technologies, Hayward, CA, USA) according to the manufacturer’s instructions. Briefly, HUVECs transfected with 25 nM of each siRNA were incubated in EGM-2 at 37° C for 3 d. Cells were harvested with trypsin-EDTA treatment and dispersed as single cells. Cells were fixed with ice-cold 70% (v/v) ethanol and then, incubated in GUAVA Cell Cycle Reagent for 30 min at room temperature. Cell cycle phases were analyzed using the GUAVA Flow Cytometer and its associated Modfit software.

### Comet assay

To evaluate DNA damage in a cell, we performed an alkaline comet assay using CometAssay kit (Trevigen, Gaithersburg, MD, USA) according to the manufacturer’s instructions. Briefly, HUVECs transfected with 25 nM of control or *SIRT6* siRNA were incubated in EGM-2 for 3 d. Cells treated with 200 μM H_2_O_2_ for 1 h were used as positive control. Cells were gently detached by trypsin-EDTA treatment and suspended at 1 × 10^5^ cells/ml in ice cold PBS. Fifty microliter of cells combined with molten LMAgarose at a ratio of 1:10 (v/v) were dropped and spread onto CometSlide. After gelling LMAgarose, slides were treated with lysis solution for 1 h at 4° C and alkaline unwinding solution for 20 min at room temperature. The slides were placed in electrophoresis tray containing alkaline electrophoresis solution and subjected to electrophoresis at 21 volts for 40 min. After staining cells with SYBR green (Invitrogen), cell images were obtained at 200 × magnification using EVOS M5000 imaging system (Thermo Fisher Scientific). Percent DNA in the tail and tail moment from at least 50 randomly selected cells per sample were analyzed using OpenComet v1.3.1 software [42].

### Statistical analysis

The data are presented as the mean ± standard deviation (SD). Statistical significance was evaluated using a Student’s t-test and P-values < 0.05 were considered to be statistically significant. Two-way ANOVA was used for two variables.

## Supplementary Material

Supplementary Figures

Supplementary Table 1
