# Supplementary material for: Sirtuin 6 deficiency induces endothelial cell senescence via downregulation of forkhead box M1 expression
Source: Aging (Albany NY). 2020 Nov 10;12(21):20946–67. doi: 10.18632/aging.202176 (PMC7695388; doi:10.18632/aging.202176)
Supplement: Supplementary Figures [file aging-12-202176-s001..pdf]

SUPPLEMENTARY FIGURES

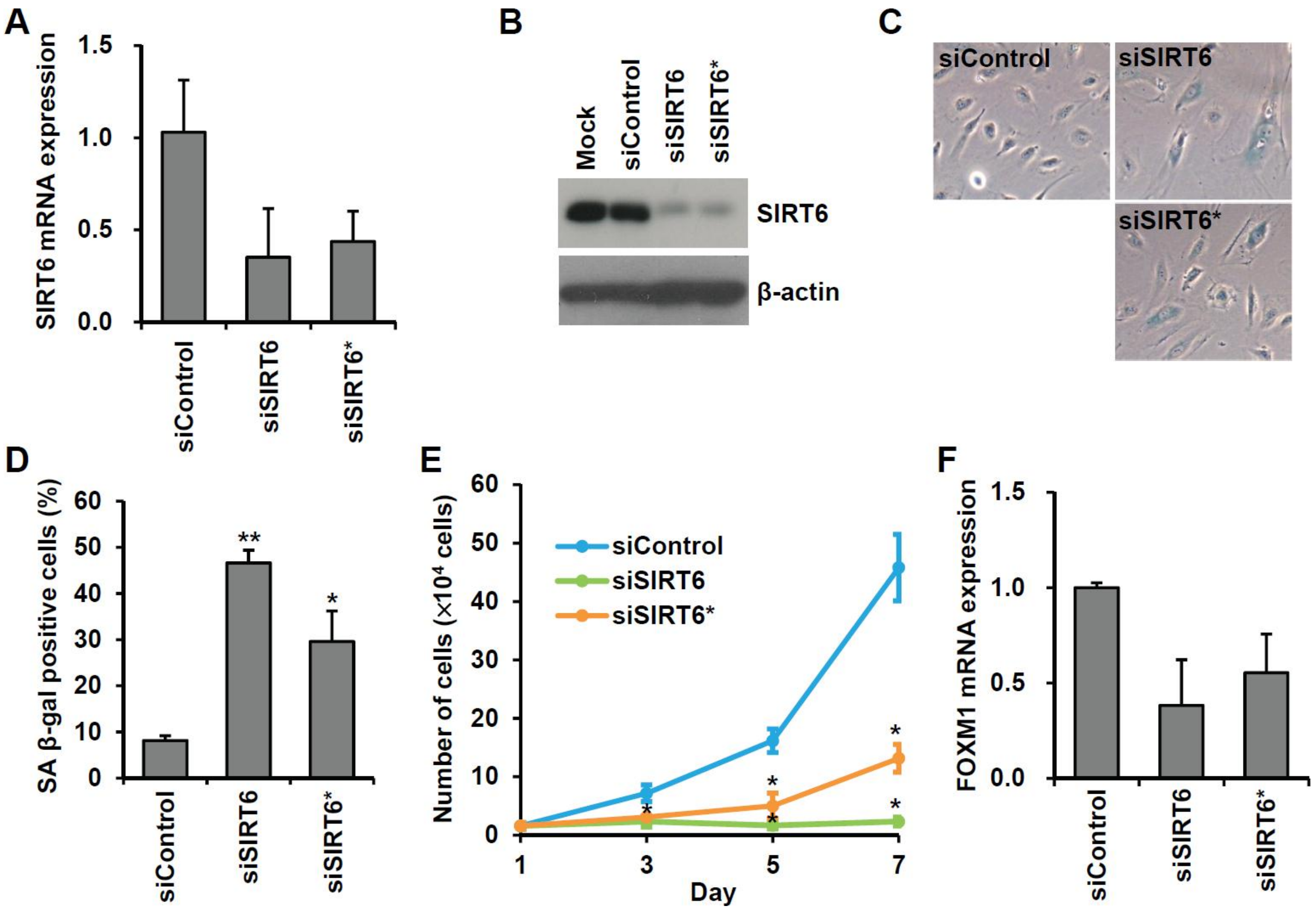

Supplementary Figure 1. Effects of *SIRT6* knockdown on senescence, proliferation, and FOXM1 expression of HUVECs. (**A**) Real time RT-PCR analysis to examine whether *SIRT6* siRNAs, siSIRT6 and siSIRT6*, could efficiently inhibit *SIRT6* expression. Total RNA was isolated from cells 3 d after siRNA (25 nM) transfection. (**B**) Western blot analysis showing that both siSIRT6 and siSIRT6* reduced SIRT6 protein expression. (**C**) The representative images obtained from SA β-gal-stained HUVECs. The cells transfected with *SIRT6* siRNA (25 nM) were re-transfected with the siRNA 3 d after the first siRNA treatment. After 6 d from the first transfection, cells were stained for SA β-gal. (**D**) The percentage of SA β-gal-positive senescent HUVECs. The data are shown as the mean ± SD (n = 3). *$P < 0.05$ or **$P < 0.01$ vs. control siRNA (**E**) Number of living HUVECs at the indicated time after 25 nM siRNA transfection. Trypsin-EDTA treated HUVECs were stained with trypan blue (0.4%, 1:1 dilution), and the number of living cells was measured using hemocytometer. The data are shown as the mean ± SD (n = 3). *$P < 0.01$ vs. control siRNA (**F**) Real time RT-PCR analysis indicating that *SIRT6* knockdown inhibited *FOXM1* expression in HUVECs. Total RNA was isolated from cells 3 d after siRNA transfection.

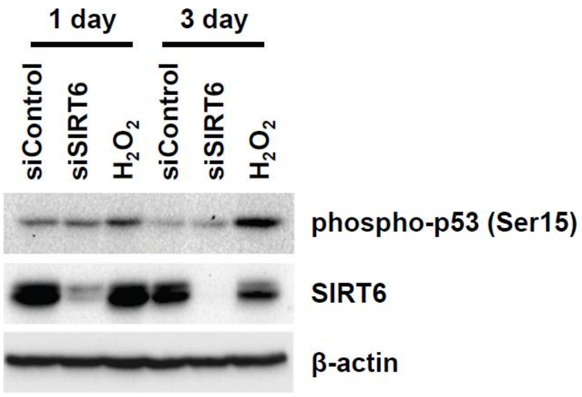

Supplementary Figure 2. Western blot analysis for phosphorylated p53 expressions in *SIRT6* siRNA-treated HUVECs. HUVECs were treated with 200 μM H_2O_2 for 1 h or transfected with 25 nM control or *SIRT6* siRNA. After 1 or 3 d. total protein was isolated from cells. Protein expression was analyzed using anti-phopho-p53 (Ser15) and anti-SIRT6 antibodies. β-Actin was used as a loading control.

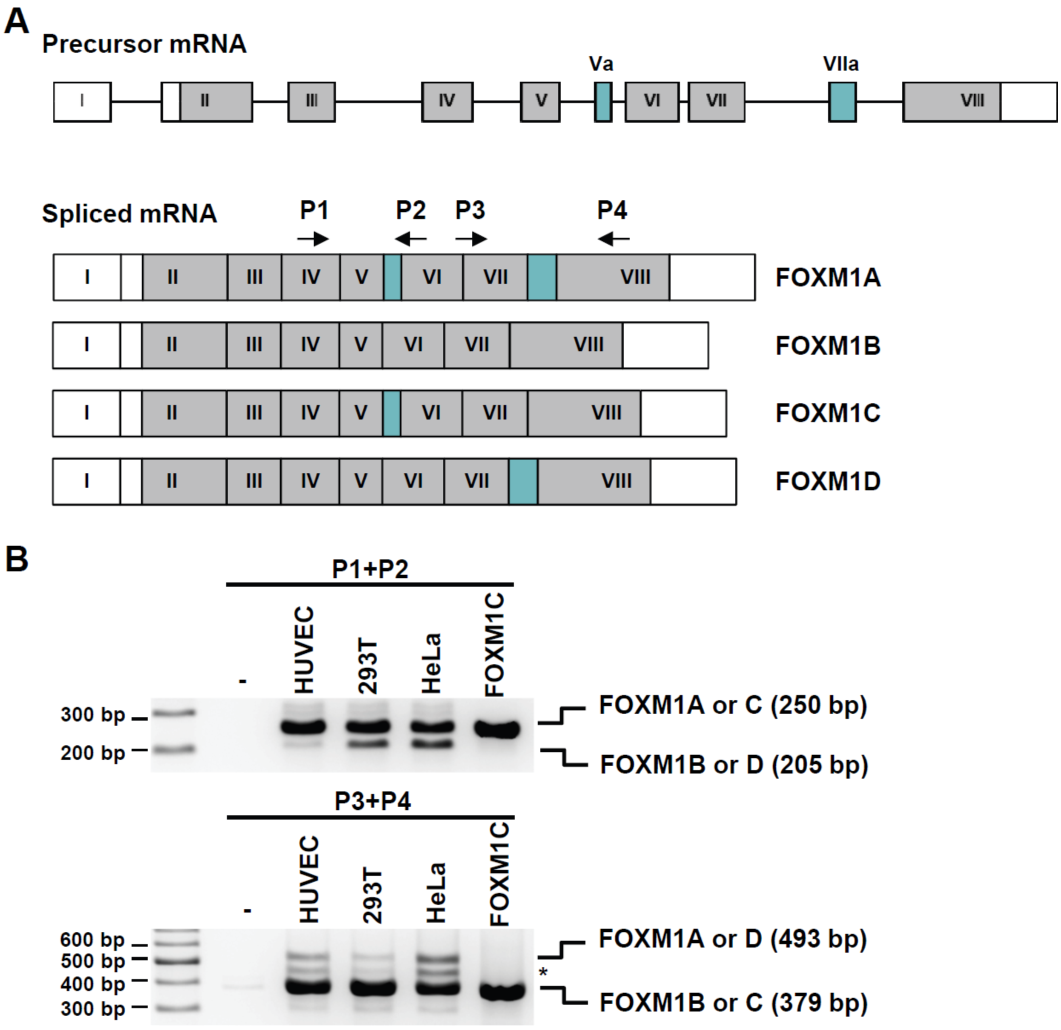

Supplementary Figure 3. FOXM1 isoforms expressed in HUVECs. (**A**) Diagram of FOXM1 precursor and spliced mRNAs. The primers, P1, P2, P3, and P4, to identify the FOXM1 isoforms have been shown. (**B**) RT-PCR analysis showing a 250 bp fragment containing Va exon and a 493 bp fragment containing VIIa exon. Total RNA was isolated from HUVEC, 293T, and HeLa cells. The pENTR/D-TOPO vector containing FOXM1C sequence was used as a control. HUVECs predominantly expressed FOXM1C isoform. *, non-specific PCR fragments.
