# Supplementary material for: Sirtuin 6 deficiency induces endothelial cell senescence via downregulation of forkhead box M1 expression
Source: Aging (Albany NY). 2020 Nov 10;12(21):20946–67. doi: 10.18632/aging.202176 (PMC7695388; doi:10.18632/aging.202176)
Supplement: Supplementary Table 1 [file aging-12-202176-s002..docx]

**Supplementary Table 1. Gene list of differentially expressing genes in SIRT6 siRNA-treated HUVECs compared to control cells.**

|  | Probe ID | Symbol | Genebank accession | siControl | siSIRT6 | Fold ratio (siSIRT6/siControl) |
| --- | --- | --- | --- | --- | --- | --- |
|  | ILMN_1800276 | RCN1 | NM_002901.1 | 7634.58 | 1680.88 | 0.22 |
|  | ILMN_1809850 | RCN3 | NM_020650.2 | 1290.46 | 340.46 | 0.26 |
|  | ILMN_1670948 | TSR2 | NM_058163.1 | 1451.17 | 407.88 | 0.28 |
|  | ILMN_2388155 | CASP3 | NM_032991.2 | 2332.51 | 673.1 | 0.29 |
|  | ILMN_1801842 | PTX3 | NM_002852.2 | 2157.28 | 705.74 | 0.33 |
|  | ILMN_1779014 | TSPYL1 | NM_003309.2 | 919.9 | 322.39 | 0.35 |
|  | ILMN_1746368 | SELT | NM_016275.3 | 716.42 | 265.4 | 0.37 |
|  | ILMN_1739582 | HOXA9 | NM_152739.3 | 2455.84 | 921.39 | 0.38 |
|  | ILMN_1728071 | KRAS | NM_033360.2 | 354.59 | 147.14 | 0.41 |
|  | ILMN_1682139 | RAI14 | NM_015577.1 | 5586.26 | 2437.18 | 0.44 |
|  | ILMN_1725090 | CTHRC1 | NM_138455.2 | 1715.8 | 760.95 | 0.44 |
|  | ILMN_1679267 | TGM2 | NM_198951.1 | 2067.97 | 937.28 | 0.45 |
|  | ILMN_1761912 | MGAT1 | NM_002406.2 | 1135.67 | 513.54 | 0.45 |
|  | ILMN_2227368 | SELT | NM_016275.3 | 450.38 | 205.5 | 0.46 |
|  | ILMN_1781795 | ERH | NM_004450.1 | 4719.22 | 2176.31 | 0.46 |
|  | ILMN_1761594 | FAM35A | NM_019054.2 | 459.53 | 218.22 | 0.47 |
|  | ILMN_1728197 | CLDN5 | NM_003277.2 | 2182.35 | 1040.94 | 0.48 |
|  | ILMN_2394571 | FBXW11 | NM_033645.2 | 1009.9 | 486.51 | 0.48 |
|  | ILMN_3250067 | ANGPT2 | NM_001118888.1 | 1485.09 | 712.95 | 0.48 |
|  | ILMN_1669881 | TSPAN13 | NM_014399.3 | 1063.79 | 513.78 | 0.48 |
|  | ILMN_1715179 | SNRPA1 | NM_003090.2 | 5371.09 | 2598.27 | 0.48 |
|  | ILMN_1792712 | LOC201725 | NM_001008393.1 | 775.69 | 374.2 | 0.48 |
|  | ILMN_1691843 | RNPS1 | NM_080594.1 | 1914.83 | 928.01 | 0.48 |
|  | ILMN_1696568 | ATP2C1 | NM_014382.2 | 523.6 | 257.13 | 0.49 |
|  | ILMN_1683120 | UNG | NM_003362.2 | 2483.8 | 1235.03 | 0.50 |
|  | ILMN_1814526 | ADD3 | NM_001121.2 | 1145.16 | 569.41 | 0.50 |
|  | ILMN_1710676 | FBXO5 | NM_012177.2 | 1225.37 | 615.95 | 0.50 |
|  | ILMN_1762899 | EGR1 | NM_001964.2 | 506 | 254.58 | 0.50 |
|  | ILMN_2117508 | CTHRC1 | NM_138455.2 | 897.02 | 451.01 | 0.50 |
|  | ILMN_3249240 | C4orf46 | NM_001008393.2 | 553.59 | 277.82 | 0.50 |
|  | ILMN_1689037 | LIPG | NM_006033.2 | 1002.93 | 506.94 | 0.51 |
|  | ILMN_1786065 | UHRF1 | NM_001048201.1 | 2699.23 | 1360.89 | 0.50 |
|  | ILMN_2068104 | TFPI2 | NM_006528.2 | 2719.26 | 1373.52 | 0.51 |
|  | ILMN_1710758 | RNF20 | NM_019592.5 | 648.37 | 328.41 | 0.51 |
|  | ILMN_1660718 | GABBR2 | NM_005458.5 | 4021.91 | 2048.47 | 0.51 |
|  | ILMN_1666733 | IL8 | NM_000584.2 | 2686.17 | 1372.57 | 0.51 |
|  | ILMN_1711838 | SLC25A24 | NM_013386.3 | 521.91 | 265.7 | 0.51 |
|  | ILMN_1774207 | ANGPT2 | NM_001147.1 | 2182.85 | 1115.13 | 0.51 |
|  | ILMN_2297626 | PEG10 | NM_001040152.1 | 449.76 | 229.39 | 0.51 |
|  | ILMN_1705908 | RPL7L1 | NM_198486.2 | 2194.48 | 1123.15 | 0.51 |
|  | ILMN_1779711 | DTL | NM_016448.1 | 642.11 | 329.16 | 0.51 |
|  | ILMN_1668814 | CENPM | NM_024053.3 | 382.59 | 197.31 | 0.52 |
|  | ILMN_1652512 | C2CD2 | NM_015500.1 | 1179.81 | 610.42 | 0.52 |
|  | ILMN_1659913 | ISG20 | NM_002201.4 | 504.83 | 261.5 | 0.52 |
|  | ILMN_1737184 | CDCA7 | NM_031942.4 | 1642.11 | 856.32 | 0.52 |
|  | ILMN_1745110 | LAPTM4A | NM_014713.3 | 2025.88 | 1070.94 | 0.53 |

|  | Probe ID | Symbol | Genebank accession | siControl | siSIRT6 | Fold ratio (siSIRT6/siControl) |
| --- | --- | --- | --- | --- | --- | --- |
|  | ILMN_3280735 | LOC643507 | XR_038681.1 | 1121.85 | 593.32 | 0.53 |
|  | ILMN_1702783 | LOC652595 | XM_942117.1 | 1943.35 | 1040.46 | 0.54 |
|  | ILMN_1654690 | CECR5 | NM_033070.2 | 1412.47 | 760.25 | 0.54 |
|  | ILMN_1694711 | MAD2L1BP | NM_014628.2 | 760.43 | 409.3 | 0.54 |
|  | ILMN_2184373 | IL8 | NM_000584.2 | 8017.85 | 4341.57 | 0.54 |
|  | ILMN_2202423 | HELLS | NM_018063.3 | 601.46 | 324.63 | 0.54 |
|  | ILMN_1794863 | CAMK2N1 | NM_018584.5 | 940.1 | 514.13 | 0.55 |
|  | ILMN_2130525 | TSPAN13 | NM_014399.3 | 1330.11 | 725.42 | 0.55 |
|  | ILMN_3269484 | LOC100128899 | XR_039503.1 | 589.63 | 322.54 | 0.55 |
|  | ILMN_3226082 | LOC728620 | XR_037241.1 | 1506.17 | 827.72 | 0.55 |
|  | ILMN_1694603 | SMARCC1 | NM_003074.2 | 2092 | 1153.66 | 0.55 |
|  | ILMN_3221790 | LOC729123 | XR_015896.2 | 906.19 | 500.88 | 0.55 |
|  | ILMN_1810228 | TTF2 | NM_003594.3 | 745.12 | 415.01 | 0.56 |
|  | ILMN_2412860 | MCM4 | NM_182746.1 | 705.09 | 392.62 | 0.56 |
|  | ILMN_1681503 | MCM2 | NM_004526.2 | 613.68 | 345.61 | 0.56 |
|  | ILMN_1782609 | STAG2 | NM_001042750.1 | 811.81 | 455.82 | 0.56 |
|  | ILMN_2409395 | CCNC | NM_001013399.1 | 692.34 | 389.28 | 0.56 |
|  | ILMN_1725244 | HAT1 | NM_003642.2 | 2901 | 1640.59 | 0.57 |
|  | ILMN_1802519 | VPS36 | NM_016075.2 | 537.21 | 303.03 | 0.56 |
|  | ILMN_2413898 | MCM10 | NM_018518.3 | 1333.81 | 752.56 | 0.56 |
|  | ILMN_1654268 | HMGB2 | NM_002129.2 | 1794.04 | 1020.69 | 0.57 |
|  | ILMN_2174574 | HNRNPA3P1 | NR_002726.1 | 584.61 | 332.07 | 0.57 |
|  | ILMN_1708382 | C3orf75 | NM_001031703.2 | 922.88 | 529.44 | 0.57 |
|  | ILMN_1805828 | VRK1 | NM_003384.2 | 838.31 | 482.93 | 0.58 |
|  | ILMN_2192385 | TTC19 | NM_017775.2 | 850.01 | 487.19 | 0.57 |
|  | ILMN_1704537 | PHGDH | NM_006623.2 | 2248.37 | 1299.43 | 0.58 |
|  | ILMN_1670353 | RAD51AP1 | NM_006479.3 | 1263.02 | 732.66 | 0.58 |
|  | ILMN_1680339 | PDGFRL | NM_006207.1 | 491.6 | 285.3 | 0.58 |
|  | ILMN_1683450 | CDCA5 | NM_080668.2 | 1836.4 | 1066.99 | 0.58 |
|  | ILMN_2123567 | SENP2 | NM_021627.2 | 648.07 | 376.54 | 0.58 |
|  | ILMN_2191436 | POLA1 | NM_016937.2 | 356.23 | 207.7 | 0.58 |
|  | ILMN_1744822 | BECN1 | NM_003766.2 | 929.51 | 542.82 | 0.58 |
|  | ILMN_1707493 | SNHG3-RCC1 | NM_001048197.1 | 1344.01 | 788.52 | 0.59 |
|  | ILMN_1778543 | LOC653874 | XM_936215.1 | 1056.44 | 622.39 | 0.59 |
|  | ILMN_1809034 | GATC | NM_176818.1 | 300.18 | 176.27 | 0.59 |
|  | ILMN_2399431 | PRPS2 | NM_001039091.1 | 1013.88 | 595.93 | 0.59 |
|  | ILMN_1673721 | EXO1 | NM_006027.3 | 404.69 | 239.96 | 0.59 |
|  | ILMN_1678669 | RRM2 | NM_001034.1 | 602.3 | 355.49 | 0.59 |
|  | ILMN_1684217 | AURKB | NM_004217.2 | 1511.4 | 896.61 | 0.59 |
|  | ILMN_1705686 | NRGN | NM_006176.1 | 731.98 | 433.73 | 0.59 |
|  | ILMN_1737205 | MCM4 | NM_005914.2 | 1653.15 | 978.21 | 0.59 |
|  | ILMN_1746561 | BCL2L2 | NM_004050.2 | 634.73 | 374.63 | 0.59 |
|  | ILMN_2160929 | FEN1 | NM_004111.4 | 4044.28 | 2387.58 | 0.59 |
|  | ILMN_1740291 | POLQ | NM_199420.3 | 441.62 | 263.26 | 0.60 |
|  | ILMN_1789123 | PLK4 | NM_014264.3 | 1108.45 | 658.79 | 0.59 |

|  | Probe ID | Symbol | Genebank accession | siControl | siSIRT6 | Fold ratio (siSIRT6/siControl) |
| --- | --- | --- | --- | --- | --- | --- |
|  | ILMN_1789384 | QSOX2 | NM_181701.3 | 456.35 | 271.41 | 0.59 |
|  | ILMN_3293146 | LOC642975 | XR_036988.1 | 5376.06 | 3195.15 | 0.59 |
|  | ILMN_1661432 | NUP43 | NM_024647.4 | 1833.44 | 1095.22 | 0.60 |
|  | ILMN_1676423 | CCNC | NM_005190.3 | 867.47 | 518.43 | 0.60 |
|  | ILMN_1693259 | PDCD6IP | NM_013374.3 | 556.15 | 333.53 | 0.60 |
|  | ILMN_2368718 | CENPM | NM_001002876.1 | 868.47 | 519.03 | 0.60 |
|  | ILMN_1693905 | HAT1 | NM_003642.2 | 747.54 | 450.38 | 0.60 |
|  | ILMN_1806312 | C20orf30 | NM_001009924.1 | 4404.21 | 2657.15 | 0.60 |
|  | ILMN_2349459 | BIRC5 | NM_001168.2 | 1950.1 | 1177.9 | 0.60 |
|  | ILMN_3241169 | C3orf75 | NM_001031703.2 | 598.14 | 360.2 | 0.60 |
|  | ILMN_1713249 | PHF19 | NM_015651.1 | 913.76 | 554.74 | 0.61 |
|  | ILMN_1741801 | CDC7 | NM_003503.2 | 654.69 | 397.92 | 0.61 |
|  | ILMN_1776464 | PARP4 | NM_006437.3 | 1986.48 | 1206.82 | 0.61 |
|  | ILMN_2348975 | NASP | NM_002482.2 | 368.62 | 223.94 | 0.61 |
|  | ILMN_1815051 | API5 | NM_006595.2 | 1339.67 | 817.46 | 0.61 |
|  | ILMN_1873278 | LOC731895 | XM_001133620.1 | 343.14 | 209 | 0.61 |
|  | ILMN_2112811 | RPL36A | NM_021029.4 | 570.07 | 346.81 | 0.61 |
|  | ILMN_2188521 | PVRL3 | NM_015480.1 | 935.33 | 569.28 | 0.61 |
|  | ILMN_2222008 | KIFC1 | NM_002263.2 | 1140.67 | 697.64 | 0.61 |
|  | ILMN_1660793 | PAQR4 | NM_152341.2 | 748.05 | 459.53 | 0.61 |
|  | ILMN_1727633 | PVRL3 | NM_015480.1 | 498.23 | 304.79 | 0.61 |
|  | ILMN_1751776 | CKAP2L | NM_152515.2 | 747.71 | 457.83 | 0.61 |
|  | ILMN_1801121 | SENP2 | NM_021627.2 | 394.26 | 241.74 | 0.61 |
|  | ILMN_1805826 | BIVM | NM_017693.2 | 670.46 | 410.81 | 0.61 |
|  | ILMN_2143155 | KIF11 | NM_004523.2 | 1311.8 | 802.86 | 0.61 |
|  | ILMN_3234547 | LOC100133803 | XM_001719081.1 | 3177.48 | 1951.9 | 0.61 |
|  | ILMN_3240389 | CPOX | NM_000097.4 | 928.01 | 569.94 | 0.61 |
|  | ILMN_1700081 | FST | NM_013409.1 | 2579.72 | 1594.62 | 0.62 |
|  | ILMN_1708427 | KPNA3 | NM_002267.2 | 2304.65 | 1425.58 | 0.62 |
|  | ILMN_2121555 | FBXO5 | NM_012177.2 | 624.11 | 386.06 | 0.62 |
|  | ILMN_2396947 | PSMC3IP | NM_016556.1 | 350.68 | 216.42 | 0.62 |
|  | ILMN_3206827 | LOC100131737 | XR_039035.1 | 825.81 | 508.93 | 0.62 |
|  | ILMN_1753342 | SAT1 | NM_002970.1 | 2185.38 | 1358.38 | 0.62 |
|  | ILMN_2189027 | LIPG | NM_006033.2 | 2164.27 | 1345.26 | 0.62 |
|  | ILMN_1659364 | RFC5 | NM_007370.3 | 1106.41 | 693.14 | 0.63 |
|  | ILMN_1668657 | FLJ38973 | NM_153689.4 | 422.65 | 264.11 | 0.62 |
|  | ILMN_1758104 | PRPS2 | NM_001039091.1 | 663.22 | 415.68 | 0.63 |
|  | ILMN_1797236 | TGM2 | NM_198951.1 | 499.84 | 311.7 | 0.62 |
|  | ILMN_1809590 | GINS2 | NM_016095.1 | 1663.11 | 1037.1 | 0.62 |
|  | ILMN_2048700 | ATAD2 | NM_014109.2 | 495.13 | 309.54 | 0.63 |
|  | ILMN_2075334 | HIST1H4C | NM_003542.3 | 15618.84 | 9787.11 | 0.63 |
|  | ILMN_2187830 | CCNH | NM_001239.2 | 2348.19 | 1470.75 | 0.63 |
|  | ILMN_1676893 | ADCY3 | NM_004036.3 | 1528.61 | 963.85 | 0.63 |
|  | ILMN_1799744 | GALC | NM_000153.2 | 394.17 | 247.28 | 0.63 |
|  | ILMN_2187718 | COX17 | NM_005694.1 | 2493 | 1566.87 | 0.63 |
|  | ILMN_2404539 | C20orf30 | NM_001009923.1 | 3604.71 | 2271.87 | 0.63 |

|  | Probe ID | Symbol | Genebank accession | siControl | siSIRT6 | Fold ratio (siSIRT6/siControl) |
| --- | --- | --- | --- | --- | --- | --- |
|  | ILMN_1800276 | RCN1 | NM_002901.1 | 7634.58 | 1680.88 | 0.22 |
|  | ILMN_1809850 | RCN3 | NM_020650.2 | 1290.46 | 340.46 | 0.26 |
|  | ILMN_1670948 | TSR2 | NM_058163.1 | 1451.17 | 407.88 | 0.28 |
|  | ILMN_2388155 | CASP3 | NM_032991.2 | 2332.51 | 673.1 | 0.29 |
|  | ILMN_1801842 | PTX3 | NM_002852.2 | 2157.28 | 705.74 | 0.33 |
|  | ILMN_1779014 | TSPYL1 | NM_003309.2 | 919.9 | 322.39 | 0.35 |
|  | ILMN_1746368 | SELT | NM_016275.3 | 716.42 | 265.4 | 0.37 |
|  | ILMN_1739582 | HOXA9 | NM_152739.3 | 2455.84 | 921.39 | 0.38 |
|  | ILMN_1728071 | KRAS | NM_033360.2 | 354.59 | 147.14 | 0.41 |
|  | ILMN_1682139 | RAI14 | NM_015577.1 | 5586.26 | 2437.18 | 0.44 |
|  | ILMN_1725090 | CTHRC1 | NM_138455.2 | 1715.8 | 760.95 | 0.44 |
|  | ILMN_1679267 | TGM2 | NM_198951.1 | 2067.97 | 937.28 | 0.45 |
|  | ILMN_1761912 | MGAT1 | NM_002406.2 | 1135.67 | 513.54 | 0.45 |
|  | ILMN_2227368 | SELT | NM_016275.3 | 450.38 | 205.5 | 0.46 |
|  | ILMN_1781795 | ERH | NM_004450.1 | 4719.22 | 2176.31 | 0.46 |
|  | ILMN_1761594 | FAM35A | NM_019054.2 | 459.53 | 218.22 | 0.47 |
|  | ILMN_1728197 | CLDN5 | NM_003277.2 | 2182.35 | 1040.94 | 0.48 |
|  | ILMN_2394571 | FBXW11 | NM_033645.2 | 1009.9 | 486.51 | 0.48 |
|  | ILMN_3250067 | ANGPT2 | NM_001118888.1 | 1485.09 | 712.95 | 0.48 |
|  | ILMN_1669881 | TSPAN13 | NM_014399.3 | 1063.79 | 513.78 | 0.48 |
|  | ILMN_1715179 | SNRPA1 | NM_003090.2 | 5371.09 | 2598.27 | 0.48 |
|  | ILMN_1792712 | LOC201725 | NM_001008393.1 | 775.69 | 374.2 | 0.48 |
|  | ILMN_1691843 | RNPS1 | NM_080594.1 | 1914.83 | 928.01 | 0.48 |
|  | ILMN_1696568 | ATP2C1 | NM_014382.2 | 523.6 | 257.13 | 0.49 |
|  | ILMN_1683120 | UNG | NM_003362.2 | 2483.8 | 1235.03 | 0.50 |
|  | ILMN_1814526 | ADD3 | NM_001121.2 | 1145.16 | 569.41 | 0.50 |
|  | ILMN_1710676 | FBXO5 | NM_012177.2 | 1225.37 | 615.95 | 0.50 |
|  | ILMN_1762899 | EGR1 | NM_001964.2 | 506 | 254.58 | 0.50 |
|  | ILMN_2117508 | CTHRC1 | NM_138455.2 | 897.02 | 451.01 | 0.50 |
|  | ILMN_3249240 | C4orf46 | NM_001008393.2 | 553.59 | 277.82 | 0.50 |
|  | ILMN_1689037 | LIPG | NM_006033.2 | 1002.93 | 506.94 | 0.51 |
|  | ILMN_1786065 | UHRF1 | NM_001048201.1 | 2699.23 | 1360.89 | 0.50 |
|  | ILMN_2068104 | TFPI2 | NM_006528.2 | 2719.26 | 1373.52 | 0.51 |
|  | ILMN_1710758 | RNF20 | NM_019592.5 | 648.37 | 328.41 | 0.51 |
|  | ILMN_1660718 | GABBR2 | NM_005458.5 | 4021.91 | 2048.47 | 0.51 |
|  | ILMN_1666733 | IL8 | NM_000584.2 | 2686.17 | 1372.57 | 0.51 |
|  | ILMN_1711838 | SLC25A24 | NM_013386.3 | 521.91 | 265.7 | 0.51 |
|  | ILMN_1774207 | ANGPT2 | NM_001147.1 | 2182.85 | 1115.13 | 0.51 |
|  | ILMN_2297626 | PEG10 | NM_001040152.1 | 449.76 | 229.39 | 0.51 |
|  | ILMN_1705908 | RPL7L1 | NM_198486.2 | 2194.48 | 1123.15 | 0.51 |
|  | ILMN_1779711 | DTL | NM_016448.1 | 642.11 | 329.16 | 0.51 |
|  | ILMN_1668814 | CENPM | NM_024053.3 | 382.59 | 197.31 | 0.52 |
|  | ILMN_1652512 | C2CD2 | NM_015500.1 | 1179.81 | 610.42 | 0.52 |
|  | ILMN_1659913 | ISG20 | NM_002201.4 | 504.83 | 261.5 | 0.52 |
|  | ILMN_1737184 | CDCA7 | NM_031942.4 | 1642.11 | 856.32 | 0.52 |
|  | ILMN_1745110 | LAPTM4A | NM_014713.3 | 2025.88 | 1070.94 | 0.53 |

|  | Probe ID | Symbol | Genebank accession | siControl | siSIRT6 | Fold ratio (siSIRT6/siControl) |
| --- | --- | --- | --- | --- | --- | --- |
|  | ILMN_2412384 | CCNE2 | NM_057735.1 | 591.95 | 372.65 | 0.63 |
|  | ILMN_1658071 | ATP1B1 | NM_001677.3 | 1323.06 | 838.89 | 0.63 |
|  | ILMN_1667225 | PDE1C | NM_005020.1 | 588.81 | 372.39 | 0.63 |
|  | ILMN_1691112 | PIGN | NM_176787.4 | 384.99 | 243.26 | 0.63 |
|  | ILMN_1732688 | DUT | NM_001025248.1 | 775.33 | 490.12 | 0.63 |
|  | ILMN_1806037 | TK1 | NM_003258.2 | 1540.66 | 977.98 | 0.63 |
|  | ILMN_2157099 | CCNA1 | NM_003914.2 | 587.73 | 372.39 | 0.63 |
|  | ILMN_1656574 | PCGF6 | NM_032154.3 | 593.46 | 378.99 | 0.64 |
|  | ILMN_1681737 | TMSB15A | NM_021992.2 | 432.13 | 275.71 | 0.64 |
|  | ILMN_1695414 | ASF1B | NM_018154.2 | 515.92 | 329.62 | 0.64 |
|  | ILMN_1723486 | HK2 | NM_000189.4 | 512.47 | 327.27 | 0.64 |
|  | ILMN_1743397 | PIGW | NM_178517.3 | 552.05 | 351.16 | 0.64 |
|  | ILMN_1760412 | SHISA2 | NM_001007538.1 | 1270.05 | 810.12 | 0.64 |
|  | ILMN_1813207 | MRPS9 | NM_182640.1 | 923.73 | 587.86 | 0.64 |
|  | ILMN_2407824 | ATP1B1 | NM_001001787.1 | 2804.12 | 1783.71 | 0.64 |
|  | ILMN_1675626 | PRPF38A | NM_032864.3 | 360.37 | 230.29 | 0.64 |
|  | ILMN_1721876 | TIMP2 | NM_003255.4 | 1692.18 | 1081.39 | 0.64 |
|  | ILMN_1728972 | FAM64A | NM_019013.1 | 710.65 | 456.56 | 0.64 |
|  | ILMN_1758852 | ENTPD7 | NM_020354.2 | 241.58 | 155.02 | 0.64 |
|  | ILMN_1763907 | C6orf173 | NM_001012507.1 | 4932.15 | 3156.99 | 0.64 |
|  | ILMN_1775170 | MT1X | NM_005952.2 | 1125.75 | 721.74 | 0.64 |
|  | ILMN_1782419 | GNG11 | NM_004126.3 | 10854.46 | 6978.33 | 0.64 |
|  | ILMN_1797074 | EMG1 | NM_006331.5 | 1835.13 | 1176.54 | 0.64 |
|  | ILMN_1798654 | MCM6 | NM_005915.4 | 3142.43 | 2019.8 | 0.64 |
|  | ILMN_1679438 | MLF1IP | NM_024629.2 | 288.95 | 186.58 | 0.65 |
|  | ILMN_1750722 | RPS7 | NM_001011.3 | 1450.16 | 938.14 | 0.65 |
|  | ILMN_1766658 | PKMYT1 | NM_182687.1 | 353.69 | 227.81 | 0.64 |
|  | ILMN_1771966 | BCCIP | NM_078468.1 | 4062.07 | 2621.78 | 0.65 |
|  | ILMN_1794539 | KIF11 | NM_004523.2 | 621.81 | 400.78 | 0.64 |
|  | ILMN_1798454 | MAD2L1BP | NM_014628.2 | 324.41 | 209.24 | 0.64 |
|  | ILMN_2214098 | BIVM | NM_017693.2 | 584.07 | 376.37 | 0.64 |
|  | ILMN_2309926 | ZDHHC14 | NM_024630.2 | 389.37 | 251.72 | 0.65 |
|  | ILMN_1703697 | LANCL1 | NM_006055.1 | 654.54 | 425.2 | 0.65 |
|  | ILMN_1716382 | LOC387882 | NM_207376.1 | 2246.82 | 1454.86 | 0.65 |
|  | ILMN_1655694 | LOC642031 | XM_936101.2 | 799.9 | 522.64 | 0.65 |
|  | ILMN_1656274 | PRPF38A | NM_032864.3 | 422.65 | 275.9 | 0.65 |
|  | ILMN_1716279 | CENPE | NM_001813.2 | 996.69 | 649.87 | 0.65 |
|  | ILMN_1735908 | UTP15 | NM_032175.2 | 307.9 | 201.5 | 0.65 |
|  | ILMN_1739942 | FAM117B | NM_173511.2 | 360.54 | 235.62 | 0.65 |
|  | ILMN_1776577 | DSCC1 | NM_024094.1 | 359.54 | 235.46 | 0.65 |
|  | ILMN_1810604 | ELMOD1 | NM_018712.2 | 317.88 | 207.65 | 0.65 |
|  | ILMN_2396948 | PSMC3IP | NM_016556.1 | 256.12 | 167.5 | 0.65 |
|  | ILMN_2401701 | PCGF6 | NM_032154.3 | 678.87 | 444.49 | 0.65 |
|  | ILMN_2413251 | EWSR1 | NM_013986.2 | 909.54 | 593.18 | 0.65 |
|  | ILMN_2413899 | MCM10 | NM_018518.3 | 322.69 | 210.55 | 0.65 |
|  | ILMN_1709728 | SLC30A5 | NM_022902.2 | 1220.28 | 805.09 | 0.66 |

|  | Probe ID | Symbol | Genebank accession | siControl | siSIRT6 | Fold ratio (siSIRT6/siControl) |
| --- | --- | --- | --- | --- | --- | --- |
|  | ILMN_1726064 | PAK1IP1 | NM_017906.2 | 2051.31 | 1350.86 | 0.66 |
|  | ILMN_1752520 | SLFN11 | NM_152270.2 | 2339.53 | 1540.3 | 0.66 |
|  | ILMN_1767662 | LASS6 | NM_203463.1 | 987.06 | 648.97 | 0.66 |
|  | ILMN_1779264 | PSMG1 | NM_003720.2 | 2700.48 | 1777.95 | 0.66 |
|  | ILMN_1786197 | NR2F1 | NM_005654.4 | 1035.18 | 681.23 | 0.66 |
|  | ILMN_1897741 |  | AV737943 | 880.8 | 580.84 | 0.66 |
|  | ILMN_2086064 | SNRPC | NM_003093.1 | 2725.55 | 1796.12 | 0.66 |
|  | ILMN_2181432 | SPC24 | NM_182513.1 | 1019.51 | 672.47 | 0.66 |
|  | ILMN_2219556 | ISCA1 | NM_030940.3 | 913.76 | 600.91 | 0.66 |
|  | ILMN_2220184 | GFPT1 | NM_002056.1 | 525.06 | 346.49 | 0.66 |
|  | ILMN_3232696 | LOC729816 | XR_042352.1 | 2451.3 | 1613.15 | 0.66 |
|  | ILMN_1667670 | SLC25A15 | NM_014252.1 | 633.85 | 418.96 | 0.66 |
|  | ILMN_1674231 | CHAF1B | NM_005441.2 | 538.45 | 356.81 | 0.66 |
|  | ILMN_1696302 | FABP5 | NM_001444.1 | 3937.32 | 2615.73 | 0.66 |
|  | ILMN_1705682 | LEPREL2 | NM_014262.2 | 705.91 | 468.31 | 0.66 |
|  | ILMN_1748923 | SMC2 | NM_001042550.1 | 527.86 | 348.9 | 0.66 |
|  | ILMN_1790778 | PNMA2 | NM_007257.4 | 2296.15 | 1518.75 | 0.66 |
|  | ILMN_1796589 | TRIP13 | NM_004237.2 | 2212.3 | 1464.98 | 0.66 |
|  | ILMN_2070072 | RPS7 | NM_001011.3 | 1072.18 | 710.65 | 0.66 |
|  | ILMN_3244963 | WDR42A | NM_015726.2 | 545.59 | 362.46 | 0.66 |
|  | ILMN_1658027 | RAD54L | NM_003579.2 | 332.53 | 222.04 | 0.67 |
|  | ILMN_1779852 | LOC387934 | XM_937508.2 | 4856.38 | 3230.03 | 0.67 |
|  | ILMN_1908989 |  | AI557007 | 484.94 | 322.24 | 0.66 |
|  | ILMN_2146761 | FABP5 | NM_001444.1 | 2571.99 | 1710.26 | 0.66 |
|  | ILMN_2162860 | SLFN11 | NM_152270.2 | 1673.13 | 1117.97 | 0.67 |
|  | ILMN_2395856 | TMEM218 | NM_001080546.1 | 994.62 | 664.6 | 0.67 |
|  | ILMN_3238859 | FAM120AOS | NM_198841.2 | 1375.75 | 914.39 | 0.66 |
|  | ILMN_3266606 | FABP5L2 | XM_001134012.2 | 8480.89 | 5649.86 | 0.67 |
|  | ILMN_1660305 | PDE12 | NM_177966.4 | 539.57 | 363.13 | 0.67 |
|  | ILMN_1663195 | MCM7 | NM_182776.1 | 5550.24 | 3719.78 | 0.67 |
|  | ILMN_1669550 | MAD2L2 | NM_006341.2 | 1432.84 | 962.29 | 0.67 |
|  | ILMN_1684802 | TAF5 | NM_006951.3 | 384.63 | 258.02 | 0.67 |
|  | ILMN_1689329 | SCD | NM_005063.4 | 5943.1 | 3983.99 | 0.67 |
|  | ILMN_1691466 | YES1 | NM_005433.3 | 4878.88 | 3278.91 | 0.67 |
|  | ILMN_1696975 | USP1 | NM_001017416.1 | 1409.53 | 944.67 | 0.67 |
|  | ILMN_1706687 | KLHL5 | NM_001007075.1 | 1301.24 | 874.92 | 0.67 |
|  | ILMN_1712386 | C21orf45 | NM_018944.2 | 731.31 | 491.48 | 0.67 |
|  | ILMN_2056760 | MKRN2 | NM_014160.3 | 1123.67 | 754.48 | 0.67 |
|  | ILMN_2211672 | TSNAX | NM_005999.2 | 592.36 | 397.09 | 0.67 |
|  | ILMN_2220403 | C6orf72 | NM_138785.1 | 401.71 | 269.16 | 0.67 |
|  | ILMN_2288784 | CCDC34 | NM_030771.1 | 768.73 | 517.11 | 0.67 |
|  | ILMN_1664956 | SSU72 | NM_014188.2 | 5529.76 | 3725.8 | 0.67 |
|  | ILMN_1678962 | DFFB | NM_004402.2 | 297.62 | 200.9 | 0.68 |
|  | ILMN_1682717 | IER3 | NM_003897.3 | 3165.02 | 2134.97 | 0.67 |
|  | ILMN_1693410 | BRI3BP | XM_941876.1 | 867.67 | 585.15 | 0.67 |
|  | ILMN_1706558 | FLJ20628 | NM_017910.2 | 763.42 | 515.2 | 0.67 |

|  | Probe ID | Symbol | Genebank accession | siControl | siSIRT6 | Fold ratio (siSIRT6/siControl) |
| --- | --- | --- | --- | --- | --- | --- |
|  | ILMN_1725510 | DHCR24 | NM_014762.3 | 2326.59 | 1569.4 | 0.67 |
|  | ILMN_1728047 | AKR1A1 | NM_006066.2 | 1627.38 | 1102.83 | 0.68 |
|  | ILMN_1740430 | SLC2A4RG | NM_020062.3 | 373.08 | 251.72 | 0.67 |
|  | ILMN_1751753 | IDH2 | NM_002168.2 | 812 | 546.98 | 0.67 |
|  | ILMN_1753063 | KIF15 | NM_020242.1 | 454.67 | 307.33 | 0.68 |
|  | ILMN_1777564 | MAD2L1 | NM_002358.2 | 2524.88 | 1701.59 | 0.67 |
|  | ILMN_1786433 | BCCIP | NM_016567.2 | 581.11 | 393.26 | 0.68 |
|  | ILMN_1788184 | CIDEA | NM_001279.2 | 316.71 | 214.33 | 0.68 |
|  | ILMN_1796119 | ZUFSP | NM_145062.1 | 390.09 | 263.32 | 0.68 |
|  | ILMN_1813028 | CBX5 | NM_012117.1 | 817.84 | 550.78 | 0.67 |
|  | ILMN_2147471 | PI4K2B | NM_018323.2 | 403.85 | 273.61 | 0.68 |
|  | ILMN_2224143 | MCM3 | NM_002388.3 | 5325.37 | 3597.22 | 0.68 |
|  | ILMN_2313672 | IL1RL1 | NM_003856.2 | 5545.11 | 3735.28 | 0.67 |
|  | ILMN_2396002 | MRPL10 | NM_145255.2 | 887.74 | 599.94 | 0.68 |
|  | ILMN_3270641 | HNRNPH3 | NM_012207.2 | 1456.88 | 987.06 | 0.68 |
|  | ILMN_1667839 | UBR7 | NM_001100417.1 | 1032.31 | 700.22 | 0.68 |
|  | ILMN_1690138 | PHF10 | NM_133325.1 | 350.43 | 239.02 | 0.68 |
|  | ILMN_1711470 | UBE2T | NM_014176.2 | 1932.61 | 1310.89 | 0.68 |
|  | ILMN_1717324 | C16orf52 | NM_173501.1 | 361.87 | 246.31 | 0.68 |
|  | ILMN_1747183 | GXYLT1 | NM_001099650.1 | 411 | 279.43 | 0.68 |
|  | ILMN_1750969 | C9orf10OS | NM_198841.1 | 568.49 | 387.13 | 0.68 |
|  | ILMN_1755834 | FEN1 | NM_004111.4 | 650.47 | 441.01 | 0.68 |
|  | ILMN_1794914 | UBTD1 | NM_024954.3 | 801.93 | 545.34 | 0.68 |
|  | ILMN_1806818 | MCM3 | NM_002388.3 | 1015.28 | 691.22 | 0.68 |
|  | ILMN_1808591 | LOC731049 | XM_001129232.1 | 4512.36 | 3064.99 | 0.68 |
|  | ILMN_2156172 | HK2 | NM_000189.4 | 630.49 | 428.06 | 0.68 |
|  | ILMN_2161577 | CXCL6 | NM_002993.2 | 318.1 | 215.97 | 0.68 |
|  | ILMN_2220187 | GFPT1 | NM_002056.1 | 1191.04 | 807.7 | 0.68 |
|  | ILMN_2363621 | RBBP8 | NM_203291.1 | 782.89 | 533.74 | 0.68 |
|  | ILMN_3289262 | LOC100131261 | XM_001723141.1 | 2704.85 | 1840.23 | 0.68 |
|  | ILMN_1651936 | SETD8 | NM_020382.3 | 528.83 | 361.95 | 0.68 |
|  | ILMN_1660806 | CSRP2 | NM_001321.1 | 449.96 | 307.55 | 0.68 |
|  | ILMN_1714433 | MARCKSL1 | NM_023009.4 | 2696.12 | 1842.78 | 0.68 |
|  | ILMN_1717765 | NUDT11 | NM_018159.3 | 613.25 | 421 | 0.69 |
|  | ILMN_1718853 | UQCRC2 | NM_003366.2 | 2284.51 | 1563.61 | 0.68 |
|  | ILMN_1728626 | WDR5 | NM_017588.2 | 470.7 | 322.54 | 0.69 |
|  | ILMN_1783170 | ING3 | NM_198267.1 | 373.34 | 256.3 | 0.69 |
|  | ILMN_1811188 | BCCIP | NM_016567.2 | 1314.53 | 902.84 | 0.69 |
|  | ILMN_2066124 | AFG3L2 | NM_006796.1 | 1171.39 | 803.23 | 0.69 |
|  | ILMN_3210741 | LOC642956 | XM_938166.3 | 1741.36 | 1190.21 | 0.68 |
|  | ILMN_3238623 | LYRM7 | NM_181705.2 | 561.44 | 383.66 | 0.68 |
|  | ILMN_1658464 | GTF3A | NM_002097.1 | 3821.7 | 2641.24 | 0.69 |
|  | ILMN_1695422 | NCL | NM_005381.2 | 964.07 | 664.29 | 0.69 |
|  | ILMN_1709294 | CDCA8 | NM_018101.2 | 879.98 | 606.49 | 0.69 |
|  | ILMN_1732150 | KIAA0101 | NM_001029989.1 | 274.25 | 189.23 | 0.69 |
|  | ILMN_1738712 | GPR180 | NM_180989.4 | 892.27 | 615.95 | 0.69 |

|  | Probe ID | Symbol | Genebank accession | siControl | siSIRT6 | Fold ratio (siSIRT6/siControl) |
| --- | --- | --- | --- | --- | --- | --- |
|  | ILMN_1753639 | MTAP | NM_002451.3 | 682.96 | 470.37 | 0.69 |
|  | ILMN_1784410 | UBP1 | NM_014517.3 | 1951 | 1341.84 | 0.69 |
|  | ILMN_1790100 | C11orf82 | NM_145018.2 | 593.59 | 410.62 | 0.69 |
|  | ILMN_2103685 | DEPDC1B | NM_018369.1 | 750.83 | 518.19 | 0.69 |
|  | ILMN_3307266 | PSMC3IP | NM_013290.5 | 292.78 | 201.64 | 0.69 |
|  | ILMN_1662316 | VPS33A | NM_022916.4 | 305.35 | 212.31 | 0.70 |
|  | ILMN_1692826 | UNKL | NM_023076.3 | 367.52 | 254.94 | 0.69 |
|  | ILMN_1698252 | FANCB | NM_152633.2 | 257.72 | 178.44 | 0.69 |
|  | ILMN_1702197 | C9orf140 | NM_178448.2 | 722.24 | 502.74 | 0.70 |
|  | ILMN_1702683 | SLC33A1 | NM_004733.2 | 556.92 | 386.32 | 0.69 |
|  | ILMN_1730291 | ATP1B1 | NM_001677.3 | 1504.43 | 1042.38 | 0.69 |
|  | ILMN_1745599 | LOC643977 | XM_932991.1 | 318.98 | 221.78 | 0.70 |
|  | ILMN_1754045 | FANCL | NM_018062.2 | 346.73 | 240.02 | 0.69 |
|  | ILMN_1758164 | STC1 | NM_003155.2 | 435.94 | 301.85 | 0.69 |
|  | ILMN_1762678 | NMT1 | NM_021079.3 | 369.48 | 256.3 | 0.69 |
|  | ILMN_1771593 | RRM1 | NM_001033.2 | 4767.44 | 3309.35 | 0.69 |
|  | ILMN_1779547 | HPSE | NM_006665.2 | 603.55 | 418.09 | 0.69 |
|  | ILMN_1781906 | RBM17 | NM_032905.3 | 782.89 | 541.82 | 0.69 |
|  | ILMN_1792455 | TMEM158 | NM_015444.2 | 5292.25 | 3662.64 | 0.69 |
|  | ILMN_1798014 | EIF2S2 | NM_003908.3 | 290.76 | 201.97 | 0.69 |
|  | ILMN_1809439 | HMGB1L1 | NM_001008735.1 | 7543.41 | 5243.57 | 0.70 |
|  | ILMN_1853881 |  | BG118017 | 262.41 | 182.45 | 0.70 |
|  | ILMN_2089902 | NUS1 | NM_138459.3 | 354.01 | 245.06 | 0.69 |
|  | ILMN_2093027 | MYO1B | NM_012223.2 | 1370.04 | 949.05 | 0.69 |
|  | ILMN_2137084 | LIN9 | NM_173083.2 | 282.09 | 196.04 | 0.69 |
|  | ILMN_2212909 | MELK | NM_014791.2 | 2294.56 | 1590.94 | 0.69 |
|  | ILMN_2334205 | ERCC6L | NM_017669.2 | 415.78 | 288.95 | 0.69 |
|  | ILMN_2380771 | AKR1A1 | NM_153326.1 | 2409.19 | 1675.06 | 0.70 |
|  | ILMN_3178258 | FABP5L2 | XM_001721172.1 | 2829.5 | 1958.23 | 0.69 |
|  | ILMN_3239771 | DLGAP5 | NM_014750.3 | 2471.21 | 1721.76 | 0.70 |
|  | ILMN_1654966 | SCARA3 | NM_182826.1 | 239.69 | 167.81 | 0.70 |
|  | ILMN_1754051 | RMI1 | NM_024945.2 | 426.48 | 299.21 | 0.70 |
|  | ILMN_1761010 | PCCB | NM_000532.3 | 1643.25 | 1149.4 | 0.70 |
|  | ILMN_1763663 | FLJ20718 | NM_017939.1 | 924.16 | 646.42 | 0.70 |
|  | ILMN_1786125 | CCNA2 | NM_001237.2 | 2090.07 | 1461.6 | 0.70 |
|  | ILMN_1787628 | NOP56 | NM_006392.2 | 7010.65 | 4915.08 | 0.70 |
|  | ILMN_1806951 | CSTF3 | NM_001033506.1 | 279.04 | 194.64 | 0.70 |
|  | ILMN_1810608 | PNPT1 | NM_033109.2 | 341.72 | 238.8 | 0.70 |
|  | ILMN_2112599 | C16orf80 | NM_013242.2 | 1368.77 | 954.1 | 0.70 |
|  | ILMN_2215640 | TUBA3D | NM_080386.1 | 8082.95 | 5643.34 | 0.70 |
|  | ILMN_2374778 | DUT | NM_001025249.1 | 452.78 | 316.19 | 0.70 |
|  | ILMN_3290261 | LOC644877 | XR_017355.2 | 926.08 | 646.87 | 0.70 |
|  | ILMN_1654920 | HNRPH3 | NM_012207.1 | 2059.39 | 1446.48 | 0.70 |
|  | ILMN_1663648 | MTUS1 | NM_020749.3 | 1043.1 | 735.37 | 0.70 |
|  | ILMN_1664516 | CENPF | NM_016343.3 | 521.79 | 367.77 | 0.70 |
|  | ILMN_1665559 | CDK2 | NM_001798.2 | 1112.3 | 785.25 | 0.71 |

|  | Probe ID | Symbol | Genebank accession | siControl | siSIRT6 | Fold ratio (siSIRT6/siControl) |
| --- | --- | --- | --- | --- | --- | --- |
|  | **ILMN_1697469** | **SFRS6** | **NM_006275.4** | **4909.41** | **3449.09** | **0.70** |
|  | ILMN_1739854 | C15orf29 | NM_024713.1 | 406.28 | 286.16 | 0.70 |
|  | ILMN_1749405 | KIAA1191 | NM_001079685.1 | 1489.56 | 1049.87 | 0.70 |
|  | ILMN_1764362 | LYAR | NM_017816.1 | 1794.87 | 1263.02 | 0.70 |
|  | ILMN_1764500 | C3orf10 | NM_018462.4 | 1407.9 | 989.8 | 0.70 |
|  | ILMN_1778831 | NTSR1 | NM_002531.2 | 270.47 | 191.12 | 0.71 |
|  | ILMN_1785336 | PMM2 | NM_000303.1 | 716.59 | 505.42 | 0.71 |
|  | ILMN_1807177 | KIAA1797 | NM_017794.2 | 432.53 | 304.23 | 0.70 |
|  | ILMN_1807359 | CLEC11A | NM_002975.2 | 693.46 | 489.9 | 0.71 |
|  | ILMN_2081988 | LANCL1 | NM_006055.1 | 974.83 | 688.35 | 0.71 |
|  | ILMN_2126706 | LMNB1 | NM_005573.2 | 851.58 | 600.91 | 0.71 |
|  | ILMN_2143250 | FAR1 | NM_032228.4 | 697.15 | 490.46 | 0.70 |
|  | ILMN_2206953 | PLCL1 | NM_006226.1 | 380.83 | 269.04 | 0.71 |
|  | ILMN_2242900 | IL1RL1 | NM_003856.2 | 3385.14 | 2379.87 | 0.70 |
|  | ILMN_3262936 | LCLAT1 | NM_182551.3 | 510.23 | 359.45 | 0.70 |
|  | ILMN_3304887 | LOC729423 | XM_001726948.1 | 1196 | 841.41 | 0.70 |
|  | ILMN_1655405 | SCARF2 | NM_153334.3 | 243.31 | 172.01 | 0.71 |
|  | ILMN_1658416 | MRPS18C | NM_016067.1 | 3469.07 | 2464.93 | 0.71 |
|  | ILMN_1686109 | CCL23 | NM_145898.1 | 222.76 | 158.1 | 0.71 |
|  | ILMN_1686989 | INSIG1 | NM_198336.1 | 326.44 | 232.11 | 0.71 |
|  | ILMN_1697420 | TINF2 | NM_012461.1 | 934.25 | 664.29 | 0.71 |
|  | ILMN_1698179 | TAGLN3 | NM_001008273.1 | 190.28 | 134.74 | 0.71 |
|  | ILMN_1703906 | HJURP | NM_018410.3 | 610.42 | 434.34 | 0.71 |
|  | ILMN_1712896 | FST | NM_006350.2 | 437.66 | 310.83 | 0.71 |
|  | ILMN_1729115 | LOC651816 | XM_941060.1 | 2771.27 | 1958.68 | 0.71 |
|  | ILMN_1732831 | CHST7 | NM_019886.2 | 826.76 | 586.91 | 0.71 |
|  | ILMN_1749829 | DLGAP5 | NM_014750.3 | 3129.39 | 2223.58 | 0.71 |
|  | ILMN_1766408 | CBFB | NM_001755.2 | 1277.7 | 908.07 | 0.71 |
|  | ILMN_1777233 | E2F2 | NM_004091.2 | 280.59 | 199.33 | 0.71 |
|  | ILMN_1781516 | SUPT16H | NM_007192.2 | 2684.93 | 1908.65 | 0.71 |
|  | ILMN_1783771 | UBE2Z | XM_945544.1 | 291.7 | 206.26 | 0.71 |
|  | ILMN_1784860 | RFC3 | NM_002915.3 | 460.59 | 326.97 | 0.71 |
|  | ILMN_1801664 | POLR3K | NM_016310.2 | 565.87 | 400.69 | 0.71 |
|  | ILMN_1806040 | TYMS | NM_001071.1 | 5868.06 | 4154.14 | 0.71 |
|  | ILMN_1809813 | PGF | NM_002632.4 | 857.31 | 606.77 | 0.71 |
|  | ILMN_2100689 | MAP2K4 | NM_003010.2 | 374.72 | 265.27 | 0.71 |
|  | ILMN_2210129 | PRIM1 | NM_000946.2 | 704.44 | 498.23 | 0.71 |
|  | ILMN_3250879 | ANGPT2 | NM_001118887.1 | 272.73 | 193.21 | 0.71 |
|  | ILMN_3251592 | C9orf140 | NM_178448.3 | 428.95 | 304.58 | 0.71 |
|  | ILMN_1651433 | DCK | NM_000788.1 | 523.12 | 374.63 | 0.72 |
|  | ILMN_1657547 | CCDC34 | NM_030771.1 | 1250.83 | 895.99 | 0.72 |
|  | ILMN_1664434 | TCF3 | NM_003200.1 | 502.74 | 358.71 | 0.71 |
|  | ILMN_1670238 | CDC45L | NM_003504.3 | 1859.46 | 1331.96 | 0.72 |
|  | ILMN_1730824 | ZNF512 | NM_032434.2 | 760.25 | 543.83 | 0.72 |
|  | ILMN_1741599 | MEMO1 | NM_015955.2 | 558.08 | 399.02 | 0.71 |
|  | ILMN_1752802 | CLPTM1L | NM_030782.3 | 1847.89 | 1315.44 | 0.71 |
|  | ILMN_1769702 | GPAA1 | NM_003801.3 | 577.5 | 411.67 | 0.71 |
|  | ILMN_1798212 | LLGL1 | NM_004140.3 | 515.32 | 368.62 | 0.72 |
|  | ILMN_1810992 | CAD | NM_004341.3 | 406.94 | 290.09 | 0.71 |
|  | Probe ID | Symbol | Genebank accession | siControl | siSIRT6 | Fold ratio (siSIRT6/siControl) |
|  | ILMN_1818617 |  | AL133627 | 233.29 | 166.8 | 0.71 |
|  | ILMN_1879857 |  | CB160856 | 199.37 | 142.22 | 0.71 |
|  | ILMN_2090397 | ISG20L2 | NM_030980.1 | 1095.47 | 780.72 | 0.71 |
|  | ILMN_2189668 | NUDT11 | NM_018159.2 | 757.62 | 541.57 | 0.71 |
|  | ILMN_2225718 | CENPE | NM_001813.2 | 656.2 | 470.37 | 0.72 |
|  | ILMN_2368721 | CENPM | NM_001002876.1 | 235.08 | 168.08 | 0.71 |
|  | ILMN_2384513 | C2CD2 | NM_199050.2 | 244.21 | 174.93 | 0.72 |
|  | ILMN_2384785 | CCNE1 | NM_057182.1 | 297.97 | 213.09 | 0.72 |
|  | ILMN_2411190 | SMC2 | NM_001042550.1 | 446.96 | 318.61 | 0.71 |
|  | ILMN_3187771 | C14orf167 | NR_023922.1 | 289.42 | 206.55 | 0.71 |
|  | ILMN_3239621 | SNRNP27 | NM_006857.1 | 1341.84 | 957.19 | 0.71 |
|  | ILMN_1680831 | BAZ1B | NM_023005.2 | 823.33 | 590.58 | 0.72 |
|  | ILMN_1689327 | LOC730534 | XR_015150.1 | 8088.56 | 5827.52 | 0.72 |
|  | ILMN_1694502 | PRIM1 | NM_000946.2 | 538.7 | 386.32 | 0.72 |
|  | ILMN_1698463 | ILF3 | NM_012218.2 | 771.4 | 554.74 | 0.72 |
|  | ILMN_1698551 | RCN1 | NM_002901.1 | 174.29 | 125.11 | 0.72 |
|  | ILMN_1707748 | PIM3 | XM_938171.2 | 610.56 | 438.17 | 0.72 |
|  | ILMN_1710124 | CMTM8 | NM_178868.3 | 575.36 | 413.29 | 0.72 |
|  | ILMN_1713668 | TSNAX | NM_005999.2 | 507.99 | 366.08 | 0.72 |
|  | ILMN_1715905 | DSN1 | NM_024918.2 | 684.23 | 491.48 | 0.72 |
|  | ILMN_1732555 | B4GALT6 | NM_004775.2 | 270.35 | 194.28 | 0.72 |
|  | ILMN_1776052 | LOC148915 | XM_937758.1 | 4582.76 | 3307.82 | 0.72 |
|  | ILMN_1783753 | TXNDC12 | NM_015913.2 | 2618.15 | 1878.89 | 0.72 |
|  | ILMN_1785756 | LOC731314 | XM_001129173.1 | 2084.76 | 1501.3 | 0.72 |
|  | ILMN_1793474 | INSIG1 | NM_198336.1 | 2782.82 | 2000.76 | 0.72 |
|  | ILMN_1799642 | TRIM24 | NM_015905.2 | 555.51 | 399.02 | 0.72 |
|  | ILMN_1815882 | HNRNPA1 | NM_031157.2 | 378.81 | 272.73 | 0.72 |
|  | ILMN_2046003 | ZDHHC6 | NM_022494.1 | 1326.43 | 952.56 | 0.72 |
|  | ILMN_2362545 | ZWINT | NM_001005413.1 | 493.08 | 354.42 | 0.72 |
|  | ILMN_2367681 | SPAG1 | NM_003114.3 | 413.48 | 297.55 | 0.72 |
|  | ILMN_3199647 | LOC645251 | XR_037203.1 | 1188.29 | 855.72 | 0.72 |
|  | ILMN_3248773 | C7orf40 | NR_003697.1 | 1183.35 | 853.35 | 0.72 |
|  | ILMN_3265797 | LOC100130561 | XM_001723189.1 | 3803.2 | 2738.81 | 0.72 |
|  | ILMN_1658407 | SLC43A3 | NM_014096.2 | 844.72 | 611.84 | 0.72 |
|  | ILMN_1671800 | LOC643911 | XM_931911.2 | 263.14 | 190.77 | 0.72 |
|  | ILMN_1692168 | UBE2Z | NM_023079.3 | 880.8 | 638.41 | 0.72 |
|  | ILMN_1710962 | TMEM97 | NM_014573.2 | 2104.12 | 1519.45 | 0.72 |
|  | ILMN_1711005 | CDC25A | NM_001789.2 | 626.14 | 452.47 | 0.72 |
|  | ILMN_1722066 | ARMC1 | NM_018120.3 | 1465.32 | 1058.89 | 0.72 |
|  | ILMN_1729051 | MSH6 | NM_000179.1 | 2615.73 | 1891.96 | 0.72 |
|  | ILMN_1731184 | MELK | NM_014791.2 | 1050.12 | 759.37 | 0.72 |
|  | ILMN_1766247 | ZBTB2 | NM_020861.1 | 389.19 | 281.63 | 0.72 |

|  | Probe ID | Symbol | Genebank accession | siControl | siSIRT6 | Fold ratio (siSIRT6/siControl) |
| --- | --- | --- | --- | --- | --- | --- |
|  | ILMN_1769409 | C9orf123 | NM_033428.1 | 266.07 | 192.58 | 0.72 |
|  | ILMN_1780153 | QTRT1 | NM_031209.1 | 339.91 | 246.77 | 0.73 |
|  | ILMN_1815169 | MCM5 | NM_006739.3 | 1285.1 | 929.51 | 0.72 |
|  | ILMN_2059689 | TMEM54 | NM_033504.2 | 403.19 | 291.36 | 0.72 |
|  | ILMN_2134974 | RAB38 | NM_022337.1 | 785.25 | 569.55 | 0.73 |
|  | ILMN_2219712 | HMGB2 | NM_002129.2 | 6067.98 | 4399.12 | 0.72 |
|  | ILMN_2232520 | ANKRD28 | NM_015199.2 | 333.45 | 242.25 | 0.73 |
|  | ILMN_2351466 | NTM | NM_001048209.1 | 344.18 | 249.06 | 0.72 |
|  | ILMN_2407124 | MCM8 | NM_032485.4 | 319.65 | 231.9 | 0.73 |
|  | ILMN_3246783 | LOC100131330 | XM_001714119.1 | 413.96 | 300.45 | 0.73 |
|  | ILMN_1655642 | FANCI | NM_018193.2 | 980.25 | 714.44 | 0.73 |
|  | ILMN_1666208 | C14orf106 | NM_018353.3 | 965.41 | 706.89 | 0.73 |
|  | ILMN_1683212 | SLC25A32 | NM_030780.2 | 377.33 | 275.71 | 0.73 |
|  | ILMN_1708858 | CSNK1E | NM_152221.2 | 781.08 | 571.79 | 0.73 |
|  | ILMN_1711862 | RNF7 | NM_183237.1 | 5121.44 | 3748.25 | 0.73 |
|  | ILMN_1712452 | KIF20B | NM_016195.2 | 1198.49 | 874.51 | 0.73 |
|  | ILMN_1718852 | PLCL1 | NM_006226.1 | 276.79 | 201.78 | 0.73 |
|  | ILMN_1723846 | FAM119B | NM_015433.2 | 739.29 | 539.07 | 0.73 |
|  | ILMN_1731070 | ORC6L | NM_014321.2 | 653.03 | 477.49 | 0.73 |
|  | ILMN_1748894 | GTPBP3 | NM_032620.1 | 894.13 | 651.22 | 0.73 |
|  | ILMN_1759154 | PABPN1 | NM_004643.1 | 1482.35 | 1081.14 | 0.73 |
|  | ILMN_1759973 | NDUFA5 | NM_005000.2 | 437.76 | 319.42 | 0.73 |
|  | ILMN_1765019 | SACM1L | NM_014016.2 | 1340.29 | 979.34 | 0.73 |
|  | ILMN_1768595 | DLG4 | NM_001365.2 | 241.07 | 176.07 | 0.73 |
|  | ILMN_1768816 | TMPO | NM_003276.1 | 354.01 | 259.15 | 0.73 |
|  | ILMN_1773200 | CP110 | NM_014711.3 | 962.07 | 702.65 | 0.73 |
|  | ILMN_1773865 | HSPA5 | NM_005347.2 | 1139.62 | 832.52 | 0.73 |
|  | ILMN_1781943 | FAM83D | NM_030919.2 | 1212.69 | 886.72 | 0.73 |
|  | ILMN_1783610 | HELLS | NM_018063.3 | 273.8 | 200.25 | 0.73 |
|  | ILMN_1793033 | RBM28 | NM_018077.1 | 552.95 | 404.78 | 0.73 |
|  | ILMN_1798533 | ZNF22 | NM_006963.3 | 2048.47 | 1491.62 | 0.73 |
|  | ILMN_2053281 | C14orf149 | NM_144581.1 | 1625.5 | 1186.37 | 0.73 |
|  | ILMN_2066756 | NCAPG2 | NM_017760.5 | 465.94 | 341.01 | 0.73 |
|  | ILMN_2147306 | PNRC2 | NM_017761.2 | 2016.54 | 1470.41 | 0.73 |
|  | ILMN_2307025 | CPNE1 | NM_152931.1 | 582.99 | 424.81 | 0.73 |
|  | ILMN_2344971 | FOXM1 | NM_021953.2 | 739.46 | 537.95 | 0.73 |
|  | ILMN_2364131 | TTPAL | NM_024331.3 | 383.92 | 280.01 | 0.73 |
|  | ILMN_3181420 | HMGXB4 | NM_005487.3 | 413.67 | 301.29 | 0.73 |
|  | ILMN_3214052 | LOC644877 | XR_038951.1 | 2784.11 | 2039.03 | 0.73 |
|  | ILMN_3238662 | SNORD89 | NR_003070.1 | 877.55 | 640.03 | 0.73 |
|  | ILMN_3239113 | LOC144438 | NR_024266.1 | 392.08 | 287.02 | 0.73 |
|  | ILMN_1658678 | SAAL1 | NM_138421.2 | 1394.95 | 1025.18 | 0.73 |
|  | ILMN_1671906 | MND1 | NM_032117.2 | 336.16 | 246.54 | 0.73 |
|  | ILMN_1680955 | AURKA | NM_198436.1 | 1808.19 | 1333.19 | 0.74 |
|  | ILMN_1686871 | PARP1 | NM_001618.2 | 3742.19 | 2754.04 | 0.74 |
|  | ILMN_1689001 | CDK4 | NM_000075.2 | 4295.67 | 3163.56 | 0.74 |

|  | Probe ID | Symbol | Genebank accession | siControl | siSIRT6 | Fold ratio (siSIRT6/siControl) |
| --- | --- | --- | --- | --- | --- | --- |
|  | ILMN_1696494 | CMTM6 | NM_017801.2 | 1658.5 | 1223.11 | 0.74 |
|  | ILMN_1697736 | EXOSC2 | NM_014285.4 | 629.33 | 462.08 | 0.73 |
|  | ILMN_1739645 | ANLN | NM_018685.2 | 1361.2 | 998.07 | 0.73 |
|  | ILMN_1761175 | RPS6KB2 | NM_003952.2 | 292.91 | 214.62 | 0.73 |
|  | ILMN_1762674 | NUP43 | NM_198887.1 | 263.87 | 193.43 | 0.73 |
|  | ILMN_1764629 | SLC39A14 | NM_015359.2 | 711.15 | 521.55 | 0.73 |
|  | ILMN_1781400 | SLC7A2 | NM_001008539.2 | 442.54 | 325.31 | 0.74 |
|  | ILMN_1795865 | FGFRL1 | NM_021923.3 | 598.55 | 439.48 | 0.73 |
|  | ILMN_1797307 | BUB1B | NM_001211.4 | 444.39 | 327.27 | 0.74 |
|  | ILMN_2043816 | ARPC5L | NM_030978.1 | 2468.92 | 1819.51 | 0.74 |
|  | ILMN_2061732 | YRDC | NM_024640.3 | 1790.32 | 1314.84 | 0.73 |
|  | ILMN_2175894 | HNRPR | NM_005826.2 | 2911.74 | 2148.83 | 0.74 |
|  | ILMN_2366330 | FERMT3 | NM_031471.4 | 1453.52 | 1072.18 | 0.74 |
|  | ILMN_2382083 | CUGBP1 | NM_198700.1 | 284.31 | 209 | 0.74 |
|  | ILMN_2405018 | PPP1CB | NM_206876.1 | 723.41 | 533.5 | 0.74 |
|  | ILMN_3192001 | CCL14 | NM_032962.2 | 222.91 | 164.2 | 0.74 |
|  | ILMN_3243471 | CNPY2 | NM_014255.4 | 2040.91 | 1498.53 | 0.73 |
|  | ILMN_3287952 | LOC100133800 | XR_039017.1 | 532.26 | 390.54 | 0.73 |
|  | ILMN_3306440 | TMEM194A | NM_001130963.1 | 300.45 | 220.4 | 0.73 |
|  | ILMN_1669113 | ATF5 | NM_012068.3 | 925.66 | 685.49 | 0.74 |
|  | ILMN_1671693 | C18orf10 | NM_015476.2 | 908.07 | 672.78 | 0.74 |
|  | ILMN_1678966 | SNRPF | NM_003095.1 | 6104.54 | 4507.15 | 0.74 |
|  | ILMN_1686626 | BAT1 | NM_080598.4 | 3968.38 | 2933.35 | 0.74 |
|  | ILMN_1690464 | TMEM20 | NM_153226.1 | 224.98 | 166.07 | 0.74 |
|  | ILMN_1695588 | HNRPC | NM_031314.1 | 3738.73 | 2774.48 | 0.74 |
|  | ILMN_1695658 | KIF20A | NM_005733.1 | 1473.13 | 1089.41 | 0.74 |
|  | ILMN_1696713 | POLA2 | NM_002689.2 | 979.79 | 727.77 | 0.74 |
|  | ILMN_1708081 | LCLAT1 | NM_001002257.1 | 922.88 | 682.02 | 0.74 |
|  | ILMN_1710207 | C10orf6 | NM_018121.2 | 406.56 | 302.26 | 0.74 |
|  | ILMN_1720114 | GMNN | NM_015895.3 | 2360.7 | 1743.37 | 0.74 |
|  | ILMN_1733390 | LARP1B | NM_032239.2 | 541.57 | 400.32 | 0.74 |
|  | ILMN_1754304 | SNRNP48 | NM_152551.3 | 205.69 | 152.43 | 0.74 |
|  | ILMN_1756701 | DHRS11 | NM_024308.3 | 386.41 | 286.49 | 0.74 |
|  | ILMN_1768097 | RPGR | NM_001023582.1 | 555.89 | 413 | 0.74 |
|  | ILMN_1776173 | PSMD7 | NM_002811.3 | 3475.49 | 2569.02 | 0.74 |
|  | ILMN_1790819 | LOC728556 | XM_001129672.1 | 754.48 | 559.5 | 0.74 |
|  | ILMN_1806106 | GNL3 | NM_014366.4 | 2330.35 | 1731.33 | 0.74 |
|  | ILMN_1903568 |  | AK022936 | 1458.23 | 1081.89 | 0.74 |
|  | ILMN_2041293 | SQLE | NM_003129.3 | 2939.46 | 2177.31 | 0.74 |
|  | ILMN_2112460 | MAD2L1 | NM_002358.2 | 1862.47 | 1382.12 | 0.74 |
|  | ILMN_2128358 | TPP2 | NM_003291.1 | 239.02 | 177.46 | 0.74 |
|  | ILMN_2148150 | CHAC2 | NM_001008708.1 | 474.96 | 351.49 | 0.74 |
|  | ILMN_2211800 | HMGB1L1 | NM_001008735.1 | 6372.6 | 4723.59 | 0.74 |
|  | ILMN_2277676 | ERCC1 | NM_001983.2 | 1080.89 | 803.23 | 0.74 |
|  | ILMN_2349393 | MDK | NM_001012334.1 | 1519.8 | 1128.87 | 0.74 |
|  | ILMN_2377430 | AGPAT2 | NM_001012727.1 | 509.88 | 377.76 | 0.74 |

|  | Probe ID | Symbol | Genebank accession | siControl | siSIRT6 | Fold ratio (siSIRT6/siControl) |
| --- | --- | --- | --- | --- | --- | --- |
|  | ILMN_2392546 | PAICS | NM_006452.3 | 4719.22 | 3502.09 | 0.74 |
|  | ILMN_3251482 | ALG10B | NM_001013620.3 | 212.21 | 157.55 | 0.74 |
|  | ILMN_1669584 | ILF3 | NM_004516.2 | 608.03 | 454.67 | 0.75 |
|  | ILMN_1688152 | IL27RA | NM_004843.2 | 262.65 | 196.54 | 0.75 |
|  | ILMN_1713751 | ADAM19 | NM_033274.2 | 382.24 | 286.09 | 0.75 |
|  | ILMN_1724990 | C3orf75 | NM_001031703.2 | 255.82 | 190.9 | 0.75 |
|  | ILMN_1735461 | DDX21 | NM_004728.2 | 4434.84 | 3299.43 | 0.74 |
|  | ILMN_1736816 | C13orf3 | NM_145061.3 | 505.65 | 377.41 | 0.75 |
|  | ILMN_1737965 | ELOVL4 | NM_022726.2 | 386.95 | 288.01 | 0.74 |
|  | ILMN_1742250 | CCNH | NM_001239.2 | 570.47 | 425.99 | 0.75 |
|  | ILMN_1746257 | DAZAP1 | NM_170711.1 | 927.8 | 694.58 | 0.75 |
|  | ILMN_1747016 | CEP55 | NM_018131.3 | 2614.53 | 1956.42 | 0.75 |
|  | ILMN_1753183 | CDCA4 | NM_017955.2 | 741.34 | 552.95 | 0.75 |
|  | ILMN_1763529 | LOC650254 | XM_499385.2 | 368.37 | 275.26 | 0.75 |
|  | ILMN_1769545 | PRPF19 | NM_014502.3 | 1019.75 | 759.37 | 0.74 |
|  | ILMN_1788701 | PSIP1 | NM_033222.2 | 1059.13 | 792.9 | 0.75 |
|  | ILMN_1790562 | EYA3 | NM_001990.2 | 203.61 | 152.04 | 0.75 |
|  | ILMN_1792681 | CCDC86 | NM_024098.1 | 2893.63 | 2160.28 | 0.75 |
|  | ILMN_1796417 | ASNS | NM_133436.1 | 775.33 | 579.37 | 0.75 |
|  | ILMN_1804248 | FDPS | NM_002004.2 | 2030.57 | 1510.35 | 0.74 |
|  | ILMN_1814122 | MDC1 | NM_014641.1 | 788.52 | 587.32 | 0.74 |
|  | ILMN_2043452 | FANCE | NM_021922.2 | 540.69 | 402.08 | 0.74 |
|  | ILMN_2087646 | HLX | NM_021958.2 | 320.61 | 240.02 | 0.75 |
|  | ILMN_2094061 | IMPA2 | NM_014214.1 | 206.74 | 154.31 | 0.75 |
|  | ILMN_2131336 | TMEM194 | NM_015257.1 | 276.47 | 206.88 | 0.75 |
|  | ILMN_2179778 | PHLDB2 | NM_145753.1 | 1068.47 | 795.29 | 0.74 |
|  | ILMN_2180371 | C12orf24 | NM_013300.1 | 1058.89 | 789.61 | 0.75 |
|  | ILMN_2376133 | KIAA1191 | NM_020444.3 | 1624.75 | 1216.34 | 0.75 |
|  | ILMN_2398107 | ASNS | NM_133436.1 | 534.11 | 397.18 | 0.74 |
|  | ILMN_3188124 | LOC100130511 | XR_038018.1 | 1967.75 | 1468.71 | 0.75 |
|  | ILMN_3260345 | AGFG1 | NM_001135189.1 | 404.31 | 301.22 | 0.75 |
|  | ILMN_3294365 | LOC646993 | XM_001717725.1 | 923.31 | 687.87 | 0.75 |
|  | ILMN_1654246 | SIRT6 | NM_016539.1 | 167.15 | 125.22 | 0.75 |
|  | ILMN_1669252 | CUL2 | NM_003591.2 | 1464.64 | 1103.85 | 0.75 |
|  | ILMN_1678805 | POMT2 | NM_013382.3 | 247.85 | 185.98 | 0.75 |
|  | ILMN_1683441 | NCAPD3 | NM_015261.2 | 277.82 | 208.66 | 0.75 |
|  | ILMN_1693108 | RUVBL1 | NM_003707.1 | 1073.17 | 804.71 | 0.75 |
|  | ILMN_1708059 | USP13 | NM_003940.1 | 587.45 | 440.7 | 0.75 |
|  | ILMN_1709484 | BLM | NM_000057.2 | 305.35 | 229.02 | 0.75 |
|  | ILMN_1715401 | MT1G | NM_005950.1 | 5170.18 | 3892.1 | 0.75 |
|  | ILMN_1717490 | RPL6 | NM_001024662.1 | 1282.14 | 966.75 | 0.75 |
|  | ILMN_1719204 | PRPF31 | NM_015629.2 | 1902.48 | 1429.54 | 0.75 |
|  | ILMN_1726756 | FANCB | NM_001018113.1 | 287.15 | 215.22 | 0.75 |
|  | ILMN_1728934 | PRC1 | NM_199413.1 | 2909.05 | 2189.93 | 0.75 |
|  | ILMN_1735093 | TIMELESS | NM_003920.2 | 717.58 | 538.45 | 0.75 |
|  | ILMN_1738027 | BRCA1 | NM_007299.2 | 525.54 | 394.35 | 0.75 |

|  | Probe ID | Symbol | Genebank accession | siControl | siSIRT6 | Fold ratio (siSIRT6/siControl) |
| --- | --- | --- | --- | --- | --- | --- |
|  | ILMN_1738530 | ATAD3A | NM_018188.2 | 909.75 | 685.81 | 0.75 |
|  | ILMN_1741997 | SNRPC | NM_003093.1 | 1220.85 | 920.11 | 0.75 |
|  | ILMN_1742779 | CENPL | NM_033319.1 | 535.47 | 402.73 | 0.75 |
|  | ILMN_1746633 | C9orf25 | NM_147202.1 | 252.01 | 190.19 | 0.75 |
|  | ILMN_1746917 | LOC729843 | XR_016056.1 | 613.96 | 462.61 | 0.75 |
|  | ILMN_1748916 | C18orf55 | NM_014177.1 | 1472.79 | 1111.02 | 0.75 |
|  | ILMN_1755862 | PFAS | NM_012393.1 | 1532.5 | 1148.07 | 0.75 |
|  | ILMN_1758823 | SMARCB1 | NM_001007468.1 | 1285.99 | 968.76 | 0.75 |
|  | ILMN_1770244 | CBX1 | NM_006807.3 | 907.03 | 680.76 | 0.75 |
|  | ILMN_1777342 | PREX1 | NM_020820.3 | 341.4 | 255.94 | 0.75 |
|  | ILMN_1778226 | EXTL3 | NM_001440.2 | 461.87 | 346.81 | 0.75 |
|  | ILMN_1791119 | NDUFA10 | NM_004544.2 | 1258.95 | 944.02 | 0.75 |
|  | ILMN_1792947 | ESCO2 | NM_001017420.2 | 317.88 | 239.24 | 0.75 |
|  | ILMN_1796099 | LOC644380 | XM_929628.1 | 1938.42 | 1457.89 | 0.75 |
|  | ILMN_1801257 | CENPA | NM_001042426.1 | 842.97 | 633.41 | 0.75 |
|  | ILMN_1805842 | FHL1 | NM_001449.3 | 426.18 | 321.42 | 0.75 |
|  | ILMN_1807649 | SPOPL | NM_001001664.1 | 319.42 | 239.52 | 0.75 |
|  | ILMN_1807945 | ANP32A | NM_006305.2 | 707.54 | 533.87 | 0.75 |
|  | ILMN_1815134 | PI4K2B | NM_018323.2 | 729.62 | 547.36 | 0.75 |
|  | ILMN_2092693 | LSM12 | NM_152344.1 | 695.55 | 523.85 | 0.75 |
|  | ILMN_2109156 | RANBP1 | NM_002882.2 | 8983.11 | 6742.18 | 0.75 |
|  | ILMN_2112402 | PHF5A | NM_032758.3 | 1788.25 | 1349.3 | 0.75 |
|  | ILMN_2151488 | RMI1 | NM_024945.1 | 844.92 | 635.46 | 0.75 |
|  | ILMN_2311089 | BRCA1 | NM_007304.2 | 410.91 | 307.9 | 0.75 |
|  | ILMN_3205271 | LOC100132863 | XR_036905.1 | 14279.59 | 10722.35 | 0.75 |
|  | ILMN_3220861 | LOC729952 | XR_015724.2 | 226.23 | 170.11 | 0.75 |
|  | ILMN_3227023 | SNHG7 | NR_003672.2 | 2055.58 | 1542.8 | 0.75 |
|  | ILMN_3230241 | LOC728975 | XR_042006.1 | 202.77 | 152.29 | 0.75 |
|  | ILMN_3251383 | CCDC74B | NM_207310.1 | 215.27 | 161.76 | 0.75 |
|  | ILMN_3263702 | LOC100128881 | XM_001717756.1 | 255.47 | 191.47 | 0.75 |
|  | ILMN_3269405 | HNRNPM | NM_005968.3 | 5887.07 | 4443.05 | 0.75 |
|  | ILMN_3281502 | LOC653375 | XM_001721571.1 | 411 | 308.62 | 0.75 |
|  | ILMN_1651719 | MBTPS1 | NM_003791.2 | 1447.49 | 1099.27 | 0.76 |
|  | ILMN_1653342 | DUS1L | NM_022156.3 | 280.85 | 212.21 | 0.76 |
|  | ILMN_1665423 | ZFP91 | NM_053023.3 | 3106.34 | 2361.79 | 0.76 |
|  | ILMN_1672742 | ARHGAP23 | XM_290799.6 | 200.44 | 151.38 | 0.76 |
|  | ILMN_1677239 | CCDC14 | NM_022757.3 | 942.05 | 716.09 | 0.76 |
|  | ILMN_1677768 | POR | NM_000941.2 | 316.12 | 239.35 | 0.76 |
|  | ILMN_1686516 | CUGBP1 | NM_001025596.1 | 298.86 | 226.18 | 0.76 |
|  | ILMN_1688464 | MAP6D1 | NM_024871.1 | 367.35 | 279.3 | 0.76 |
|  | ILMN_1706238 | CSE1L | NM_001316.2 | 3910.13 | 2955.8 | 0.76 |
|  | ILMN_1709162 | SHCBP1 | NM_024745.3 | 588.27 | 445.82 | 0.76 |
|  | ILMN_1718988 | DAZAP2 | NM_014764.2 | 5059.11 | 3821.7 | 0.76 |
|  | ILMN_1719627 | SLC27A3 | NM_024330.1 | 990.26 | 748.75 | 0.76 |
|  | ILMN_1724493 | LYSMD2 | NM_153374.1 | 927.37 | 701.84 | 0.76 |
|  | ILMN_1739558 | CRELD1 | NM_001031717.2 | 519.03 | 392.62 | 0.76 |

|  | Probe ID | Symbol | Genebank accession | siControl | siSIRT6 | Fold ratio (siSIRT6/siControl) |
| --- | --- | --- | --- | --- | --- | --- |
|  | ILMN_1751871 | STK11 | NM_000455.4 | 197.18 | 149.09 | 0.76 |
|  | ILMN_1752968 | LAMB2 | NM_002292.3 | 502.16 | 381.01 | 0.76 |
|  | ILMN_1755075 | IDI1 | NM_004508.2 | 1223.11 | 929.09 | 0.76 |
|  | ILMN_1761939 | TIPIN | NM_017858.1 | 613.25 | 464.76 | 0.76 |
|  | ILMN_1763409 | LRRC8D | NM_018103.3 | 282.22 | 214.18 | 0.76 |
|  | ILMN_1771039 | GTSE1 | NM_016426.4 | 481.26 | 363.97 | 0.76 |
|  | ILMN_1786024 | POLR3H | NM_001018052.1 | 473.65 | 359.79 | 0.76 |
|  | ILMN_1788462 | AMD1 | NM_001033059.1 | 2744.51 | 2085.24 | 0.76 |
|  | ILMN_1793360 | APITD1 | NM_199295.1 | 630.93 | 477.71 | 0.76 |
|  | ILMN_1804953 | CCDC18 | NM_206886.2 | 208.47 | 158.17 | 0.76 |
|  | ILMN_1808071 | KIF14 | NM_014875.1 | 561.7 | 424.71 | 0.76 |
|  | ILMN_2101920 | HNRPH1 | NM_005520.1 | 1430.53 | 1080.64 | 0.76 |
|  | ILMN_2150894 | ALDH1B1 | NM_000692.3 | 1285.99 | 972.8 | 0.76 |
|  | ILMN_2219131 | RPS15 | NM_001018.3 | 1198.49 | 906.4 | 0.76 |
|  | ILMN_2355033 | KIAA1147 | NM_001080392.1 | 639.44 | 483.15 | 0.76 |
|  | ILMN_2374425 | CCNE1 | NM_001238.1 | 379.69 | 286.75 | 0.76 |
|  | ILMN_2376502 | RHOBTB1 | NM_198225.1 | 246.6 | 187.1 | 0.76 |
|  | ILMN_3201658 | LOC642585 | XR_038667.1 | 765.19 | 578.83 | 0.76 |
|  | ILMN_3263329 | GAR1 | NM_018983.3 | 2873.65 | 2172.29 | 0.76 |
|  | ILMN_3265895 | HNRNPR | NM_001102398.1 | 305.78 | 231.25 | 0.76 |
|  | ILMN_3266471 | LOC100129566 | XM_001718519.1 | 990.49 | 750.13 | 0.76 |
|  | ILMN_3268165 | LOC100128353 | XR_038849.1 | 1874.99 | 1419.34 | 0.76 |
|  | ILMN_3276697 | LOC646956 | XR_017376.2 | 364.56 | 275.45 | 0.76 |
|  | ILMN_3293159 | LOC100132767 | XM_001725561.1 | 227.81 | 173.04 | 0.76 |
|  | ILMN_3298829 | LOC729505 | XM_001131805.2 | 1118.75 | 845.7 | 0.76 |
|  | ILMN_1652008 | C15orf23 | NM_033286.1 | 477.6 | 365.65 | 0.77 |
|  | ILMN_1652638 | LRRC58 | XM_938862.2 | 413.76 | 314.95 | 0.76 |
|  | ILMN_1658847 | MGC61598 | XM_939432.1 | 803.41 | 615.52 | 0.77 |
|  | ILMN_1664682 | DNA2 | NM_001080449.1 | 220.5 | 167.96 | 0.76 |
|  | ILMN_1666384 | LOC151579 | XM_045290.6 | 5183.34 | 3965.63 | 0.77 |
|  | ILMN_1667213 | DFFA | NM_213566.1 | 611.69 | 468.64 | 0.77 |
|  | ILMN_1671257 | DKC1 | NM_001363.2 | 4322.55 | 3304.77 | 0.76 |
|  | ILMN_1676448 | WDFY1 | NM_020830.3 | 1958.23 | 1496.8 | 0.76 |
|  | ILMN_1677719 | CHST1 | NM_003654.3 | 271.85 | 207.94 | 0.76 |
|  | ILMN_1679177 | MARS2 | NM_138395.2 | 390.27 | 298.52 | 0.76 |
|  | ILMN_1679800 | BRIX1 | NM_018321.3 | 4064.89 | 3098.45 | 0.76 |
|  | ILMN_1682694 | LOC203547 | NM_001017980.2 | 2084.76 | 1589.11 | 0.76 |
|  | ILMN_1686097 | TOP2A | NM_001067.2 | 2133 | 1623.62 | 0.76 |
|  | ILMN_1693227 | ZC3H7A | NM_014153.2 | 799.34 | 611.98 | 0.77 |
|  | ILMN_1697703 | HPDL | NM_032756.2 | 196.13 | 150.23 | 0.77 |
|  | ILMN_1720526 | CENPN | NM_018455.3 | 1612.78 | 1227.92 | 0.76 |
|  | ILMN_1723021 | ICMT | NM_012405.3 | 1060.11 | 810.69 | 0.76 |
|  | ILMN_1730698 | ODF2 | NM_002540.3 | 648.07 | 495.25 | 0.76 |
|  | ILMN_1737413 | MSH2 | NM_000251.1 | 575.76 | 439.99 | 0.76 |
|  | ILMN_1742147 | UBL4A | NM_014235.3 | 500.65 | 381.45 | 0.76 |
|  | ILMN_1756043 | WDHD1 | NM_007086.2 | 245.46 | 187.32 | 0.76 |

|  | Probe ID | Symbol | Genebank accession | siControl | siSIRT6 | Fold ratio (siSIRT6/siControl) |
| --- | --- | --- | --- | --- | --- | --- |
|  | ILMN_1763359 | PEG10 | XM_499343.2 | 217.02 | 165.23 | 0.76 |
|  | ILMN_1765085 | TRMT6 | NM_015939.3 | 383.04 | 292.04 | 0.76 |
|  | ILMN_1773968 | SERBP1 | NM_001018069.1 | 973.7 | 743.4 | 0.76 |
|  | ILMN_1774091 | FECH | NM_000140.2 | 318.61 | 243.42 | 0.76 |
|  | ILMN_1774733 | SOCS1 | NM_003745.1 | 197.08 | 150.61 | 0.76 |
|  | ILMN_1776325 | UBE2Q1 | NM_017582.5 | 533.87 | 407.5 | 0.76 |
|  | ILMN_1778890 | PPIL5 | NM_152329.3 | 581.24 | 443.46 | 0.76 |
|  | ILMN_1781251 | LOC653115 | XM_926415.1 | 471.68 | 359.95 | 0.76 |
|  | ILMN_1786050 | RBBP9 | NM_006606.2 | 377.59 | 287.42 | 0.76 |
|  | ILMN_1793651 | UBE2N | NM_003348.3 | 2155.79 | 1648.95 | 0.76 |
|  | ILMN_1867663 |  | AF086087 | 258.32 | 197.49 | 0.76 |
|  | ILMN_2104106 | XPR1 | NM_004736.2 | 1547.44 | 1177.9 | 0.76 |
|  | ILMN_2123402 | TMEM4 | NM_014255.3 | 1603.12 | 1223.39 | 0.76 |
|  | ILMN_2169089 | C18orf54 | NM_173529.3 | 623.68 | 477.16 | 0.77 |
|  | ILMN_2204545 | ST3GAL4 | NM_006278.1 | 217.37 | 165.34 | 0.76 |
|  | ILMN_2229242 | LSM3 | NM_014463.1 | 4032.15 | 3082.03 | 0.76 |
|  | ILMN_2366334 | FERMT3 | NM_031471.4 | 431.34 | 329.16 | 0.76 |
|  | ILMN_2405521 | MTHFD2 | NM_001040409.1 | 1489.9 | 1140.67 | 0.77 |
|  | ILMN_3228822 | TMEM194A | NM_015257.2 | 369.3 | 282.54 | 0.77 |
|  | ILMN_3233135 | FAM178A | NM_018121.3 | 286.82 | 219.13 | 0.76 |
|  | ILMN_3236877 | LOC729362 | XR_041521.1 | 627.73 | 477.38 | 0.76 |
|  | ILMN_3246401 | AIF1L | NM_031426.2 | 440.4 | 337.4 | 0.77 |
|  | ILMN_3251232 | HMGN2 | NM_005517.3 | 282.94 | 216.27 | 0.76 |
|  | ILMN_3251526 | USP13 | NM_003940.2 | 374.72 | 286.42 | 0.76 |
|  | ILMN_3300972 | SIVA1 | NM_021709.2 | 3465.06 | 2646.74 | 0.76 |
|  | ILMN_1652237 | CBR3 | NM_001236.3 | 537.83 | 413.57 | 0.77 |
|  | ILMN_1656501 | DUSP5 | NM_004419.3 | 1337.2 | 1030.88 | 0.77 |
|  | ILMN_1658143 | RFC3 | NM_002915.3 | 345.45 | 264.84 | 0.77 |
|  | ILMN_1658290 | C16orf68 | NM_024109.2 | 658.48 | 508.23 | 0.77 |
|  | ILMN_1661776 | CENPJ | NM_018451.3 | 467.88 | 359.79 | 0.77 |
|  | ILMN_1664641 | MED4 | NM_014166.2 | 678.56 | 521.31 | 0.77 |
|  | ILMN_1669572 | RNF126 | NM_194460.1 | 363.8 | 279.95 | 0.77 |
|  | ILMN_1695357 | CCDC99 | NM_017785.3 | 1227.07 | 940.31 | 0.77 |
|  | ILMN_1696485 | HNRNPAB | NM_031266.2 | 1652.38 | 1266.24 | 0.77 |
|  | ILMN_1700413 | MAFF | NM_012323.2 | 204.98 | 157.55 | 0.77 |
|  | ILMN_1701032 | TFPI | NM_001032281.2 | 631.07 | 485.61 | 0.77 |
|  | ILMN_1706531 | ABCC5 | NM_005688.2 | 242.64 | 185.98 | 0.77 |
|  | ILMN_1707156 | LRRFIP2 | NM_017724.1 | 1481.32 | 1135.94 | 0.77 |
|  | ILMN_1714730 | UBE2C | NM_181803.1 | 3737 | 2868.34 | 0.77 |
|  | ILMN_1715416 | NUP188 | NM_015354.1 | 391.99 | 301.43 | 0.77 |
|  | ILMN_1716400 | FOXM1 | NM_202003.1 | 378.37 | 290.49 | 0.77 |
|  | ILMN_1738854 | CACHD1 | NM_020925.2 | 256.24 | 196.81 | 0.77 |
|  | ILMN_1745256 | CXXC5 | NM_016463.5 | 256.18 | 196.9 | 0.77 |
|  | ILMN_1753885 | YTHDF1 | NM_017798.2 | 1341.53 | 1033.27 | 0.77 |
|  | ILMN_1758915 | PDCD2 | NM_144781.1 | 594.01 | 455.19 | 0.77 |
|  | ILMN_1767324 | EIF4EBP1 | NM_004095.3 | 528.47 | 406.28 | 0.77 |

|  | Probe ID | Symbol | Genebank accession | siControl | siSIRT6 | Fold ratio (siSIRT6/siControl) |
| --- | --- | --- | --- | --- | --- | --- |
|  | ILMN_1768020 | HAUS8 | NM_033417.1 | 311.41 | 239.46 | 0.77 |
|  | ILMN_1768127 | EBNA1BP2 | NM_006824.1 | 7562.6 | 5835.61 | 0.77 |
|  | ILMN_1790537 | C16orf75 | NM_152308.1 | 395.63 | 304.44 | 0.77 |
|  | ILMN_1695945 | MEIS2 | NM_172315.1 | 392.99 | 512.83 | 1.30 |
|  | ILMN_1697962 | NSMCE1 | NM_145080.3 | 552.05 | 721.41 | 1.31 |
|  | ILMN_1703111 | BBS7 | NM_018190.2 | 272.42 | 353.03 | 1.30 |
|  | ILMN_1704554 | LOC648470 | XM_937514.1 | 185.72 | 241.24 | 1.30 |
|  | ILMN_1707084 | UBE2D4 | NM_015983.2 | 688.19 | 892.68 | 1.30 |
|  | ILMN_1707804 | SULT1A3 | NM_003166.3 | 174.93 | 226.7 | 1.30 |
|  | ILMN_1710523 | ATP8B1 | NM_005603.3 | 201.23 | 260.96 | 1.30 |
|  | ILMN_1712975 | YIF1A | NM_020470.1 | 1996.14 | 2600.67 | 1.30 |
|  | ILMN_1721741 | ATPBD1B | NM_018066.2 | 484.94 | 631.51 | 1.30 |
|  | ILMN_1722820 | KDELR3 | NM_006855.2 | 891.86 | 1158.73 | 1.30 |
|  | ILMN_1725130 | FAM50A | NM_004699.1 | 4117.82 | 5341.39 | 1.30 |
|  | ILMN_1728478 | CXCL16 | NM_022059.1 | 252.83 | 327.72 | 1.30 |
|  | ILMN_1729288 | C1QTNF6 | NM_031910.3 | 389.28 | 507.41 | 1.30 |
|  | ILMN_1730612 | DBNDD2 | NM_001048223.1 | 1679.71 | 2173.29 | 1.29 |
|  | ILMN_1732039 | DDX3Y | NM_004660.2 | 500.07 | 650.92 | 1.30 |
|  | ILMN_1733248 | NRBP2 | NM_178564.2 | 262.47 | 342.91 | 1.31 |
|  | ILMN_1739497 | GTF2H5 | NM_207118.1 | 2000.76 | 2604.28 | 1.30 |
|  | ILMN_1745152 | UQCC | NM_018244.3 | 971 | 1254.59 | 1.29 |
|  | ILMN_1745471 | IRF9 | NM_006084.4 | 398.66 | 520.11 | 1.30 |
|  | ILMN_1754149 | LETMD1 | NM_001024668.1 | 249.58 | 322.84 | 1.29 |
|  | ILMN_1754988 | N6AMT1 | NM_013240.3 | 203.61 | 263.87 | 1.30 |
|  | ILMN_1756862 | APOL3 | NM_145641.1 | 175.3 | 228.18 | 1.30 |
|  | ILMN_1762115 | CRYZL1 | NM_145858.2 | 530.42 | 687.24 | 1.30 |
|  | ILMN_1773073 | PHYH | NM_001037537.1 | 544.2 | 705.09 | 1.30 |
|  | ILMN_1779147 | ENC1 | NM_003633.1 | 667.67 | 872.29 | 1.31 |
|  | ILMN_1786469 | FBXO22 | NM_012170.2 | 667.21 | 865.07 | 1.30 |
|  | ILMN_1809141 | ING4 | NM_016162.2 | 173.81 | 225.35 | 1.30 |
|  | ILMN_1809695 | CAMK2G | NM_001222.2 | 379.07 | 489.44 | 1.29 |
|  | ILMN_1810289 | FER1L3 | NM_133337.1 | 1989.24 | 2568.42 | 1.29 |
|  | ILMN_1810910 | CFH | NM_001014975.1 | 172.37 | 224.15 | 1.30 |
|  | ILMN_2049766 | NFE2L3 | NM_004289.5 | 1962.76 | 2553.63 | 1.30 |
|  | ILMN_2073289 | MTSS1 | NM_014751.2 | 716.42 | 931.66 | 1.30 |
|  | ILMN_2143795 | MGC4677 | NM_052871.3 | 4242.41 | 5505.53 | 1.30 |
|  | ILMN_2151441 | FAM103A1 | NM_031452.2 | 781.44 | 1020.46 | 1.31 |
|  | ILMN_2191568 | TUSC4 | NM_006545.4 | 681.07 | 883.45 | 1.30 |
|  | ILMN_2203463 | SPATS2 | NM_023071.1 | 1003.39 | 1300.03 | 1.30 |
|  | ILMN_2229032 | NME6 | NM_005793.3 | 390.54 | 506.47 | 1.30 |
|  | ILMN_2274199 | SUPT3H | NM_181356.1 | 143.11 | 186.63 | 1.30 |
|  | ILMN_2294751 | ASCC3 | NM_006828.2 | 1013.88 | 1316.66 | 1.30 |
|  | ILMN_2308582 | CYB5R3 | NM_007326.2 | 8171.21 | 10572.29 | 1.29 |
|  | ILMN_2312709 | LCMT1 | NM_016309.2 | 1108.2 | 1434.5 | 1.29 |
|  | ILMN_2334210 | ITGB4 | NM_001005619.1 | 115.39 | 149.98 | 1.30 |
|  | ILMN_2341815 | TFG | NM_006070.4 | 4605.05 | 5996.89 | 1.30 |

|  | Probe ID | Symbol | Genebank accession | siControl | siSIRT6 | Fold ratio (siSIRT6/siControl) |
| --- | --- | --- | --- | --- | --- | --- |
|  | ILMN_2376258 | SMARCA1 | NM_003069.2 | 242.19 | 316.12 | 1.31 |
|  | ILMN_2394250 | PLEKHA1 | NM_021622.3 | 1447.49 | 1868.07 | 1.29 |
|  | ILMN_3190833 | CCRL2 | NM_001130910.1 | 221.53 | 286.69 | 1.29 |
|  | ILMN_3236858 | NYNRIN | NM_025081.2 | 308.97 | 403.29 | 1.31 |
|  | ILMN_3241234 | LOC730278 | XM_001126471.1 | 7256.21 | 9410.14 | 1.30 |
|  | ILMN_3249032 | EPCAM | NM_002354.2 | 146.39 | 190.86 | 1.30 |
|  | ILMN_3250899 | SULT1A3 | NM_177552.2 | 153.03 | 198 | 1.29 |
|  | ILMN_1652371 | KIAA1324L | NM_152748.2 | 217.37 | 285.17 | 1.31 |
|  | ILMN_1652445 | RAC1 | NM_018890.2 | 196.72 | 258.97 | 1.32 |
|  | ILMN_1653028 | COL4A1 | NM_001845.4 | 3821.7 | 5003.31 | 1.31 |
|  | ILMN_1654112 | PARD6A | NM_001037281.1 | 148.36 | 195.32 | 1.32 |
|  | ILMN_1654629 | TMEM175 | NM_032326.2 | 289.01 | 378.2 | 1.31 |
|  | ILMN_1656910 | TRIM6 | NM_058166.3 | 185.34 | 242.92 | 1.31 |
|  | ILMN_1657746 | BPHL | NM_004332.1 | 250.15 | 328.71 | 1.31 |
|  | ILMN_1661484 | ZBTB45 | NM_032792.2 | 363.46 | 477.82 | 1.31 |
|  | ILMN_1665510 | ERRFI1 | NM_018948.2 | 1240.18 | 1622.5 | 1.31 |
|  | ILMN_1669362 | IGFBP6 | NM_002178.2 | 330.08 | 435.84 | 1.32 |
|  | ILMN_1673566 | ADAMTS1 | NM_006988.3 | 580.57 | 763.42 | 1.31 |
|  | ILMN_1678454 | CASP4 | NM_001225.3 | 2041.39 | 2703.6 | 1.32 |
|  | ILMN_1680313 | STX4 | NM_004604.3 | 628.89 | 824.86 | 1.31 |
|  | ILMN_1682165 | NT5C2 | NM_012229.2 | 1135.41 | 1496.8 | 1.32 |
|  | ILMN_1684210 | NPAL3 | NM_020448.3 | 184.99 | 243.42 | 1.32 |
|  | ILMN_1693334 | P4HA1 | NM_000917.2 | 1081.39 | 1414.1 | 1.31 |
|  | ILMN_1694219 | ARIH1 | NM_005744.2 | 368.62 | 484.49 | 1.31 |
|  | ILMN_1694799 | PIAS2 | NM_004671.2 | 432.83 | 566.26 | 1.31 |
|  | ILMN_1695290 | FERMT2 | NM_006832.1 | 3343.17 | 4395.06 | 1.31 |
|  | ILMN_1695959 | C21orf63 | NM_058187.3 | 907.65 | 1200.71 | 1.32 |
|  | ILMN_1699636 | ACIN1 | NM_014977.2 | 608.03 | 796.58 | 1.31 |
|  | ILMN_1701487 | TAOK2 | NM_016151.2 | 325.53 | 430.84 | 1.32 |
|  | ILMN_1701875 | ZYX | NM_003461.4 | 5812.73 | 7632.82 | 1.31 |
|  | ILMN_1709809 | NHP2L1 | NM_005008.2 | 983.19 | 1285.4 | 1.31 |
|  | ILMN_1715569 | CCDC53 | NM_016053.2 | 982.29 | 1286.88 | 1.31 |
|  | ILMN_1716026 | ARMCX6 | NM_019007.3 | 802.67 | 1056.69 | 1.32 |
|  | ILMN_1717099 | DSCR3 | NM_006052.1 | 679.82 | 899.3 | 1.32 |
|  | ILMN_1727574 | ZNF827 | NM_178835.3 | 274.56 | 359.45 | 1.31 |
|  | ILMN_1729117 | COL5A2 | NM_000393.3 | 1678.16 | 2213.84 | 1.32 |
|  | ILMN_1730007 | MPZL2 | NM_144765.1 | 148.78 | 196.31 | 1.32 |
|  | ILMN_1739001 | TACSTD2 | NM_002353.1 | 2178.32 | 2878.96 | 1.32 |
|  | ILMN_1744268 | PLEC1 | NM_000445.2 | 384.9 | 505.07 | 1.31 |
|  | ILMN_1746029 | SPATS2 | NM_023071.3 | 708.36 | 934.9 | 1.32 |
|  | ILMN_1746175 | TNFSF4 | NM_003326.2 | 462.72 | 610.56 | 1.32 |
|  | ILMN_1747968 | RBM33 | NM_001008408.3 | 202.39 | 265.4 | 1.31 |
|  | ILMN_1750273 | RPL23AP7 | NR_000029.2 | 415.01 | 547.48 | 1.32 |
|  | ILMN_1755658 | ABI3 | NM_016428.2 | 965.41 | 1273.57 | 1.32 |
|  | ILMN_1769883 | IDE | NM_004969.1 | 397.46 | 523.24 | 1.32 |
|  | ILMN_1770822 | RPS6KA3 | XM_944112.1 | 245.34 | 324.48 | 1.32 |

|  | Probe ID | Symbol | Genebank accession | siControl | siSIRT6 | Fold ratio (siSIRT6/siControl) |
| --- | --- | --- | --- | --- | --- | --- |
|  | ILMN_1778371 | CCBL2 | NM_001008661.1 | 717.58 | 944.45 | 1.32 |
|  | ILMN_1779558 | GAS6 | NM_000820.1 | 398.01 | 525.06 | 1.32 |
|  | ILMN_1784749 | GAS6 | NM_000820.1 | 363.72 | 477.82 | 1.31 |
|  | ILMN_1787308 | PIP4K2C | NM_024779.3 | 185.21 | 242.19 | 1.31 |
|  | ILMN_1789702 | GBE1 | NM_000158.2 | 1882.37 | 2483.23 | 1.32 |
|  | ILMN_1795930 | PTGER4 | NM_000958.2 | 2345.48 | 3071.37 | 1.31 |
|  | ILMN_1797005 | PGLS | NM_012088.2 | 859.89 | 1134.89 | 1.32 |
|  | ILMN_1801941 | C1orf50 | NM_024097.1 | 618.23 | 815.57 | 1.32 |
|  | ILMN_2049303 | DCI | NM_001919.2 | 403.01 | 533.25 | 1.32 |
|  | ILMN_2054297 | PTGS2 | NM_000963.1 | 1303.94 | 1706.31 | 1.31 |
|  | ILMN_2063584 | CLIC4 | NM_013943.1 | 3242 | 4278.83 | 1.32 |
|  | ILMN_2086095 | ID2 | NM_002166.4 | 235.89 | 311.91 | 1.32 |
|  | ILMN_2094938 | OMA1 | NM_145243.3 | 195.9 | 256.95 | 1.31 |
|  | ILMN_2106167 | RAP1GDS1 | NM_021159.3 | 636.79 | 836.18 | 1.31 |
|  | ILMN_2113938 | TOR1AIP2 | NM_145034.1 | 261.8 | 346.33 | 1.32 |
|  | ILMN_2129234 | TMEM47 | NM_031442.2 | 818.59 | 1070.94 | 1.31 |
|  | ILMN_2137789 | KLF4 | NM_004235.3 | 151.03 | 199.84 | 1.32 |
|  | ILMN_2147503 | ALG13 | NM_018466.2 | 449.44 | 589.36 | 1.31 |
|  | ILMN_2175131 | TMEM14C | NM_016462.2 | 3097.74 | 4053.63 | 1.31 |
|  | ILMN_2184184 | ANXA1 | NM_000700.1 | 10555.2 | 13828.28 | 1.31 |
|  | ILMN_2222317 | DNAJB4 | NM_007034.3 | 1304.55 | 1709.08 | 1.31 |
|  | ILMN_2227573 | GSTO1 | NM_004832.1 | 9495.31 | 12561.03 | 1.32 |
|  | ILMN_2289924 | TRAK1 | NM_001042646.1 | 396.45 | 523.48 | 1.32 |
|  | ILMN_2296950 | APOBEC3F | NM_001006666.1 | 231.36 | 303.38 | 1.31 |
|  | ILMN_2313901 | PAM | NM_138821.1 | 2880.96 | 3814.65 | 1.32 |
|  | ILMN_2319910 | DGKA | NM_201554.1 | 215.92 | 285.43 | 1.32 |
|  | ILMN_2336133 | SULT1A4 | NM_001017391.1 | 213.49 | 282.09 | 1.32 |
|  | ILMN_2356654 | LGALS8 | NM_201545.1 | 658.94 | 865.47 | 1.31 |
|  | ILMN_2362581 | FNDC3A | NM_001079673.1 | 770.69 | 1017.87 | 1.32 |
|  | ILMN_2367113 | CASP6 | NM_032992.2 | 702.33 | 930.16 | 1.32 |
|  | ILMN_2391150 | FILIP1L | NM_001042459.1 | 154.59 | 202.25 | 1.31 |
|  | ILMN_2413816 | GRB14 | NM_004490.2 | 1529.31 | 2022.14 | 1.32 |
|  | ILMN_3191922 | KRT8P9 | XR_017231.2 | 353.44 | 464.76 | 1.31 |
|  | ILMN_3199955 | LOC645430 | XR_018764.2 | 356.07 | 469.07 | 1.32 |
|  | ILMN_3247848 | NCRNA00085 | NR_024330.1 | 246.31 | 322.61 | 1.31 |
|  | ILMN_3272378 | EZR | NM_003379.4 | 5186.94 | 6845.78 | 1.32 |
|  | ILMN_3301740 | LOC729887 | XR_040891.1 | 329.39 | 435.64 | 1.32 |
|  | ILMN_1656088 | C1orf110 | NM_178550.3 | 141.99 | 188.27 | 1.33 |
|  | ILMN_1661599 | DDIT4 | NM_019058.2 | 360.79 | 482.93 | 1.34 |
|  | ILMN_1663685 | DGCR6 | NM_005675.2 | 1233.04 | 1639.83 | 1.33 |
|  | ILMN_1664802 | WSB1 | NM_134265.2 | 1316.36 | 1747.41 | 1.33 |
|  | ILMN_1672940 | ZNF562 | NM_017656.2 | 364.14 | 484.72 | 1.33 |
|  | ILMN_1673409 | MGC16121 | XM_001128419.1 | 195.14 | 258.74 | 1.33 |
|  | ILMN_1681703 | FOXO3 | NM_201559.2 | 444.8 | 589.36 | 1.33 |
|  | ILMN_1684336 | VEPH1 | NM_024621.1 | 230.29 | 305.78 | 1.33 |
|  | ILMN_1687887 | PSMC4 | NM_006503.2 | 417.12 | 557.05 | 1.34 |

|  | Probe ID | Symbol | Genebank accession | siControl | siSIRT6 | Fold ratio (siSIRT6/siControl) |
| --- | --- | --- | --- | --- | --- | --- |
|  | ILMN_1693341 | SNRPN | NM_022806.2 | 911.44 | 1212.41 | 1.33 |
|  | ILMN_1693401 | KLHL28 | NM_017658.3 | 235.95 | 315.9 | 1.34 |
|  | ILMN_1694432 | CRIP2 | NM_001312.2 | 3123.61 | 4150.3 | 1.33 |
|  | ILMN_1696510 | GPR89C | NM_001097616.1 | 295.57 | 395.63 | 1.34 |
|  | ILMN_1699651 | IL6 | NM_000600.1 | 325.46 | 432.93 | 1.33 |
|  | ILMN_1710752 | NAPRT1 | NM_145201.3 | 213.14 | 284.18 | 1.33 |
|  | ILMN_1716057 | CAPN2 | NM_001748.3 | 693.78 | 925.66 | 1.33 |
|  | ILMN_1717234 | CAST | NM_001042445.1 | 764.3 | 1019.28 | 1.33 |
|  | ILMN_1717706 | PLK2 | NM_006622.2 | 304.51 | 404.88 | 1.33 |
|  | ILMN_1718769 | ITSN1 | NM_001001132.1 | 732.15 | 976.63 | 1.33 |
|  | ILMN_1720838 | DECR1 | NM_001359.1 | 2484.95 | 3322.38 | 1.34 |
|  | ILMN_1726222 | FLOT2 | NM_004475.2 | 2407.52 | 3207.72 | 1.33 |
|  | ILMN_1727315 | DENND1A | NM_024820.2 | 318.1 | 426.38 | 1.34 |
|  | ILMN_1733110 | RASSF7 | NM_003475.2 | 481.15 | 644.78 | 1.34 |
|  | ILMN_1737394 | LMNA | NM_005572.3 | 2520.8 | 3367.98 | 1.34 |
|  | ILMN_1737650 | DIO2 | NM_001007023.2 | 152.92 | 205.17 | 1.34 |
|  | ILMN_1738335 | TNFSF18 | NM_005092.2 | 269.35 | 357.22 | 1.33 |
|  | ILMN_1738955 | C10orf104 | NM_173473.2 | 213.44 | 283.79 | 1.33 |
|  | ILMN_1739770 | CRSP9 | NM_004270.3 | 370.42 | 496.39 | 1.34 |
|  | ILMN_1739885 | SLC41A3 | NM_001008485.1 | 529.81 | 701.84 | 1.32 |
|  | ILMN_1752988 | C11orf17 | NM_182901.2 | 880.8 | 1179.81 | 1.34 |
|  | ILMN_1754279 | FBXW7 | NM_033632.2 | 435.64 | 577.5 | 1.33 |
|  | ILMN_1757604 | TPM2 | NM_213674.1 | 3465.87 | 4640.29 | 1.34 |
|  | ILMN_1760062 | IFI44 | NM_006417.3 | 514.37 | 688.67 | 1.34 |
|  | ILMN_1764380 | GLTP | NM_016433.3 | 5592.72 | 7494.76 | 1.34 |
|  | ILMN_1773018 | CUEDC2 | NM_024040.2 | 1112.05 | 1483.03 | 1.33 |
|  | ILMN_1773079 | COL3A1 | NM_000090.3 | 143.54 | 192.54 | 1.34 |
|  | ILMN_1774066 | TMEM141 | NM_032928.2 | 640.92 | 849.42 | 1.33 |

|  | Probe ID | Symbol | Genebank accession | siControl | siSIRT6 | Fold ratio (siSIRT6/siControl) |
| --- | --- | --- | --- | --- | --- | --- |
|  | ILMN_1774604 | PNKD | NM_022572.2 | 266.56 | 353.93 | 1.33 |
|  | ILMN_1778677 | MRS2 | NM_020662.2 | 250.38 | 332.53 | 1.33 |
|  | ILMN_1787324 | C16orf48 | NM_032140.1 | 766.96 | 1017.16 | 1.33 |
|  | ILMN_1793616 | RNF38 | NM_022781.4 | 685.02 | 913.55 | 1.33 |
|  | ILMN_1794230 | SCAND1 | NM_016558.2 | 1044.31 | 1386.28 | 1.33 |
|  | ILMN_1799600 | STARD8 | NM_014725.2 | 239.74 | 318.1 | 1.33 |
|  | ILMN_1802205 | RHOB | NM_004040.2 | 2630.28 | 3508.57 | 1.33 |
|  | ILMN_1812769 | UBXN1 | NM_015853.3 | 2563.09 | 3419.73 | 1.33 |
|  | ILMN_1813019 | DNAJB4 | NM_007034.3 | 1042.14 | 1390.77 | 1.33 |
|  | ILMN_1815086 | NINJ1 | NM_004148.3 | 913.33 | 1220.28 | 1.34 |
|  | ILMN_1815445 | IDS | NM_006123.2 | 383.48 | 508.35 | 1.33 |
|  | ILMN_1823231 |  | CR603183 | 236.88 | 314.08 | 1.33 |
|  | ILMN_1839019 |  | BX641108 | 589.9 | 786.7 | 1.33 |
|  | ILMN_2089329 | SPRY2 | NM_005842.2 | 1659.27 | 2214.86 | 1.33 |
|  | ILMN_2094166 | CHMP5 | NM_016410.2 | 567.31 | 759.37 | 1.34 |
|  | ILMN_2133316 | GIMAP7 | NM_153236.3 | 473.65 | 634 | 1.34 |
|  | ILMN_2151281 | GABARAPL1 | NM_031412.2 | 473.54 | 629.33 | 1.33 |
|  | ILMN_2169761 | CPNE8 | NM_153634.2 | 602.16 | 804.71 | 1.34 |
|  | ILMN_2230035 | BBS2 | NM_031885.2 | 380.65 | 505.07 | 1.33 |
|  | ILMN_2262901 | RUFY3 | NM_001037442.1 | 422.95 | 563.65 | 1.33 |
|  | ILMN_2278433 | LOC285074 | NM_001012626.1 | 339.28 | 452.05 | 1.33 |
|  | ILMN_2286870 | CSNK1D | NM_139062.1 | 730.8 | 974.6 | 1.33 |
|  | ILMN_2311166 | ITGB5 | NM_002213.3 | 2285.56 | 3032.59 | 1.33 |
|  | ILMN_2329429 | GIMAP6 | NM_001007224.1 | 964.3 | 1282.73 | 1.33 |
|  | ILMN_2372011 | SCAND1 | NM_033630.1 | 776.05 | 1041.42 | 1.34 |
|  | ILMN_2380698 | DSTN | NM_006870.3 | 8549.75 | 11346.82 | 1.33 |
|  | ILMN_2396956 | AKAP13 | NM_007200.3 | 235.62 | 312.92 | 1.33 |
|  | ILMN_2409062 | ISCU | NM_213595.1 | 1727.74 | 2297.74 | 1.33 |
|  | ILMN_3209631 | LOC728060 | XR_015272.2 | 400.59 | 531.9 | 1.33 |
|  | ILMN_3233930 | LOC390557 | XM_001726973.1 | 3391.4 | 4546.9 | 1.34 |
|  | ILMN_3237991 | LOC645166 | XM_001129441.2 | 1128.87 | 1506.17 | 1.33 |
|  | ILMN_3240594 | RNU4ATAC | NR_023343.1 | 136.62 | 181.73 | 1.33 |
|  | ILMN_3251691 | POLR3G | NM_006467.2 | 742.54 | 990.26 | 1.33 |
|  | ILMN_3310065 | SFTA1P | NR_027082.1 | 155.17 | 205.93 | 1.33 |
|  | ILMN_1651554 | RGS5 | NM_003617.2 | 1625.5 | 2184.37 | 1.34 |
|  | ILMN_1653133 | SH3D19 | NM_001009555.2 | 700.54 | 948.61 | 1.35 |
|  | ILMN_1654552 | MRPS31 | NM_005830.2 | 1057.18 | 1430.2 | 1.35 |
|  | ILMN_1656378 | NMT2 | NM_004808.1 | 1345.88 | 1816.57 | 1.35 |
|  | ILMN_1659240 | MTMR14 | NM_001077525.1 | 755.35 | 1018.57 | 1.35 |
|  | ILMN_1663489 | UBR2 | NM_015255.1 | 383.74 | 519.39 | 1.35 |
|  | ILMN_1666924 | PINK1 | NM_032409.2 | 210.6 | 285.04 | 1.35 |
|  | ILMN_1670875 | PPM1D | NM_003620.2 | 540.94 | 730.46 | 1.35 |
|  | ILMN_1671969 | UGP2 | NM_006759.3 | 296.66 | 402.73 | 1.36 |
|  | ILMN_1673305 | RHOC | NM_001042678.1 | 5597.89 | 7538.18 | 1.35 |

|  | Probe ID | Symbol | Genebank accession | siControl | siSIRT6 | Fold ratio (siSIRT6/siControl) |
| --- | --- | --- | --- | --- | --- | --- |
|  | ILMN_1680347 | ZNF317 | NM_020933.2 | 324.78 | 437.26 | 1.35 |
|  | ILMN_1690546 | PPP3CC | NM_005605.3 | 433.13 | 585.56 | 1.35 |
|  | ILMN_1693685 | LOC205251 | XR_017711.1 | 783.8 | 1056.93 | 1.35 |
|  | ILMN_1701655 | SLC24A6 | NM_024959.2 | 1074.91 | 1445.81 | 1.35 |
|  | ILMN_1705250 | ADAMTSL1 | NM_052866.3 | 189.67 | 256.24 | 1.35 |
|  | ILMN_1715748 | FLNC | NM_001458.3 | 3177.48 | 4281.79 | 1.35 |
|  | ILMN_1731048 | TLR1 | NM_003263.3 | 320.24 | 435.04 | 1.36 |
|  | ILMN_1731714 | CREB5 | NM_182898.2 | 225.71 | 306.84 | 1.36 |
|  | ILMN_1733799 | FAM195B | NM_207368.3 | 514.25 | 699.09 | 1.36 |
|  | ILMN_1741176 | CHMP2B | NM_014043.2 | 473.32 | 640.33 | 1.35 |
|  | ILMN_1742827 | EXOC4 | NM_021807.3 | 430.44 | 578.16 | 1.34 |
|  | ILMN_1743187 | C6orf120 | NM_001029863.1 | 267.24 | 362.21 | 1.36 |
|  | ILMN_1765833 | SLC9A3R2 | NM_004785.3 | 185.76 | 252.13 | 1.36 |
|  | ILMN_1766657 | STOM | NM_004099.4 | 3696.64 | 4976.79 | 1.35 |
|  | ILMN_1777061 | ZSWIM6 | XM_035299.8 | 1221.98 | 1642.87 | 1.34 |
|  | ILMN_1779034 | NADSYN1 | NM_018161.4 | 497.88 | 673.41 | 1.35 |
|  | ILMN_1779071 | FEZ1 | NM_005103.3 | 1174.37 | 1588.01 | 1.35 |
|  | ILMN_1781472 | CDC42BPA | NM_003607.3 | 293.59 | 396.73 | 1.35 |
|  | ILMN_1782939 | ALB | NM_000477.3 | 149.57 | 203.14 | 1.36 |
|  | ILMN_1789196 | TPM2 | NM_213674.1 | 5194.13 | 6983.17 | 1.34 |
|  | ILMN_1790637 | C11orf80 | NM_024650.2 | 326.14 | 437.96 | 1.34 |
|  | ILMN_1797822 | SEL1L3 | NM_015187.3 | 1049.63 | 1412.14 | 1.35 |
|  | ILMN_1798372 | ANXA2P3 | NR_001446.2 | 1343.08 | 1817.41 | 1.35 |
|  | ILMN_1802690 | GULP1 | NM_016315.2 | 167.81 | 227.18 | 1.35 |
|  | ILMN_1806266 | RAP1GDS1 | NM_021159.3 | 625.27 | 839.86 | 1.34 |
|  | ILMN_1808196 | GSTO1 | NM_004832.1 | 5184.54 | 6981.56 | 1.35 |
|  | ILMN_1808769 | C1orf97 | NM_032705.2 | 239.52 | 321.94 | 1.34 |
|  | ILMN_1815283 | SULT1A3 | NM_003166.3 | 218.43 | 294.27 | 1.35 |
|  | ILMN_1873863 |  | CR622110 | 245.46 | 331.53 | 1.35 |
|  | ILMN_2096083 | PLEKHA5 | NM_019012.2 | 261.2 | 352.87 | 1.35 |
|  | ILMN_2122420 | HMGCL | NM_000191.2 | 224.88 | 303.03 | 1.35 |
|  | ILMN_2153466 | FAM50B | NM_012135.1 | 242.58 | 326.29 | 1.35 |
|  | ILMN_2159272 | PDCD1LG2 | NM_025239.2 | 139.33 | 189.45 | 1.36 |
|  | ILMN_2181445 | BCL2L13 | NM_015367.2 | 1391.09 | 1892.4 | 1.36 |
|  | ILMN_2184612 | C3orf52 | NM_024616.1 | 181.48 | 245.4 | 1.35 |
|  | ILMN_2229170 | CRSP9 | NM_004270.3 | 383.04 | 519.39 | 1.36 |
|  | ILMN_2292387 | TANK | NM_004180.2 | 805.64 | 1085.39 | 1.35 |
|  | ILMN_2300970 | ETFB | NM_001014763.1 | 952.12 | 1292.25 | 1.36 |
|  | ILMN_2318568 | HCFC1R1 | NM_001002018.1 | 343.94 | 462.08 | 1.34 |
|  | ILMN_2322842 | PPHLN1 | NM_201440.1 | 1054.01 | 1431.85 | 1.36 |
|  | ILMN_2338722 | NRXN3 | NM_004796.3 | 234.54 | 317.51 | 1.35 |
|  | ILMN_2384056 | GPER | NM_001039966.1 | 141.8 | 191.74 | 1.35 |
|  | ILMN_2384241 | TGFBR2 | NM_001024847.1 | 5528.48 | 7444.71 | 1.35 |
|  | ILMN_2384591 | HN1 | NM_016185.2 | 1985.56 | 2668.85 | 1.34 |
|  | ILMN_2388177 | SPATA7 | NM_018418.2 | 273.99 | 369.56 | 1.35 |
|  | ILMN_2406043 | VPS24 | NM_001005753.1 | 815.76 | 1095.47 | 1.34 |

|  | Probe ID | Symbol | Genebank accession | siControl | siSIRT6 | Fold ratio (siSIRT6/siControl) |
| --- | --- | --- | --- | --- | --- | --- |
|  | ILMN_3178302 | FNDC3B | NM_001135095.1 | 1703.16 | 2309.45 | 1.36 |
|  | ILMN_3240520 | ELTD1 | NM_022159.3 | 2778.33 | 3744.78 | 1.35 |
|  | ILMN_3243244 | SNORD80 | NR_003940.1 | 665.06 | 899.93 | 1.35 |
|  | ILMN_3244640 | SNORD96A | NR_002592.1 | 290.76 | 393.08 | 1.35 |
|  | ILMN_1662232 | DCTN2 | NM_006400.3 | 3423.68 | 4674.73 | 1.37 |
|  | ILMN_1664303 | HTATIP2 | NM_006410.3 | 1734.94 | 2392.55 | 1.38 |
|  | ILMN_1669032 | PPIC | NM_000943.4 | 1227.92 | 1687.49 | 1.37 |
|  | ILMN_1671387 | C3orf23 | NM_001029840.2 | 252.65 | 345.61 | 1.37 |
|  | ILMN_1671928 | PROS1 | NM_000313.1 | 186.11 | 253.64 | 1.36 |
|  | ILMN_1676336 | AADACL1 | NM_020792.3 | 1104.11 | 1519.8 | 1.38 |
|  | ILMN_1676361 | ARHGAP22 | NM_021226.2 | 1057.42 | 1439.15 | 1.36 |
|  | ILMN_1682864 | SPSB3 | NM_080861.3 | 574.3 | 791.62 | 1.38 |
|  | ILMN_1689059 | ZNF329 | NM_024620.3 | 259.87 | 357.8 | 1.38 |
|  | ILMN_1691410 | BAMBI | NM_012342.2 | 457.2 | 622.67 | 1.36 |
|  | ILMN_1697642 | BCAP29 | NM_001008405.1 | 1353.99 | 1846.61 | 1.36 |
|  | ILMN_1697864 | CXorf38 | NM_144970.1 | 450.69 | 615.24 | 1.37 |
|  | ILMN_1702835 | SH3BGRL | NM_003022.1 | 1351.18 | 1849.18 | 1.37 |
|  | ILMN_1705814 | KRT80 | NM_182507.2 | 876.33 | 1198.77 | 1.37 |
|  | ILMN_1709043 | C9orf46 | NM_018465.2 | 1126.53 | 1541.73 | 1.37 |
|  | ILMN_1715788 | MMRN2 | NM_024756.1 | 477.93 | 655.14 | 1.37 |
|  | ILMN_1718718 | MKKS | NM_170784.1 | 537.83 | 737.24 | 1.37 |
|  | ILMN_1724040 | ANKRD57 | NM_023016.3 | 561.19 | 765.36 | 1.36 |
|  | ILMN_1725241 | GSTK1 | NM_015917.1 | 1438.82 | 1963.67 | 1.36 |
|  | ILMN_1735432 | ISCU | NM_014301.2 | 371.36 | 512 | 1.38 |
|  | ILMN_1736704 | DIXDC1 | NM_001037954.2 | 271.91 | 371.96 | 1.37 |
|  | ILMN_1745148 | ZNFX1 | NM_021035.2 | 310.48 | 422.85 | 1.36 |
|  | ILMN_1746664 | WSB2 | NM_018639.3 | 1824.14 | 2505.13 | 1.37 |
|  | ILMN_1750101 | S100A11 | NM_005620.1 | 2876.97 | 3960.13 | 1.38 |
|  | ILMN_1750961 | TM6SF1 | NM_023003.2 | 664.44 | 911.44 | 1.37 |
|  | ILMN_1751075 | SETD4 | NM_017438.2 | 288.28 | 396.36 | 1.37 |
|  | ILMN_1757408 | ZNF256 | NM_005773.2 | 210.94 | 290.15 | 1.38 |
|  | ILMN_1772459 | RPS23 | NM_001025.4 | 2235.94 | 3048.04 | 1.36 |
|  | ILMN_1774982 | CDC42EP5 | NM_145057.2 | 1152.06 | 1567.95 | 1.36 |
|  | ILMN_1775192 | BCLAF1 | NM_001077440.1 | 2204.14 | 3009.55 | 1.37 |
|  | ILMN_1784641 | NDUFA3 | NM_004542.2 | 2445.08 | 3357.1 | 1.37 |
|  | ILMN_1786021 | PRKAB2 | NM_005399.3 | 194.37 | 264.6 | 1.36 |
|  | ILMN_1787843 | HSDL2 | NM_032303.3 | 321.8 | 439.79 | 1.37 |
|  | ILMN_1788356 | C11orf17 | NM_182901.2 | 491.26 | 672.16 | 1.37 |
|  | ILMN_1795937 | VIL2 | NM_003379.3 | 2849.84 | 3916.46 | 1.37 |
|  | ILMN_1796335 | LPCAT2 | NM_017839.3 | 239.85 | 329.47 | 1.37 |
|  | ILMN_1803367 | EVI1 | NM_005241.1 | 1157.4 | 1581.05 | 1.37 |
|  | ILMN_1804396 | C14orf4 | NM_024496.2 | 619.66 | 845.11 | 1.36 |
|  | ILMN_1814737 | LNPEP | NM_175920.3 | 276.15 | 377.67 | 1.37 |
|  | ILMN_1814985 | PDLIM7 | NM_005451.3 | 267.86 | 369.22 | 1.38 |
|  | ILMN_2059535 | PPM1F | NM_014634.2 | 2583.9 | 3552.62 | 1.37 |
|  | ILMN_2062620 | NMT2 | NM_004808.2 | 400.04 | 549.51 | 1.37 |

|  | Probe ID | Symbol | Genebank accession | siControl | siSIRT6 | Fold ratio (siSIRT6/siControl) |
| --- | --- | --- | --- | --- | --- | --- |
|  | ILMN_2077905 | PTGFRN | NM_020440.2 | 332.99 | 457.09 | 1.37 |
|  | ILMN_2136455 | C3orf64 | NM_173654.1 | 970.33 | 1333.5 | 1.37 |
|  | ILMN_2185563 | ANKRA2 | NM_023039.2 | 397.37 | 545.34 | 1.37 |
|  | ILMN_2190051 | CCDC91 | NM_018318.3 | 293.52 | 401.34 | 1.37 |
|  | ILMN_2211780 | SLC25A4 | NM_001151.2 | 1375.43 | 1871.53 | 1.36 |
|  | ILMN_2314169 | PTHLH | NM_198965.1 | 188.88 | 258.68 | 1.37 |
|  | ILMN_2354953 | NQO1 | NM_000903.2 | 923.52 | 1266.82 | 1.37 |
|  | ILMN_2358980 | ILK | NM_001014794.1 | 1541.73 | 2117.29 | 1.37 |
|  | ILMN_2373982 | PICK1 | NM_012407.3 | 185.81 | 252.83 | 1.36 |
|  | ILMN_2388397 | LYPD1 | NM_001077427.2 | 385.88 | 527.37 | 1.37 |
|  | ILMN_2393046 | NHLRC3 | NM_001017370.1 | 191.03 | 261.14 | 1.37 |
|  | ILMN_2396672 | ABLIM1 | NM_001003407.1 | 200.48 | 275.33 | 1.37 |
|  | ILMN_3241665 | C1orf133 | NR_024337.1 | 282.67 | 384.9 | 1.36 |
|  | ILMN_3242900 | HIST2H2AA4 | NM_001040874.1 | 320.98 | 441.72 | 1.38 |
|  | ILMN_3252936 | KRT18P17 | XR_037953.1 | 550.4 | 758.5 | 1.38 |
|  | ILMN_1655952 | FAM39E | NM_182905.2 | 919.69 | 1276.81 | 1.39 |
|  | ILMN_1659027 | SLC2A1 | NM_006516.1 | 516.75 | 714.27 | 1.38 |
|  | ILMN_1667460 | SULF2 | NM_018837.2 | 595.1 | 829.25 | 1.39 |
|  | ILMN_1671568 | ECHDC2 | NM_018281.2 | 425.5 | 588.68 | 1.38 |
|  | ILMN_1673509 | RPL28 | NM_000991.3 | 229.5 | 317.66 | 1.38 |
|  | ILMN_1676238 | LOC440105 | XM_495926.2 | 465.72 | 645.53 | 1.39 |
|  | ILMN_1679133 | SERPINB1 | NM_030666.2 | 682.33 | 952.12 | 1.40 |
|  | ILMN_1687757 | AKR1C4 | NM_001818.2 | 179.06 | 248.43 | 1.39 |
|  | ILMN_1701514 | TRAF3IP2 | NM_147686.1 | 292.85 | 404.69 | 1.38 |
|  | ILMN_1706426 | DSTN | NM_001011546.1 | 6053.97 | 8420.36 | 1.39 |
|  | ILMN_1711069 | YPEL5 | NM_016061.1 | 575.5 | 795.1 | 1.38 |
|  | ILMN_1713990 | TRIP6 | NM_003302.2 | 1918.37 | 2676.87 | 1.40 |
|  | ILMN_1716342 | CCDC132 | NM_017667.2 | 341.8 | 473.54 | 1.39 |
|  | ILMN_1726245 | TGFBR2 | NM_001024847.2 | 3571.55 | 4936.71 | 1.38 |
|  | ILMN_1726516 | SCRIB | NM_015356.3 | 1105.9 | 1542.44 | 1.39 |
|  | ILMN_1728009 | TMEM171 | NM_173490.5 | 503.09 | 702.81 | 1.40 |
|  | ILMN_1741264 | MRPS33 | NM_016071.2 | 1109.74 | 1534.98 | 1.38 |
|  | ILMN_1745116 | ABHD12 | NM_015600.3 | 382.86 | 534.11 | 1.40 |
|  | ILMN_1755711 | C17orf68 | XM_938283.2 | 311.77 | 435.34 | 1.40 |
|  | ILMN_1756238 | TMEM217 | NM_145316.2 | 236.93 | 329.85 | 1.39 |
|  | ILMN_1763011 | C7orf10 | NM_024728.1 | 153.77 | 213.14 | 1.39 |
|  | ILMN_1771333 | CD47 | NM_198793.2 | 324.71 | 453.3 | 1.40 |
|  | ILMN_1775042 | WDR69 | NM_178821.1 | 398.56 | 555.51 | 1.39 |
|  | ILMN_1776094 | PPCS | NM_024664.2 | 1074.41 | 1498.18 | 1.39 |
|  | ILMN_1777449 | IFT74 | NM_001099222.1 | 390.63 | 539.69 | 1.38 |
|  | ILMN_1779674 | PTPRG | NM_002841.2 | 485.84 | 678.56 | 1.40 |
|  | ILMN_1784320 | ELMO1 | NM_014800.9 | 251.54 | 349.63 | 1.39 |
|  | ILMN_1784948 | SPOCD1 | NM_144569.4 | 1410.51 | 1950.55 | 1.38 |
|  | ILMN_1786429 | P2RY5 | NM_005767.4 | 202.91 | 282.94 | 1.39 |
|  | ILMN_1793025 | OSAP | NM_032623.2 | 1208.22 | 1675.06 | 1.39 |
|  | ILMN_1798448 | IDS | NM_006123.2 | 383.39 | 530.55 | 1.38 |

|  | Probe ID | Symbol | Genebank accession | siControl | siSIRT6 | Fold ratio (siSIRT6/siControl) |
| --- | --- | --- | --- | --- | --- | --- |
|  | ILMN_1798496 | HOXB8 | NM_024016.3 | 295.98 | 412.81 | 1.39 |
|  | ILMN_1803277 | MVP | NM_005115.3 | 1305.45 | 1810.28 | 1.39 |
|  | ILMN_1803564 | YIPF1 | NM_018982.3 | 348.25 | 481.26 | 1.38 |
|  | ILMN_1811102 | LRSAM1 | NM_138361.3 | 476.06 | 658.03 | 1.38 |
|  | ILMN_2043615 | C17orf90 | NM_001039842.1 | 624.55 | 867.87 | 1.39 |
|  | ILMN_2058795 | PGCP | NM_016134.2 | 199.28 | 277.31 | 1.39 |
|  | ILMN_2082273 | RGS5 | NM_003617.2 | 3822.59 | 5287.36 | 1.38 |
|  | ILMN_2095133 | SPTAN1 | NM_003127.1 | 555.12 | 775.87 | 1.40 |
|  | ILMN_2153332 | ATXN1 | NM_000332.2 | 269.79 | 376.02 | 1.39 |
|  | ILMN_2201678 | FSTL1 | NM_007085.3 | 2482.65 | 3435.57 | 1.38 |
|  | ILMN_2324574 | RALGAPA1 | NM_194301.2 | 293.52 | 407.97 | 1.39 |
|  | ILMN_2331010 | TNFRSF10B | NM_147187.1 | 306.91 | 426.78 | 1.39 |
|  | ILMN_2350574 | MYADM | NM_138373.3 | 1653.15 | 2282.4 | 1.38 |
|  | ILMN_3187508 | KRT18P13 | XM_001726959.1 | 221.22 | 307.97 | 1.39 |
|  | ILMN_3236112 | TP53TG1 | NR_015381.1 | 175.46 | 243.42 | 1.39 |
|  | ILMN_3251536 | FAM127C | NM_001078173.1 | 380.13 | 528.71 | 1.39 |
|  | ILMN_1652631 | GLIPR2 | NM_022343.2 | 1044.79 | 1465.32 | 1.40 |
|  | ILMN_1653039 | LOC642934 | XM_942991.2 | 1489.56 | 2094.42 | 1.41 |
|  | ILMN_1654685 | MCTP1 | NM_024717.3 | 266.01 | 373.08 | 1.40 |
|  | ILMN_1658483 | IL1A | NM_000575.3 | 184.23 | 260.96 | 1.42 |
|  | ILMN_1665065 | SERINC3 | NM_198941.1 | 146.05 | 204.27 | 1.40 |
|  | ILMN_1667948 | SPATA18 | NM_145263.2 | 183.21 | 258.2 | 1.41 |
|  | ILMN_1671123 | LOC647543 | XM_943178.1 | 377.41 | 528.95 | 1.40 |
|  | ILMN_1672389 | CRYZ | NM_001889.2 | 1615.02 | 2263.49 | 1.40 |
|  | ILMN_1685397 | ITGA3 | NM_002204.1 | 581.78 | 820.86 | 1.41 |
|  | ILMN_1687084 | C3orf64 | NM_173654.1 | 419.83 | 593.87 | 1.41 |
|  | ILMN_1687785 | PPA2 | NM_176866.2 | 2093.45 | 2927.93 | 1.40 |
|  | ILMN_1689318 | NUAK1 | NM_014840.2 | 432.53 | 612.83 | 1.42 |
|  | ILMN_1691111 | SPATA2L | NM_152339.2 | 431.14 | 611.27 | 1.42 |
|  | ILMN_1694458 | FLJ41200 | XM_379623.3 | 318.32 | 447.17 | 1.40 |
|  | ILMN_1697220 | NT5E | NM_002526.1 | 1584.34 | 2243.7 | 1.42 |
|  | ILMN_1701077 | LOC642897 | XR_017554.1 | 460.38 | 647.17 | 1.41 |
|  | ILMN_1706571 | SLC35D2 | NM_007001.1 | 359.29 | 508.58 | 1.42 |
|  | ILMN_1708110 | TMEM144 | NM_018342.3 | 152.54 | 213.73 | 1.40 |
|  | ILMN_1715693 | LOC440160 | XM_498571.2 | 304.3 | 426.58 | 1.40 |
|  | ILMN_1723768 | NLRX1 | NM_170722.1 | 201.6 | 283.53 | 1.41 |
|  | ILMN_1730487 | CALD1 | NM_033140.2 | 3389.84 | 4770.75 | 1.41 |
|  | ILMN_1744604 | CYBA | NM_000101.2 | 1223.11 | 1722.95 | 1.41 |
|  | ILMN_1747020 | SGK3 | NM_001033578.1 | 223.53 | 312.85 | 1.40 |
|  | ILMN_1753426 | KIAA0556 | NM_015202.1 | 188.14 | 263.68 | 1.40 |
|  | ILMN_1757186 | GIMAP1 | NM_130759.2 | 531.77 | 746.67 | 1.40 |
|  | ILMN_1761044 | GNB1L | NM_053004.2 | 335 | 471.35 | 1.41 |
|  | ILMN_1768505 | IL13RA1 | NM_001560.2 | 1583.97 | 2242.67 | 1.42 |
|  | ILMN_1769633 | CTSO | NM_001334.2 | 180.6 | 255.53 | 1.41 |
|  | ILMN_1775330 | C15orf52 | NM_207380.1 | 565.22 | 793.27 | 1.40 |
|  | ILMN_1776213 | RGMB | NM_001012761.1 | 794 | 1119.78 | 1.41 |

|  | Probe ID | Symbol | Genebank accession | siControl | siSIRT6 | Fold ratio (siSIRT6/siControl) |
| --- | --- | --- | --- | --- | --- | --- |
|  | ILMN_1777378 | COMMD6 | NM_203497.2 | 3902.01 | 5524.65 | 1.42 |
|  | ILMN_1784847 | CREBZF | NM_001039618.1 | 186.93 | 264.84 | 1.42 |
|  | ILMN_1790797 | VPS28 | NM_016208.2 | 928.23 | 1313.92 | 1.42 |
|  | ILMN_1793118 | TAX1BP1 | NM_001079864.1 | 1934.84 | 2706.1 | 1.40 |
|  | ILMN_1794803 | NDP | NM_000266.1 | 304.37 | 427.27 | 1.40 |
|  | ILMN_1796074 | C18orf56 | NM_001012716.1 | 235.89 | 333.3 | 1.41 |
|  | ILMN_1813685 | RAB7L1 | NM_003929.1 | 341.4 | 480.15 | 1.41 |
|  | ILMN_1847308 |  | D87470 | 279.75 | 393.62 | 1.41 |
|  | ILMN_1882590 |  | AK094914 | 353.36 | 497.08 | 1.41 |
|  | ILMN_2115434 | RAB32 | NM_006834.2 | 2337.9 | 3306.3 | 1.41 |
|  | ILMN_2118229 | NAPEPLD | NM_198990.3 | 264.84 | 373.51 | 1.41 |
|  | ILMN_2142752 | MANSC1 | NM_018050.2 | 511.05 | 717.08 | 1.40 |
|  | ILMN_2150654 | ZSWIM4 | NM_023072.1 | 277.56 | 389.46 | 1.40 |
|  | ILMN_2159453 | STXBP2 | NM_006949.1 | 349.63 | 489.56 | 1.40 |
|  | ILMN_2184789 | HSCB | NM_172002.3 | 624.26 | 884.67 | 1.42 |
|  | ILMN_2354381 | PON2 | NM_000305.2 | 3243.49 | 4593.36 | 1.42 |
|  | ILMN_2371825 | AGL | NM_000645.2 | 383.21 | 539.57 | 1.41 |
|  | ILMN_2374770 | TAX1BP1 | NM_001079864.1 | 1414.42 | 1992.46 | 1.41 |
|  | ILMN_2388800 | PPAP2B | NM_003713.3 | 1072.68 | 1519.8 | 1.42 |
|  | ILMN_2393450 | C14orf173 | NM_022489.2 | 1452.85 | 2035.26 | 1.40 |
|  | ILMN_3251565 | RNASEH2C | NM_032193.3 | 181.56 | 256.47 | 1.41 |
|  | ILMN_1651282 | COL17A1 | NM_000494.3 | 270.91 | 388.65 | 1.43 |
|  | ILMN_1653161 | SNCG | NM_003087.1 | 196.86 | 281.37 | 1.43 |
|  | ILMN_1656185 | DEF8 | NM_017702.2 | 1599.42 | 2275.02 | 1.42 |
|  | ILMN_1659297 | FZD6 | NM_003506.2 | 1021.64 | 1469.73 | 1.44 |
|  | ILMN_1662865 | CPPED1 | NM_018340.2 | 250.96 | 357.47 | 1.42 |
|  | ILMN_1666819 | PHLDB1 | NM_015157.1 | 824.67 | 1179.53 | 1.43 |
|  | ILMN_1674629 | C9orf3 | NM_032823.3 | 141.3 | 200.9 | 1.42 |
|  | ILMN_1676563 | HTRA1 | NM_002775.3 | 1222.54 | 1737.34 | 1.42 |
|  | ILMN_1688629 | ZNF274 | NM_016324.2 | 451.74 | 645.97 | 1.43 |
|  | ILMN_1694075 | GADD45A | NM_001924.2 | 592.91 | 844.92 | 1.43 |
|  | ILMN_1695423 | CD9 | NM_001769.2 | 5158.25 | 7417.24 | 1.44 |
|  | ILMN_1696183 | HBQ1 | NM_005331.3 | 143.91 | 204.32 | 1.42 |
|  | ILMN_1705111 | FNDC3A | NM_001079673.1 | 756.57 | 1073.17 | 1.42 |
|  | ILMN_1706217 | TLR4 | NM_138554.2 | 218.07 | 312.63 | 1.43 |
|  | ILMN_1714158 | PON2 | NM_000305.2 | 2213.33 | 3142.43 | 1.42 |
|  | ILMN_1716265 | PGM2L1 | NM_173582.3 | 360.95 | 519.15 | 1.44 |
|  | ILMN_1721703 | PNN | NM_002687.3 | 1088.66 | 1564.7 | 1.44 |
|  | ILMN_1727815 | CFI | NM_000204.2 | 244.16 | 350.43 | 1.44 |
|  | ILMN_1738742 | PLAT | NM_000930.2 | 1291.65 | 1858.17 | 1.44 |
|  | ILMN_1765159 | ELMOD2 | NM_153702.1 | 231.95 | 329.93 | 1.42 |
|  | ILMN_1770612 | KRT15 | NM_002275.2 | 193.03 | 277.31 | 1.44 |
|  | ILMN_1771261 | SYNC1 | NM_030786.1 | 165.54 | 236 | 1.43 |
|  | ILMN_1779333 | MSRB3 | NM_001031679.1 | 142.95 | 204.03 | 1.43 |
|  | ILMN_1780861 | LOC653506 | XM_927769.1 | 638.41 | 913.97 | 1.43 |
|  |  |  |  |  |  |  |

|  | Probe ID | Symbol | Genebank accession | siControl | siSIRT6 | Fold ratio (siSIRT6/siControl) |
| --- | --- | --- | --- | --- | --- | --- |
|  | ILMN_1781560 | ST3GAL6 | NM_006100.2 | 306.55 | 437.05 | 1.43 |
|  | ILMN_1798360 | CXCR7 | NM_020311.2 | 790.89 | 1130.96 | 1.43 |
|  | ILMN_2041222 | FLJ40504 | NM_173624.1 | 2979.8 | 4254.18 | 1.43 |
|  | ILMN_2066348 | HERPUD2 | NM_022373.3 | 403.57 | 575.9 | 1.43 |
|  | ILMN_2194229 | TMEM128 | NM_032927.2 | 342.59 | 488.31 | 1.43 |
|  | ILMN_2284181 | UGP2 | NM_006759.3 | 237.21 | 338.89 | 1.43 |
|  | ILMN_2368530 | IL32 | NM_001012633.1 | 295.5 | 424.81 | 1.44 |
|  | ILMN_2413158 | PODXL | NM_001018111.1 | 2923.2 | 4167.6 | 1.43 |
|  | ILMN_3263225 | LOC100130506 | XM_001724500.1 | 715.43 | 1021.64 | 1.43 |
|  | ILMN_1651254 | LPP | NM_005578.2 | 1989.24 | 2885.62 | 1.45 |
|  | ILMN_1657446 | C1orf57 | NM_032324.1 | 726.09 | 1054.25 | 1.45 |
|  | ILMN_1661428 | ATP11C | NM_173694.3 | 197.91 | 286.55 | 1.45 |
|  | ILMN_1663032 | FNDC4 | NM_022823.1 | 473.65 | 685.97 | 1.45 |
|  | ILMN_1675062 | MYL9 | NM_006097.3 | 261.14 | 379.34 | 1.45 |
|  | ILMN_1677385 | C8orf40 | NM_138436.2 | 499.04 | 721.24 | 1.45 |
|  | ILMN_1682930 | SIPA1 | NM_006747.2 | 840.25 | 1221.69 | 1.45 |
|  | ILMN_1697227 | USP36 | NM_025090.2 | 220.91 | 318.25 | 1.44 |
|  | ILMN_1700306 | OCIAD2 | NM_001014446.1 | 4366.72 | 6357.89 | 1.46 |
|  | ILMN_1703379 | VPS41 | NM_014396.2 | 937.71 | 1359.95 | 1.45 |
|  | ILMN_1703852 | EFNB2 | NM_004093.2 | 498.11 | 718.08 | 1.44 |
|  | ILMN_1739521 | NLGN1 | NM_014932.2 | 577.76 | 839.47 | 1.45 |
|  | ILMN_1743755 | LOC441150 | XM_001134411.1 | 152.85 | 221.27 | 1.45 |
|  | ILMN_1756877 | C14orf179 | NM_052873.1 | 508.35 | 738.1 | 1.45 |
|  | ILMN_1774074 | RXRB | NM_021976.3 | 480.59 | 692.02 | 1.44 |
|  | ILMN_1778010 | IL32 | NM_001012636.1 | 184.27 | 268.11 | 1.45 |
|  | ILMN_1781285 | DUSP1 | NM_004417.2 | 2425.39 | 3510.19 | 1.45 |
|  | ILMN_1782487 | LOC400759 | NR_003133.1 | 195.95 | 283 | 1.44 |
|  | ILMN_1783276 | NEXN | NM_144573.3 | 576.43 | 841.22 | 1.46 |
|  | ILMN_1788192 | ARMCX1 | NM_016608.1 | 581.38 | 843.36 | 1.45 |
|  | ILMN_1797522 | DUSP3 | NM_004090.2 | 1535.33 | 2226.66 | 1.45 |
|  | ILMN_2079786 | NUAK1 | NM_014840.2 | 523.97 | 757.62 | 1.45 |
|  | ILMN_2081883 | IQCK | NM_153208.1 | 440.2 | 639 | 1.45 |
|  | ILMN_2140799 | FAM24B | NM_152644.2 | 598 | 868.27 | 1.45 |
|  | ILMN_2364852 | BTN2A1 | NM_007049.2 | 663.83 | 956.97 | 1.44 |
|  | ILMN_2367191 | PSMF1 | NM_178579.1 | 1589.84 | 2313.72 | 1.46 |
|  | ILMN_3191030 | LOC100129960 | XR_037304.1 | 722.74 | 1044.55 | 1.45 |
|  | ILMN_3253456 | FNDC3B | NM_022763.3 | 1433.18 | 2066.06 | 1.44 |
|  | ILMN_3265193 | LOC100129186 | XM_001722466.1 | 251.08 | 363.55 | 1.45 |
|  | ILMN_1652826 | LRRC17 | NM_005824.1 | 291.09 | 430.74 | 1.48 |
|  | ILMN_1659845 | KIAA0355 | NM_014686.3 | 854.14 | 1260.11 | 1.48 |
|  | ILMN_1660871 | NEK6 | NM_014397.3 | 539.2 | 791.07 | 1.47 |
|  | ILMN_1670272 | LRP10 | NM_014045.3 | 2398.64 | 3507.76 | 1.46 |
|  | ILMN_1676897 | HSPA12B | NM_052970.3 | 347.37 | 508.23 | 1.46 |
|  | ILMN_1680364 | CD109 | NM_133493.2 | 201.04 | 293.93 | 1.46 |
|  | ILMN_1686957 | SS18 | NM_001007559.1 | 135.8 | 198.73 | 1.46 |
|  | ILMN_1686981 | SULF2 | NM_018837.2 | 265.89 | 393.62 | 1.48 |

|  | Probe ID | Symbol | Genebank accession | siControl | siSIRT6 | Fold ratio (siSIRT6/siControl) |
| --- | --- | --- | --- | --- | --- | --- |
|  | ILMN_1692058 | NDN | NM_002487.2 | 945.33 | 1384.04 | 1.46 |
|  | ILMN_1705213 | TMBIM1 | NM_022152.4 | 1108.45 | 1636.81 | 1.48 |
|  | ILMN_1706505 | COL5A1 | NM_000093.3 | 828.29 | 1226.78 | 1.48 |
|  | ILMN_1720708 | CSNK1D | NM_001893.3 | 1606.83 | 2348.19 | 1.46 |
|  | ILMN_1721657 | RSU1 | NM_012425.3 | 850.99 | 1256.91 | 1.48 |
|  | ILMN_1737972 | TSPYL5 | NM_033512.2 | 267.12 | 390.81 | 1.46 |
|  | ILMN_1744046 | DIAPH2 | NM_006729.3 | 360.62 | 531.65 | 1.47 |
|  | ILMN_1745497 | C12orf26 | NM_032230.1 | 335.85 | 496.62 | 1.48 |
|  | ILMN_1751079 | TAP1 | NM_000593.5 | 539.45 | 791.26 | 1.47 |
|  | ILMN_1752478 | DHRS3 | NM_004753.4 | 230.03 | 336.63 | 1.46 |
|  | ILMN_1760493 | LIMS2 | NM_017980.3 | 153.56 | 226.18 | 1.47 |
|  | ILMN_1773643 | SLC38A9 | NM_173514.1 | 256.65 | 375.76 | 1.46 |
|  | ILMN_1788250 | LDOC1 | NM_012317.2 | 787.97 | 1167.33 | 1.48 |
|  | ILMN_1789830 | CFLAR | NM_003879.3 | 978.66 | 1430.2 | 1.46 |
|  | ILMN_1791306 | C9orf103 | NM_001001551.1 | 202.58 | 298.86 | 1.48 |
|  | ILMN_1799579 | CCDC51 | NM_024661.3 | 320.76 | 472.44 | 1.47 |
|  | ILMN_1805643 | RILPL1 | NM_178314.2 | 838.69 | 1226.78 | 1.46 |
|  | ILMN_2075051 | PGS1 | NM_024419.3 | 562.48 | 824.29 | 1.47 |
|  | ILMN_2098743 | THEM2 | NM_018473.2 | 689.94 | 1014.81 | 1.47 |
|  | ILMN_2188204 | ATG12 | NM_004707.2 | 1239.32 | 1819.93 | 1.47 |
|  | ILMN_2223903 | PPIC | NM_000943.4 | 983.88 | 1455.53 | 1.48 |
|  | ILMN_2367063 | TPK1 | NM_001042482.1 | 241.74 | 353.69 | 1.46 |
|  | ILMN_2376263 | SMARCA1 | NM_003069.2 | 486.4 | 713.61 | 1.47 |
|  | ILMN_2413527 | VCL | NM_003373.3 | 330.31 | 485.95 | 1.47 |
|  | ILMN_1668582 | CRBN | NM_016302.2 | 404.22 | 600.77 | 1.49 |
|  | ILMN_1671893 | CHMP2A | NM_014453.2 | 1102.07 | 1636.43 | 1.48 |
|  | ILMN_1673320 | C18orf34 | NM_198995.1 | 165.96 | 246.71 | 1.49 |
|  | ILMN_1681670 | SLC25A4 | NM_001151.2 | 428.36 | 641.36 | 1.50 |
|  | ILMN_1688639 | FBXL2 | NM_012157.2 | 380.04 | 569.15 | 1.50 |
|  | ILMN_1691572 | TST | NM_003312.4 | 1579.22 | 2341.69 | 1.48 |
|  | ILMN_1698732 | PALLD | NM_016081.3 | 872.29 | 1298.23 | 1.49 |
|  | ILMN_1705301 | TEAD4 | NM_201443.1 | 1607.94 | 2399.75 | 1.49 |
|  | ILMN_1708143 | FAM127A | NM_001078171.1 | 2456.97 | 3645.75 | 1.48 |
|  | ILMN_1727080 | MYO6 | NM_004999.3 | 217.37 | 326.36 | 1.50 |
|  | ILMN_1727479 | TPRG1L | NM_182752.3 | 1473.47 | 2193.47 | 1.49 |
|  | ILMN_1732468 | HSPA4L | NM_014278.2 | 249.4 | 371.87 | 1.49 |
|  | ILMN_1751576 | TEK | NM_000459.1 | 1635.29 | 2439.44 | 1.49 |
|  | ILMN_1754795 | FAT1 | NM_005245.3 | 460.69 | 684.23 | 1.49 |
|  | ILMN_1755649 | SLC16A5 | NM_004695.2 | 185.46 | 277.63 | 1.50 |
|  | ILMN_1757237 | DDEF2 | NM_003887.1 | 1006.87 | 1496.45 | 1.49 |
|  | ILMN_1763447 | PLXNB2 | NM_012401.2 | 419.93 | 625.7 | 1.49 |
|  | ILMN_1766925 | CDH13 | NM_001257.3 | 498.23 | 742.2 | 1.49 |
|  | ILMN_1776953 | MYL9 | NM_006097.3 | 206.21 | 309.69 | 1.50 |
|  | ILMN_1795429 | VCL | NM_014000.2 | 6698.71 | 9942.09 | 1.48 |
|  | ILMN_1805773 | ART4 | NM_021071.2 | 155.92 | 233.94 | 1.50 |
|  | ILMN_1809931 | NDRG1 | NM_006096.2 | 1022.58 | 1523.67 | 1.49 |

|  | Probe ID | Symbol | Genebank accession | siControl | siSIRT6 | Fold ratio (siSIRT6/siControl) |
| --- | --- | --- | --- | --- | --- | --- |
|  | ILMN_1842286 |  | AV735490 | 139.33 | 209.29 | 1.50 |
|  | ILMN_1875123 |  | BI024234 | 1085.64 | 1619.13 | 1.49 |
|  | ILMN_2169439 | ITGAV | NM_002210.2 | 1411.16 | 2109.96 | 1.50 |
|  | ILMN_2180677 | ARMCX1 | NM_016608.1 | 844.14 | 1263.32 | 1.50 |
|  | ILMN_2225735 | CRBN | NM_016302.2 | 527.12 | 782.71 | 1.48 |
|  | ILMN_2319077 | FAS | NM_152877.1 | 174.09 | 261.62 | 1.50 |
|  | ILMN_2342033 | F11R | NM_144504.1 | 1945.6 | 2884.29 | 1.48 |
|  | ILMN_2412192 | CFH | NM_001014975.1 | 170.31 | 252.42 | 1.48 |
|  | ILMN_3178307 | KRT18P28 | XR_017689.1 | 334.69 | 497.08 | 1.49 |
|  | ILMN_3190972 | LOC100130291 | XR_038455.1 | 876.13 | 1311.8 | 1.50 |
|  | ILMN_3231638 | FAM160B1 | NM_020940.3 | 662.45 | 992.55 | 1.50 |
|  | ILMN_3250257 | ACVRL1 | NM_000020.2 | 2659.61 | 3969.29 | 1.49 |
|  | ILMN_3263974 | KRT18P13 | XM_001726959.1 | 1085.14 | 1620.25 | 1.49 |
|  | ILMN_1653115 | ECH1 | NM_001398.2 | 656.05 | 990.49 | 1.51 |
|  | ILMN_1670322 | FCHO2 | NM_138782.1 | 306.55 | 463.9 | 1.51 |
|  | ILMN_1672124 | C4orf18 | NM_016613.4 | 480.59 | 723.24 | 1.50 |
|  | ILMN_1684887 | SAMSN1 | NM_022136.3 | 205.36 | 311.27 | 1.52 |
|  | ILMN_1686254 | FAM127B | NM_001078172.1 | 812.37 | 1226.22 | 1.51 |
|  | ILMN_1696675 | CES2 | NM_003869.4 | 551.03 | 831.94 | 1.51 |
|  | ILMN_1696749 | LMNA | NM_005572.3 | 4034.95 | 6110.18 | 1.51 |
|  | ILMN_1698231 | RRM2B | NM_015713.3 | 248.83 | 374.29 | 1.50 |
|  | ILMN_1707727 | ANGPTL4 | NM_139314.1 | 764.83 | 1167.6 | 1.53 |
|  | ILMN_1711408 | ANXA4 | NM_001153.2 | 530.67 | 806.02 | 1.52 |
|  | ILMN_1715508 | NNMT | NM_006169.2 | 893.3 | 1363.41 | 1.53 |
|  | ILMN_1724533 | LY96 | NM_015364.2 | 1080.64 | 1631.52 | 1.51 |
|  | ILMN_1730906 | FILIP1L | NM_182909.2 | 197.77 | 299.97 | 1.52 |
|  | ILMN_1757631 | DBNDD1 | NM_001042610.1 | 178.49 | 270.03 | 1.51 |
|  | ILMN_1760667 | POLR3GL | NM_032305.1 | 1214.66 | 1841.08 | 1.52 |
|  | ILMN_1761833 | SLC40A1 | NM_014585.4 | 179.52 | 270.28 | 1.51 |
|  | ILMN_1769288 | LOC402560 | XM_944321.1 | 341.8 | 517.95 | 1.52 |
|  | ILMN_1775016 | MPZL2 | NM_144765.1 | 158.98 | 242.25 | 1.52 |
|  | ILMN_1795778 | P4HA2 | NM_001017973.1 | 823.71 | 1253.72 | 1.52 |
|  | ILMN_1802888 | ZNF185 | NM_007150.2 | 1601.27 | 2423.71 | 1.51 |
|  | ILMN_1803647 | FAM162A | NM_014367.3 | 1175.45 | 1779.18 | 1.51 |
|  | ILMN_1803772 | POLD4 | NM_021173.2 | 220.56 | 332.14 | 1.51 |
|  | ILMN_1804629 | TPK1 | NM_001042482.1 | 515.08 | 786.33 | 1.53 |
|  | ILMN_1808508 | KITLG | NM_000899.3 | 270.1 | 407.31 | 1.51 |
|  | ILMN_2052208 | GADD45A | NM_001924.2 | 950.36 | 1434.83 | 1.51 |
|  | ILMN_2061446 | AADACL1 | NM_020792.3 | 1850.46 | 2789.9 | 1.51 |
|  | ILMN_2076602 | ITM2A | NM_004867.3 | 191.69 | 291.97 | 1.52 |
|  | ILMN_2380967 | DNASE1L1 | NM_001009934.1 | 1383.4 | 2103.15 | 1.52 |
|  | ILMN_3243156 | AHNAK2 | NM_138420.2 | 523.12 | 789.06 | 1.51 |
|  | ILMN_1671260 | GPR177 | NM_001002292.1 | 1149.4 | 1775.49 | 1.54 |
|  | ILMN_1772286 | OCIAD2 | NM_152398.2 | 2845.89 | 4359.66 | 1.53 |
|  | ILMN_1782897 | CAPRIN1 | NM_203364.2 | 624.55 | 967.2 | 1.55 |
|  | ILMN_1790807 | XPC | NM_004628.3 | 664.75 | 1030.41 | 1.55 |

|  | Probe ID | Symbol | Genebank accession | siControl | siSIRT6 | Fold ratio (siSIRT6/siControl) |
| --- | --- | --- | --- | --- | --- | --- |
|  | ILMN_1795183 | RNASE1 | NM_198232.1 | 724.91 | 1110.76 | 1.53 |
|  | ILMN_1843198 |  | AK026966 | 206.07 | 315.9 | 1.53 |
|  | ILMN_2072101 | C4orf49 | NM_032623.3 | 1206.27 | 1860.75 | 1.54 |
|  | ILMN_2261076 | NEDD9 | NM_006403.2 | 593.05 | 913.33 | 1.54 |
|  | ILMN_2319913 | DGKA | NM_201554.1 | 205.69 | 315.03 | 1.53 |
|  | ILMN_2336094 | ODZ3 | NM_001080477.1 | 362.71 | 556.28 | 1.53 |
|  | ILMN_2356111 | SLC41A3 | NM_017836.3 | 850.4 | 1299.13 | 1.53 |
|  | ILMN_2388142 | CD99L2 | NM_134445.2 | 1278.29 | 1960.04 | 1.53 |
|  | ILMN_3201937 | LOC645381 | XR_038557.1 | 278.59 | 425.99 | 1.53 |
|  | ILMN_3236160 | C4orf49 | NM_032623.3 | 989.8 | 1514.54 | 1.53 |
|  | ILMN_1662358 | MX1 | NM_002462.2 | 189.98 | 299.07 | 1.57 |
|  | ILMN_1666976 | PLD3 | NM_001031696.1 | 723.41 | 1126.79 | 1.56 |
|  | ILMN_1702363 | SULF1 | NM_015170.1 | 157.22 | 245.91 | 1.56 |
|  | ILMN_1715607 | CHMP4A | NM_014169.2 | 773.18 | 1214.94 | 1.57 |
|  | ILMN_1729453 | TSPAN9 | NM_006675.3 | 1157.4 | 1809.03 | 1.56 |
|  | ILMN_1758281 | CALCRL | NM_005795.4 | 786.52 | 1221.41 | 1.55 |
|  | ILMN_1761566 | C5orf32 | NM_032412.3 | 556.02 | 873.3 | 1.57 |
|  | ILMN_1772722 | MRPS33 | NM_016071.2 | 237.37 | 371.79 | 1.57 |
|  | ILMN_1785424 | ABLIM1 | NM_006720.3 | 519.99 | 807.7 | 1.55 |
|  | ILMN_1796423 | CLIC3 | NM_004669.2 | 152.57 | 240.24 | 1.57 |
|  | ILMN_1802615 | CDK6 | NM_001259.5 | 482.04 | 751.35 | 1.56 |
|  | ILMN_1907834 |  | AK025332 | 271.16 | 426.58 | 1.57 |
|  | ILMN_2160210 | TACSTD1 | NM_002354.1 | 175.34 | 272.54 | 1.55 |
|  | ILMN_2171289 | SAMSN1 | NM_022136.3 | 265.89 | 417.41 | 1.57 |
|  | ILMN_2190084 | VAMP8 | NM_003761.2 | 1396.56 | 2190.94 | 1.57 |
|  | ILMN_2230892 | IL10RB | NM_000628.3 | 1192.96 | 1850.46 | 1.55 |
|  | ILMN_2336130 | SULT1A4 | NM_001017391.1 | 194.55 | 304.44 | 1.56 |
|  | ILMN_2357272 | BCLAF1 | NM_014739.2 | 489.33 | 767.31 | 1.57 |
|  | ILMN_2381899 | OPTN | NM_001008213.1 | 404.13 | 635.46 | 1.57 |
|  | ILMN_1659462 | DUSP23 | NM_017823.3 | 1634.91 | 2597.67 | 1.59 |
|  | ILMN_1671565 | RNASET2 | NM_003730.3 | 336.24 | 533.13 | 1.59 |
|  | ILMN_1720048 | CCL2 | NM_002982.3 | 323.29 | 509.17 | 1.57 |
|  | ILMN_1721732 | GSDMC | NM_031415.2 | 269.85 | 430.74 | 1.60 |
|  | ILMN_1736234 | C1orf77 | NM_015607.2 | 1141.99 | 1812.38 | 1.59 |
|  | ILMN_1765641 | SEMA3A | NM_006080.2 | 255.88 | 405.06 | 1.58 |
|  | ILMN_1770290 | CNN2 | NM_201277.1 | 2328.74 | 3693.23 | 1.59 |
|  | ILMN_1811702 | GRN | NM_002087.2 | 782.89 | 1249.38 | 1.60 |
|  | ILMN_2170595 | RRM2B | NM_015713.3 | 581.51 | 926.3 | 1.59 |
|  | ILMN_2387078 | MPZL2 | NM_144765.1 | 183.46 | 292.58 | 1.59 |
|  | ILMN_1656057 | PLAU | NM_002658.2 | 1502.69 | 2431 | 1.62 |
|  | ILMN_1657111 | C14orf78 | XM_001132404.1 | 846.09 | 1367.19 | 1.62 |
|  | ILMN_1658494 | C13orf15 | NM_014059.2 | 1515.94 | 2433.25 | 1.61 |
|  | ILMN_1659047 | HIST2H2AA3 | NM_003516.2 | 248.14 | 402.54 | 1.62 |
|  | ILMN_1669376 | DRAM1 | NM_018370.2 | 501 | 803.41 | 1.60 |
|  | ILMN_1704753 | EPAS1 | NM_001430.3 | 812.37 | 1318.79 | 1.62 |
|  |  |  |  |  |  |  |

|  | Probe ID | Symbol | Genebank accession | siControl | siSIRT6 | Fold ratio (siSIRT6/siControl) |
| --- | --- | --- | --- | --- | --- | --- |
|  | ILMN_1716988 | OPN3 | NM_014322.2 | 403.85 | 653.93 | 1.62 |
|  | ILMN_1758626 | IDS | NM_000202.3 | 621.24 | 994.39 | 1.60 |
|  | ILMN_2365569 | ICA1 | NM_004968.2 | 361.12 | 579.77 | 1.61 |
|  | ILMN_2381697 | P4HA2 | NM_001017974.1 | 524.09 | 840.63 | 1.60 |
|  | ILMN_3277209 | LOC285943 | XR_040116.1 | 306.48 | 491.6 | 1.60 |
|  | ILMN_1652409 | SPATA7 | NM_001040428.2 | 293.46 | 484.38 | 1.65 |
|  | ILMN_1668514 | PIP5K1C | NM_012398.1 | 465.4 | 762.01 | 1.64 |
|  | ILMN_1684746 | IPO11 | NM_016338.3 | 2089.1 | 3414.2 | 1.63 |
|  | ILMN_1698706 | NID2 | NM_007361.3 | 298.1 | 491.14 | 1.65 |
|  | ILMN_1738578 | FILIP1L | NM_014890.2 | 194.42 | 320.61 | 1.65 |
|  | ILMN_1741356 | PRICKLE1 | NM_153026.1 | 951.68 | 1558.56 | 1.64 |
|  | ILMN_1757019 | NTN4 | NM_021229.2 | 222.81 | 365.4 | 1.64 |
|  | ILMN_1759513 | RND3 | NM_005168.3 | 1511.75 | 2473.49 | 1.64 |
|  | ILMN_1761941 | C4orf18 | NM_016613.5 | 1277.7 | 2080.43 | 1.63 |
|  | ILMN_1769299 | MTMR11 | NM_181873.2 | 365.91 | 600.49 | 1.64 |
|  | ILMN_1769615 | FLRT2 | NM_013231.4 | 279.95 | 459.21 | 1.64 |
|  | ILMN_1776188 | MAP1LC3A | NM_181509.1 | 779.46 | 1286.29 | 1.65 |
|  | ILMN_1780825 | RRAS | NM_006270.3 | 2136.45 | 3490.78 | 1.63 |
|  | ILMN_1791792 | C12orf5 | NM_020375.2 | 749.27 | 1226.78 | 1.64 |
|  | ILMN_1796179 | HIST1H2BK | NM_080593.1 | 1362.46 | 2230.27 | 1.64 |
|  | ILMN_1814305 | SAMD9 | NM_017654.2 | 269.29 | 439.59 | 1.63 |
|  | ILMN_2066151 | TEK | NM_000459.2 | 1755.91 | 2880.29 | 1.64 |
|  | ILMN_2144426 | HIST2H2AA3 | NM_003516.2 | 166.42 | 273.49 | 1.64 |
|  | ILMN_1653466 | HES4 | NM_021170.2 | 375.59 | 623.68 | 1.66 |
|  | ILMN_1667199 | SQRDL | NM_021199.2 | 183.67 | 304.86 | 1.66 |
|  | ILMN_1669390 | PPP1R13L | NM_006663.2 | 372.99 | 619.23 | 1.66 |
|  | ILMN_1682567 | CCDC106 | NM_013301.2 | 496.85 | 824.86 | 1.66 |
|  | ILMN_1725314 | GBP3 | NM_018284.2 | 360.87 | 605.79 | 1.68 |
|  | ILMN_1759787 | THBD | NM_000361.2 | 162.5 | 268.91 | 1.65 |
|  | ILMN_1803788 | LGALS3 | NM_002306.1 | 899.3 | 1507.91 | 1.68 |
|  | ILMN_2062468 | IGFBP7 | NM_001553.1 | 2521.97 | 4220.89 | 1.67 |
|  | ILMN_2358919 | TP53I3 | NM_147184.1 | 866.27 | 1450.5 | 1.67 |
|  | ILMN_2364674 | TRPT1 | NM_031472.2 | 777.84 | 1301.24 | 1.67 |
|  | ILMN_2412139 | CABYR | NM_153768.1 | 276.92 | 459.21 | 1.66 |
|  | ILMN_1662419 | COX7A1 | NM_001864.2 | 539.94 | 911.02 | 1.69 |
|  | ILMN_1663281 | ZNF702P | NR_003578.1 | 355.33 | 604.67 | 1.70 |
|  | ILMN_1668863 | LYPD1 | NM_144586.5 | 2021.21 | 3443.52 | 1.70 |
|  | ILMN_1672661 | SP110 | NM_004510.2 | 298.52 | 504.14 | 1.69 |
|  | ILMN_1735930 | KLF2 | NM_016270.2 | 3214.4 | 5467.5 | 1.70 |
|  | ILMN_1816342 |  | CR612552 | 1009.9 | 1719.77 | 1.70 |
|  | ILMN_2054019 | ISG15 | NM_005101.1 | 582.32 | 987.98 | 1.70 |
|  | ILMN_2098616 | C5orf39 | NM_001014279.1 | 639.59 | 1090.67 | 1.71 |
|  | ILMN_2211122 | TRAPPC2P1 | NR_002166.1 | 259.15 | 440.09 | 1.70 |
|  | ILMN_2341595 | KITLG | NM_000899.3 | 618.66 | 1048.66 | 1.70 |
|  | ILMN_3307906 | PALMD | NM_017734.4 | 611.13 | 1037.81 | 1.70 |
|  | ILMN_1669982 | CCDC85A | NM_001080433.1 | 505.65 | 873.7 | 1.73 |

|  | Probe ID | Symbol | Genebank accession | siControl | siSIRT6 | Fold ratio (siSIRT6/siControl) |
| --- | --- | --- | --- | --- | --- | --- |
|  | ILMN_1680693 | ZNF419 | NM_001098495.1 | 275.58 | 473.1 | 1.72 |
|  | ILMN_1716687 | TPM1 | NM_001018020.1 | 1902.48 | 3301.72 | 1.74 |
|  | ILMN_1731107 | CCDC92 | NM_025140.1 | 650.77 | 1128.87 | 1.73 |
|  | ILMN_1732609 | KIAA1539 | NM_025182.2 | 492.51 | 842.38 | 1.71 |
|  | ILMN_1741219 | RRAGB | NM_006064.3 | 227.96 | 392.44 | 1.72 |
|  | ILMN_1781514 | PCDH17 | NM_014459.2 | 199.05 | 342.98 | 1.72 |
|  | ILMN_1791280 | HSPB8 | NM_014365.2 | 410.62 | 711.47 | 1.73 |
|  | ILMN_1800626 | SESN1 | NM_014454.1 | 276.47 | 476.06 | 1.72 |
|  | ILMN_2076600 | ITM2A | NM_004867.3 | 496.96 | 863.87 | 1.74 |
|  | ILMN_2094266 | HES2 | NM_019089.3 | 354.51 | 608.45 | 1.72 |
|  | ILMN_2188722 | GLS | NM_014905.2 | 511.05 | 888.57 | 1.74 |
|  | ILMN_3242105 | LOC100134073 | XM_001718050.1 | 778.92 | 1334.11 | 1.71 |
|  | ILMN_3308335 | RNU6-1 | NR_004394.1 | 1655.06 | 2857.1 | 1.73 |
|  | ILMN_3310351 | RNU6-15 | NR_028372.1 | 1742.97 | 3008.86 | 1.73 |
|  | ILMN_1654262 | ZMAT3 | NM_152240.1 | 1942.01 | 3386.7 | 1.74 |
|  | ILMN_1657977 | MSRB2 | NM_012228.2 | 1017.87 | 1791.15 | 1.76 |
|  | ILMN_1701424 | LAMC2 | NM_005562.1 | 189.93 | 335.46 | 1.77 |
|  | ILMN_1717990 | CALD1 | NM_033138.2 | 189.8 | 334.69 | 1.76 |
|  | ILMN_1769520 | UBE2L6 | NM_004223.3 | 346.49 | 609.3 | 1.76 |
|  | ILMN_1797793 | BLVRB | NM_000713.1 | 704.11 | 1225.37 | 1.74 |
|  | ILMN_1805665 | FLRT3 | NM_198391.1 | 150.99 | 266.56 | 1.77 |
|  | ILMN_1814787 | ICA1 | NM_004968.2 | 621.81 | 1090.17 | 1.75 |
|  | ILMN_2067656 | CCND2 | NM_001759.2 | 357.22 | 621.52 | 1.74 |
|  | ILMN_2360710 | TPM1 | NM_001018004.1 | 2268.2 | 3986.76 | 1.76 |
|  | ILMN_2399769 | GPR177 | NM_001002292.1 | 745.12 | 1302.44 | 1.75 |
|  | ILMN_1651958 | MGP | NM_000900.2 | 478.27 | 861.08 | 1.80 |
|  | ILMN_1673639 | ABI3BP | NM_015429.2 | 184.52 | 331 | 1.79 |
|  | ILMN_1694847 | TERF1 | NM_017489.1 | 338.34 | 603.13 | 1.78 |
|  | ILMN_1709634 | CMBL | NM_138809.3 | 731.65 | 1301.24 | 1.78 |
|  | ILMN_2113490 | NTN4 | NM_021229.3 | 464.11 | 833.67 | 1.80 |
|  | ILMN_2342579 | IL7R | NM_002185.2 | 214.92 | 386.59 | 1.80 |
|  | ILMN_2400935 | TAGLN | NM_003186.3 | 120.37 | 214.13 | 1.78 |
|  | ILMN_2415144 | SP110 | NM_004510.2 | 283.53 | 503.67 | 1.78 |
|  | ILMN_1688630 | RECK | NM_021111.1 | 267.98 | 490.35 | 1.83 |
|  | ILMN_1716407 | SORBS2 | NM_003603.4 | 208.8 | 378.99 | 1.82 |
|  | ILMN_1748206 | C20orf160 | NM_080625.2 | 216.72 | 394.44 | 1.82 |
|  | ILMN_1659106 | PHLDA3 | NM_012396.3 | 280.2 | 517.71 | 1.85 |
|  | ILMN_1683023 | PDGFC | NM_016205.1 | 332.91 | 619.37 | 1.86 |
|  | ILMN_1708728 | H2AFJ | NM_177925.2 | 1275.05 | 2357.43 | 1.85 |
|  | ILMN_1819608 |  | AL157484 | 469.72 | 873.7 | 1.86 |
|  | ILMN_2150175 | GDF6 | NM_001001557.1 | 167.85 | 313.65 | 1.87 |
|  | ILMN_2333670 | RNASE1 | NM_198235.1 | 1574.85 | 2916.45 | 1.85 |
|  | ILMN_2407879 | SORBS2 | NM_003603.4 | 251.02 | 463.36 | 1.85 |
|  | ILMN_3229189 | N4BP2L2 | NM_033111.3 | 155.56 | 287.02 | 1.85 |
|  | ILMN_1656369 | C8orf4 | NM_020130.3 | 147.68 | 280.92 | 1.90 |
|  | ILMN_1691341 | IL7R | XM_937367.1 | 326.51 | 621.09 | 1.90 |

|  | Probe ID | Symbol | Genebank accession | siControl | siSIRT6 | Fold ratio (siSIRT6/siControl) |
| --- | --- | --- | --- | --- | --- | --- |
|  | ILMN_1730777 | KRT19 | NM_002276.3 | 735.88 | 1383.72 | 1.88 |
|  | ILMN_1752579 | ATP6V0A1 | NM_005177.3 | 755 | 1416.39 | 1.88 |
|  | ILMN_1771482 | KIAA1324 | NM_020775.2 | 171.14 | 320.31 | 1.87 |
|  | ILMN_1671703 | ACTA2 | NM_001613.1 | 571.13 | 1105.13 | 1.93 |
|  | ILMN_1717793 | C19orf33 | NM_033520.1 | 187.27 | 359.54 | 1.92 |
|  | ILMN_1731418 | SP110 | NM_004510.2 | 336.78 | 642.7 | 1.91 |
|  | ILMN_1680110 | C10orf116 | NM_006829.2 | 713.45 | 1389.48 | 1.95 |
|  | ILMN_1721770 | PAPPA | NM_002581.3 | 170.39 | 336.08 | 1.97 |
|  | ILMN_2071809 | MGP | NM_000900.2 | 619.52 | 1218.87 | 1.97 |
|  | ILMN_2324002 | CALD1 | NM_033157.2 | 919.48 | 1792.8 | 1.95 |
|  | ILMN_2374352 | DBNDD1 | NM_001042610.1 | 1029.46 | 2036.67 | 1.98 |
|  | ILMN_1701114 | GBP1 | NM_002053.1 | 250.91 | 498.34 | 1.99 |
|  | ILMN_1784602 | CDKN1A | NM_000389.2 | 3436.36 | 6904.56 | 2.01 |
|  | ILMN_1865764 |  | BC036485 | 1393.66 | 2791.19 | 2.00 |
|  | ILMN_2063168 | MALL | NM_005434.3 | 3389.05 | 6798.5 | 2.01 |
|  | ILMN_1672611 | CDH11 | NM_001797.2 | 968.99 | 1975.5 | 2.04 |
|  | ILMN_1674650 | C9orf95 | NM_017881.1 | 306.13 | 622.67 | 2.03 |
|  | ILMN_1677200 | CYFIP2 | NM_014376.2 | 376.98 | 771.58 | 2.05 |
|  | ILMN_2053103 | SLC40A1 | NM_014585.3 | 230.24 | 467.67 | 2.03 |
|  | ILMN_1682034 | HEY2 | NM_012259.1 | 254.82 | 534.61 | 2.10 |
|  | ILMN_1699226 | UBR4 | NM_020765.2 | 868.27 | 1814.89 | 2.09 |
|  | ILMN_1774077 | GBP2 | NM_004120.3 | 285.43 | 589.09 | 2.06 |
|  | ILMN_1660549 | GPR177 | NM_001002292.1 | 991.87 | 2107.52 | 2.12 |
|  | ILMN_1685433 | COL8A1 | NM_020351.2 | 1749.83 | 3714.62 | 2.12 |
|  | ILMN_1769876 | TBC1D2 | NM_018421.2 | 313.5 | 671.54 | 2.14 |
|  | ILMN_2163723 | KRT7 | NM_005556.3 | 590.86 | 1263.32 | 2.14 |
|  | ILMN_1683263 | TSPAN8 | NM_004616.2 | 313.29 | 684.23 | 2.18 |
|  | ILMN_1757338 | PLSCR4 | NM_020353.1 | 814.06 | 1803.6 | 2.22 |
|  | ILMN_1713124 | AKR1C3 | NM_003739.4 | 457.2 | 1045.52 | 2.29 |
|  | ILMN_2402392 | COL8A1 | NM_020351.2 | 1226.5 | 2795.71 | 2.28 |
|  | ILMN_1694548 | ANXA3 | NM_005139.2 | 1805.69 | 4254.18 | 2.36 |
|  | ILMN_2148785 | GBP1 | NM_002053.1 | 556.15 | 1370.35 | 2.46 |
|  | ILMN_1653026 | PLAC8 | NM_016619.1 | 185.46 | 466.37 | 2.51 |
|  | ILMN_2067269 | RECK | NM_021111.1 | 607.61 | 1531.79 | 2.52 |
|  | ILMN_2058782 | IFI27 | NM_005532.3 | 1293.44 | 3305.53 | 2.56 |
|  | ILMN_1752932 | MPZL2 | NM_005797.2 | 788.88 | 2170.78 | 2.75 |
|  | ILMN_1685917 | EMCN | NM_016242.2 | 392.62 | 1183.08 | 3.01 |
|  | ILMN_2093343 | PLAC8 | NM_016619.1 | 224.41 | 692.18 | 3.08 |
|  | ILMN_1778668 | TAGLN | NM_003186.3 | 207.36 | 751.87 | 3.63 |
